# Adaptively evolved human oral actinomyces‐sourced defensins show therapeutic potential

**DOI:** 10.15252/emmm.202114499

**Published:** 2021-12-20

**Authors:** Shunyi Zhu, Bin Gao, Yoshitaka Umetsu, Steve Peigneur, Ping Li, Shinya Ohki, Jan Tytgat

**Affiliations:** ^1^ Group of Peptide Biology and Evolution State Key Laboratory of Integrated Management of Pest Insects and Rodents Institute of Zoology Chinese Academy of Sciences Beijing China; ^2^ Center for Nano Materials and Technology (CNMT) Japan Advanced Institute of Science and Technology (JAIST) Nomi Japan; ^3^ Toxicology and Pharmacology University of Leuven Leuven Belgium; ^4^ Key Laboratory for Biomedical Effects of Nanomaterials and Nanosafety (Chinese Academy of Sciences) National Center for Nanoscience and Technology Beijing China

**Keywords:** actinomycesin, adaptive evolution, antimicrobial peptide, cell‐wall synthesis inhibitor, systemic therapy, Microbiology, Virology & Host Pathogen Interaction

## Abstract

The development of eukaryote‐derived antimicrobial peptides as systemically administered drugs has proven a challenging task. Here, we report the first human oral actinomyces‐sourced defensin—actinomycesin—that shows promise for systemic therapy. Actinomycesin and its homologs are only present in actinobacteria and myxobacteria, and share similarity with a group of ancient invertebrate‐type defensins reported in fungi and invertebrates. Signatures of natural selection were detected in defensins from the actinomyces colonized in human oral cavity and ruminant rumen and dental plaque, highlighting their role in adaptation to complex multispecies bacterial communities. Consistently, actinomycesin exhibited potent antibacterial activity against oral bacteria and clinical isolates of *Staphylococcus* and synergized with two classes of human salivary antibacterial factors. Actinomycesin specifically inhibited bacterial peptidoglycan synthesis and displayed weak immunomodulatory activity and low toxicity on human and mammalian cells and ion channels in the heart and central nervous system. Actinomycesin was highly efficient in mice infected with *Streptococcus pneumoniae* and mice with *MRSA*‐induced experimental peritoneal infection. This work identifies human oral bacteria as a new source of systemic anti‐infective drugs.

## Graphical abstract

Bacteria are a major source of clinically used antibiotics. This study explored the potential of oral actinomyces‐sourced defensins as systemic anti‐infective drugs via a combination of microbial genome mining, evolution analysis and experimental studies.

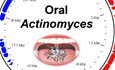

**The paper explained**

**Problem**

Despite their evolutionary success as eukaryotic defensive weapon across 1.8‐billion‐year history and intense research over several decades, few eukaryote‐sourced antimicrobial peptides (AMPs) have reached the stage of clinical applications for treating systemic infection. Two main intrinsic factors hamper the development of these AMPs as systemically administered drugs. First, in eukaryotes, especially in multicellular organisms, most AMPs defend a microenvironment and are not delivered into the systemic circulation; second, in multicellular organisms, AMPs have evolved multiple biological functions beyond their antibiotic activity. These additional properties often interfere with the human immune system function when administered systemically.

**Results**

Our study addressed these two critical issues based on the hypothesis that AMPs from unicellular, prokaryotic organisms that are the most evolutionary distant from humans, may exhibit more advantages than AMPs from multicellular organisms. We searched for AMPs from the public available microbial genome resources and discovered AMSIN, the first human oral actinomyces‐sourced defensin targeting bacterial cell‐wall biosynthesis and exhibiting therapeutic potential against bacterial infections elicited by *Streptococcus pneumoniae* and methicillin‐resistant *Staphylococcus aureus* in mice.

**Impact**

This work identifies human oral bacteria as a new source of peptide antibiotics, and might stimulate a shift in this field of research from eukaryotes to prokaryotes.

## Introduction

The extensive use of conventional antibiotics has led to the emergence of antimicrobial resistance that is spreading faster than the development of new antibiotics, posing a great medical challenge in the 21^st^ century (Ling *et al*, 2015; Sierra *et al*, 2017). To tackle this challenge, one needs to explore various new sources of antibiotics with diverse chemical structures and modes of action to delay the occurrence of resistance. As the effectors of the innate immunity of multicellular organisms, antimicrobial peptides (AMPs), also known as peptide antibiotics (Hancock, 1997), provide the first line of defense against microbial infection. Because of their evolutionary success as a defensive weapon of multicellular organisms across 1.8‐billion‐year history (Futuyma & Kirkpatrick, 2017), these molecules have been considered as a class of promising alternatives of conventional anti‐infective agents (Zasloff, 2002, 2019; Fox, 2013; Mylonakis *et al*, 2016; Magana *et al*, 2020). AMPs are a class of cationic molecules of normally < 100 amino acids and broadly present in plants, fungi, and animals (Mygind *et al*, 2005; Fjell *et al*, 2012; Mylonakis *et al*, 2016; Sierra *et al*, 2017). Their antimicrobial activity is mostly associated with their ability to electrostatically attach to an accessible bacterial membrane with a strong negatively charged surface and some are found to bind to other bacterial components (e.g., Lipid II and intracellular targets) (Brogden, 2005; Oppedijk *et al*, 2016). Besides merely serving as endogenous peptide antibiotics (Hancock, 1997), AMPs in vertebrates (also known as host defense peptides, HDPs) also function as multifaceted modulating molecules involved in both the innate and adaptive immune responses (Mansour *et al*, 2014).

Despite intense studies over several decades, very few AMPs have reached to the stage of clinical applications to treat systemic infection (Fox, 2013; Zasloff, 2019; Magana *et al*, 2020). This frustration is mainly because of the intrinsic non‐specific cytotoxicity stemmed from their fundamental detergent‐like properties causing membrane (particularly erythrocytes) disruption, in a dose required for treatment (Gao *et al*, 2009, 2018; Vaara, 2009; Mansour *et al*, 2014; Zasloff, 2019; Mahlapuu *et al*, 2020). In addition, human and other vertebrate‐derived AMPs often exhibit multifaceted modulating properties that can interfere with the human immune system function when used as systemically administered drugs (Vaara, 2009; Mansour *et al*, 2014; Zasloff, 2019). Alternatively, AMPs from unicellular, prokaryotic organisms that have the most remote evolutionary distance from humans may exhibit more advantages in this aspect (Vaara, 2009). Firstly, most naturally occurring antibiotics clinically available are produced by actinomycetes and myxobacteria, the two most important natural sources of active metabolites, and only very few by fungi (Geddes, 2008; Waksman *et al*, 2010; Wright, 2012). Secondly, no multicellular organisms‐sourced AMPs have been clinically approved for systemic therapy and several animal‐sourced AMPs show a promise only for topical therapy (Zasloff, 2002, 2019). This may be explained by the fact that most of these AMPs are evolved to defend a microenvironment (e.g., skin, oral cavity, and eyes, etc.) and not to be delivered into the systemic circulation (Zasloff, 2019). Indeed, it has been reported that human serum and blood proteins as well as fetal bovine serum (FBS) are able to inactivate the antimicrobial activity of human HDPs, for example, LL‐37 and defensins (Panyutich *et al*, 1995; Johansson *et al*, 1998; Kudryashova *et al*, 2021). This property might become an intrinsic cause bedeviling them as systemically administered drugs. Thirdly, like bacteria‐produced chemical antibiotics (Hibbing *et al*, 2010), AMPs in bacteria should be evolved to fight their producers’ competitors (i.e., antagonism) as “pure” peptide antibiotics other than to optimize an inherent property to interfere with the human immune system. We, thus, inferred that exploring bacteria‐derived AMPs might be a more promising strategy than using currently available AMPs from multicellular organisms.

To confirm this, we used defensins as an example to exploit the structural diversity and therapeutic potential of bacterial AMPs. Defensins are a class of small cationic AMPs stabilized by multiple intramolecular disulfide bridges. Based on the connectivity and orientation of their disulfide bridges, defensins are categorized into two distinct structural classes: *cis*‐ and *trans*‐defensins (Shafee *et al*, 2016). The former includes peptides with the cysteine‐stabilized α‐helix and β‐sheet (CSαβ) motif and is also the only AMPs shared by plants, fungi, and invertebrates (Dimarcq *et al*, 1998; Mygind *et al*, 2005; Zhu *et al*, 2005), which can be further classified into three major subgroups: ancient invertebrate‐type defensins (AITDs); classical insect‐type defensins (CITDs); and plant/insect‐type defensins (PITDs) (Zhu, 2008). The latter includes vertebrate‐derived α‐, β‐, θ‐defensins and the big defensins from invertebrates (mollusks, arthropods, and chordates) (Shafee *et al*, 2016). Evolutionarily, they both could originate from a common ancestor (Zhou *et al*, 2019).

By surveying the microbial genomes, we found that AITD‐like peptides are restrictedly distributed in two microbial subgroups (actinobacteria and myxobacteria). On this basis, we measured the selective pressure driving the evolution of bacterial defensins by codon‐substitution models and conducted a series of experiments to determine the structure of actinomycesin (abbreviated as AMSIN), one representative of the actinomyces‐sourced defensins, and to evaluate its antibacterial activity, action mode, and interacting target as well as therapeutic potential. Our work reveals that: (i) Different from the defensins from fungi and animals, defensins from the actinomyces colonized in human oral cavity as well as ruminant rumen and dental plaque are evolving under positive Darwinian selection, in favor of their role in ecological adaptation to the complex oral and rumen multispecies communities. (ii) AMSIN is an inhibitor of cell‐wall synthesis with a significant therapeutic efficacy on the systemic infections caused by *Streptococcus pneumoniae* (abbreviated as *SP*) and methicillin‐resistant *Staphylococcus aureus* (abbreviated as *MRSA*) in two experimental mouse models. Our studies indicate that AMSIN and its bacterial homologs from actinobacteria and myxobacteria represent a new source of peptide antibiotics for systemic therapy.

## Results

### Discovery and evolution of bacterial defensins

**Figure EV1:**
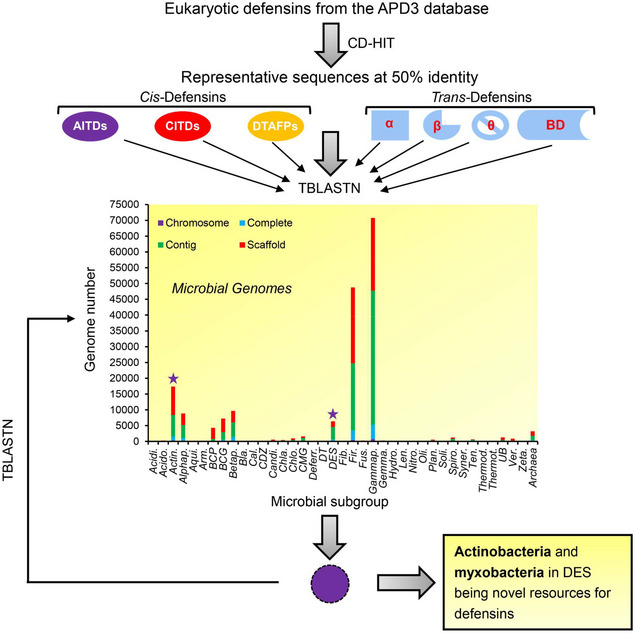
Database search for discovery of bacterial homologs of eukaryotic defensins Queries extracted from the APD3 database included *cis*‐ and *trans*‐defensins (Shafee *et al*, 2016; Zhou *et al*, 2019) and their redundancy was removed by CD‐HIT to 50% identity. The sign “★” denotes the microbial subgroups identified as the sources of bacterial defensin‐like peptides. “Actin.” and “DES” are the abbreviations of actinobacteria and delta/epsilon subdivisions, respectively. For other abbreviations, see Dataset EV1.

**Figure 1:**
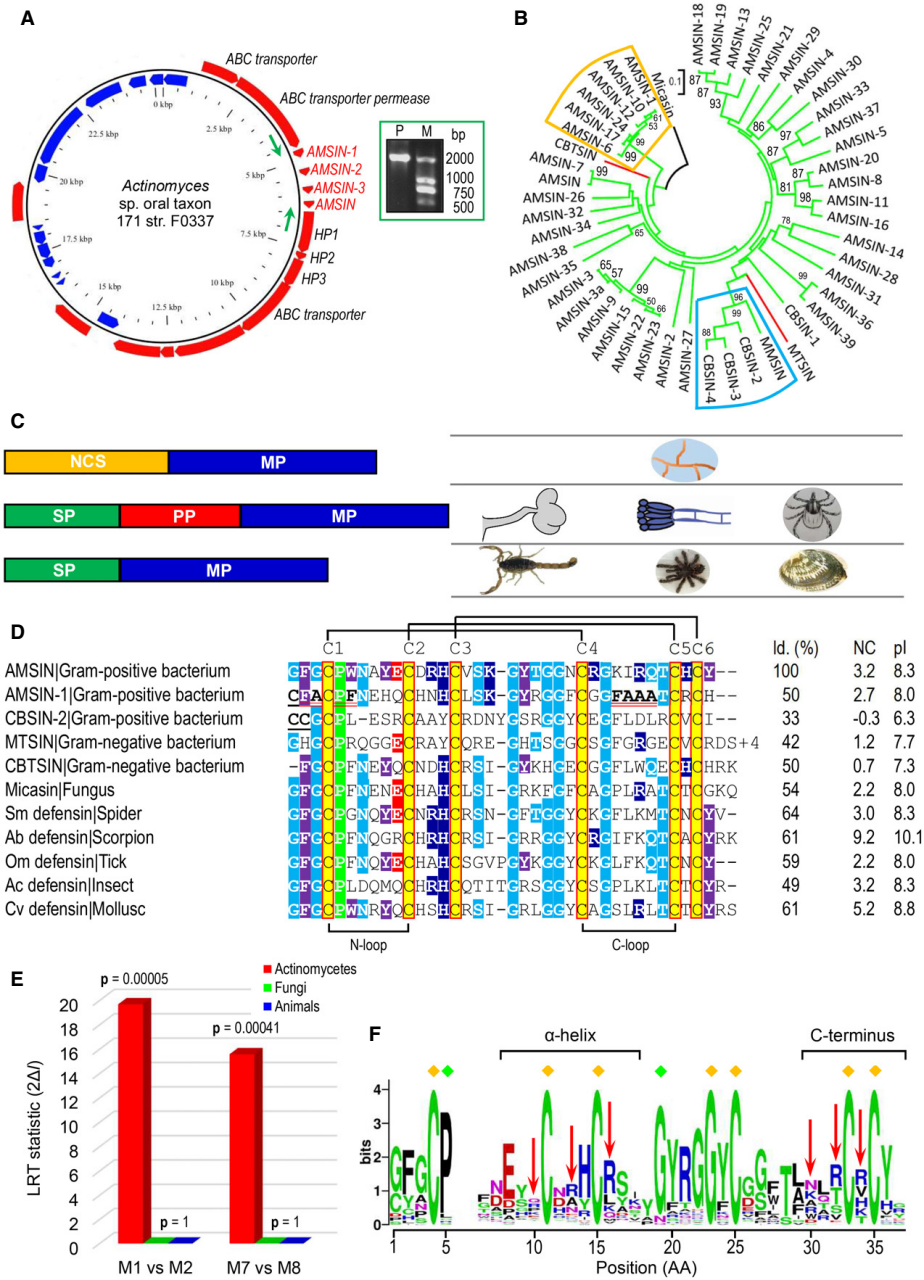
Comparisons of AITDs from prokaryotic and eukaryotic organisms Circular genome plot for *Actinomyces* sp. oral taxon 171 str. F0337 with genes encoding AITD‐like peptides. In this GView (Petkau *et al*, 2010) image, red and blue tracks show genes on forward and reverse strands, respectively. AMSIN genes and their immediate upstream and downstream genes are shown. ABC, ATP‐binding cassette. HP, hypothetical protein. The BioProject database accession number for the genomes is PRJNA43131. The AMSIN gene cluster was verified by PCR amplification (inset) and DNA sequencing. Arrows in *green* mark binding positions of the two specific primers.A neighbor‐joining tree of bacterial defensins with the fungal defensin micasin as outgroup (Zhu *et al*, 2012). The evolutionary distances were computed by the *p*‐distance method. Numbers on the nodes represent bootstrap values (from 500 replicates) and only > 50% are shown. The scale bar represents the number of amino acid differences per site. Branches in green and red indicate peptides from actinobacteria and myxobacteria, respectively. Two clusters comprising members with structural variations are boxed in different colors (orange, dimer; cyan, vicinal disulfide).Comparison of precursor organization of AITDs with different origins. NCSS, non‐classical signal sequence; SP, signal peptide; PP, propeptide; MP, mature peptide.Sequence comparison between unicellular and multicellular AITDs. For the latter, only the top BLASTP hits obtained by AMSIN as query searching against the GenBank database were chosen for alignment. The completely conserved cysteines are shaded in yellow and the non‐conserved underlined once. Identical non‐cysteine residues are shaded in different colors based on their side‐chain nature (red, acidic; purple, aromatic; blue, basic; cyan, polar and uncharged; green, hydrophobic). Residues in the N‐terminus and the C‐loop of AMSIN‐1, which form a continuous hydrophobic surface in its dimer, are underlined twice in red.LRT statistics for M1/M2 (df = 2) and M7/M8 (df = 2) and the corresponding tail probability *P*.Weblogo of bacterial defensins showing the evolutionary conservation (the sign ♦ in *yellow* and *green*, respectively, denoting completely and highly conserved positions). Positions under positive selection are denoted by red arrows and their structural location is indicated.

To explore potential defensin‐like peptides in bacteria, we conducted a systematic mining of the microbial genome database using eukaryotic defensins as queries (Fig EV1). The database contained 39 microbial subgroups (Dataset EV1) with genome assembly at four different levels, from which we identified 48 defensin‐like peptides. These peptides were named according to their origins at the genus level with an ending as “sin” considering their potential antibiotic feature (Dataset EV2). They are restrictedly distributed in actinobacteria and myxobacteria (Fig 1A and B; Appendix Figs S1 and S2; Datasets EV2 and EV3). The former includes *Actinomyces*, *Corynebacterium*, and *Micromonospora* and the latter includes *Melittangium* and *Cystobacter*. In each *Actinomyces* species, multiple defensin genes are clustered together in chromosomes and located between two *ABC transporter* genes (Fig 1A; Appendix Fig S1). Copy number variation, a mechanism broadly contributing to both virus evolution (Bayer *et al*, 2018) and pesticide resistance in mosquitos (Weetman *et al*, 2018), occurs in the genome of *Actinomyces oris* among different strains (MMRCO6‐1, S24V, and CCUG 34286) (Appendix Fig S1). These bacterial peptides exist as a precursor form and their organization varies depending on their origins (Fig 1C). For the actinobacteria‐derived peptides, their maturation most requires excision at an acidic residue (Glu or Asp) and few at the polar Asn (Appendix Fig S2), suggesting that they belong to a class of non‐classically secreted proteins, as evidenced by their SecP score exceeding ≥ 0.5 (Appendix Fig S3A) (Bendtsen *et al*, 2004). For the myxobacteria‐derived peptides, their precursors share the same organization as those from fungi and ticks but different from those from scorpions, spiders, and mussels (Fig 1C). In comparison with the eukaryotic defensins, these mature bacterial peptides share detectable to high sequence similarity to AITDs from fungi and invertebrates, both having a relatively short N‐loop between Cys1 and Cys2 (Fig 1D; Appendix Fig S2). At the physiological pH, 80% mature bacterial defensins carry net positive charges (Appendix Fig S3B; Dataset EV3), a typical feature required for the antibacterial function in eukaryotic defensins. Several defensin pseudogenes have also been identified in *Corynebacterium* and *Actinomyces*, in which mutations lead to a frame shift or a premature stop codon formation (Dataset EV4).

**Figure EV2:**
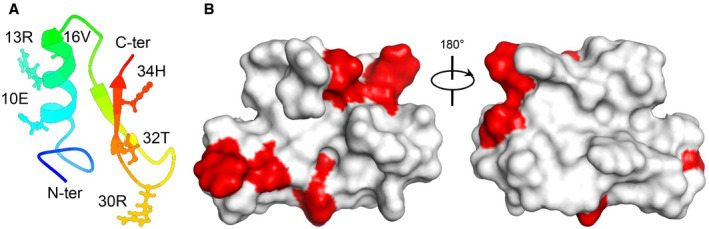
Mapping of PSSs of the actinomyces‐sourced defensins on the structure of AMSIN The ribbon drawing of AMSIN with PSSs shown as ball‐and‐stick model.The molecular surface of AMSIN. PSSs are marked in red.

As shown in Dataset EV2, most bacterial defensins described here are derived from *Actinomyces* colonized in human oral cavity (*A. johnsonii*, *A. naeslundii*, *A. oris*, and *Actinomyces* sp. oral taxon 171 str. F0337) and a few in ruminant rumen (*A*. *succiniciruminis*) and dental plaque (*A. denticolens*). Therefore, it is logical to assume that these complex multispecies bacterial communities (Kolenbrander & Periasamy, 2011; McCann *et al*, 2017; Verma *et al*, 2018; Chevrette & Currie, 2019) have driven the adaptive evolution of these defensins, which may be different from the evolution of AITDs in fungi and invertebrates. To test this assumption, we compared selective pressure for driving their evolution using the maximum likelihood‐based models of codon substitutions (Yang, 2006). For the actinomycetes‐derived defensins, the likelihood ratio test (LRT) statistic between models M1 and M2 is 2Δ*l* = 19.68915, much greater than χ^2^ distribution critical values (df = 2, *P* = 0.00005). The maximum likelihood estimate of parameters (MLEs) under M2 suggests that about 20% of sites are under positive selection with *ω* = 3.17. The test with models M7 and M8 yielded 2Δ*l* = 15.587266 (df = 2, *P* = 0.00041) (Fig 1E; Table 1). MLEs under M8 suggests that about 18% of sites are under positive selection with *ω* = 2.49 (Table 1). Both tests obtained very similar conclusions. Using the empirical Bayes method computing the posterior probabilities (*P*), six common positively selected sites (PSSs) with *P* > 95% under M2 and M8 were identified (Table 1). These sites are distributed in two evolutionarily variable regions (the α‐helix and the C‐terminus) (Fig 1F) and they all are located on the molecular surface (Fig EV2), suggesting that natural selection has intensified the antibiotic activity of these bacterial peptides through adjusting and optimizing their bacterium‐contacting surface, and thus highlighting their potential value as peptide antibiotics. On the contrary, no signatures of natural selection were detected in the fungal and animal defensins (both *P* = 1) (Fig 1E; Tables EV1 and EV2). This differential might reflect a difference in their physiological roles to the hosts. Our observations also hint at the possible involvement of these bacterial peptides in species competition as the consequence of the actinomycetes adapting to the complex oral and rumen microflora, a phenomenon analogous to the soil bacteria‐produced antibiotics (Hibbing *et al*, 2010).

**Table 1:** Maximum likelihood estimates of parameters and sites inferred to be under positive selection in the actinomyces‐sourced defensins.

Model	S	*p*	*l*	Estimates of parameters	PSSs
M0 (one‐ratio)	15.03	1	−1,545.53	*ω* = 0.64	None
M1 (Nearly Neutral)	16.65	2	−1,473.64	*p*_0_ = 0.37 (*p*_1_ = 0.63)	Not allowed
				*ω*_0_ = 0.07 (*ω*_1_ = 1.00)	
M2 (Positive Selection)	17.40	4	−1,463.80	*p*_0_ = 0.22	10E**, 13R**, 16V*
				*p*_1_ = 0.58 (*p*_2_ = 0.20)	30R*, 32T*, 34H**
				*ω*_0_ = 0.00	
				*ω*_1_ = 1.00 (*ω*_2_ = **3.17**)	
M7 (beta)	17.18	2	−1,465.13	*P* = 0.24, *q* = 0.21	Not allowed
M8 (beta&ω > 1)	17.63	4	−1,457.34	*P* = 0.18, *q* = 0.14	10E**, 13R**, 16V**
				*p*_0_ = 0.82 (*p*_1_ = 0.18)	30R**, 32T*, 34H**
				*ω*_s_ = **2.49**	

S represents the tree length; *p* is the number of parameters in the ω distribution; *l* is the log likelihood. PSSs under M2 and M8 are identified by the Bayes Empirical Bayes (BEB) method (*P > 95%; **P > 99%). The Naive Empirical Bayes (NEB) method produced similar results. The *ω* values as indicators of positive selection are boldfaced. Residues are numbered according to AMSIN.

### Structural diversity of bacterial defensins

**Figure EV3:**
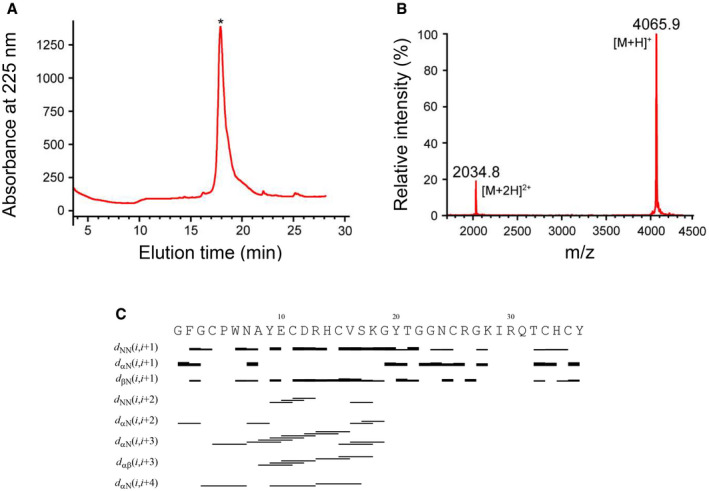
Oxidative refolding and identification of AMSIN Reversed‐phase high‐performance liquid chromatographic (RP‐HPLC) profile of oxidized AMSIN (marked by an asterisk).MALDI‐TOF MS. The two main peaks correspond to the singly and doubly protonated forms of AMSIN, respectively.Summary of nuclear Overhauser effect (NOE) data of AMSIN. The thickness of the bars indicates the intensity of the NOEs.

**Figure 2:**
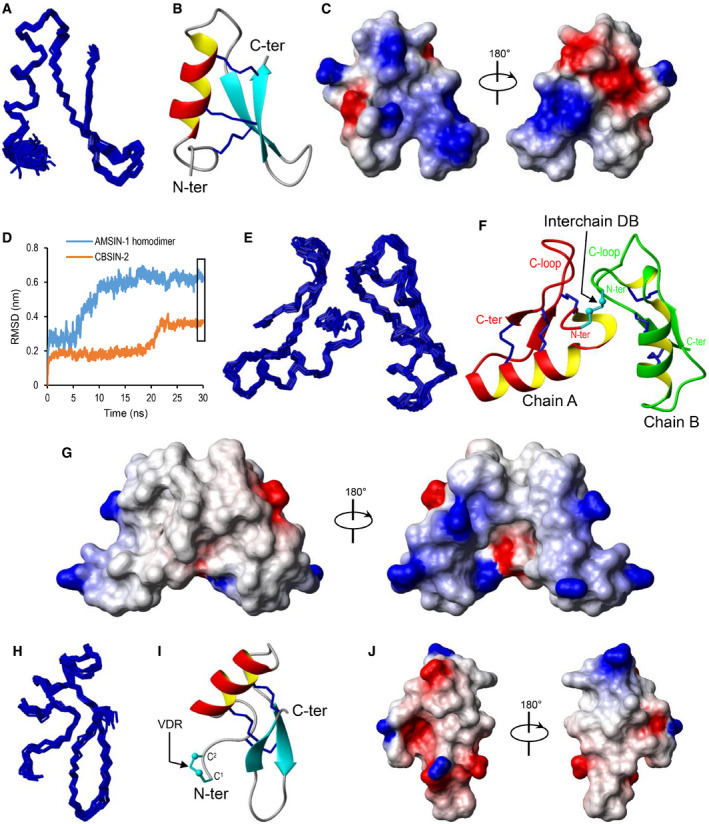
Representative three‐dimensional (3D) structures of bacterial defensins A–CThe NMR structure of AMSIN. A. Ensemble of the 20 lowest energy conformers superimposed over the backbone atoms of residues 3–36. B. A ribbon representation with disulfide connectivities shown in sticks. C. Electrostatic potential map. Red, blue, and gray represent negatively charged, positively charged, and electrostatically neutral zones, respectively.DBackbone‐RMSDs for AMSIN‐1 and CBSIN‐2, shown as a function of time (30‐ns simulations). The box marks the time for extracting the snapshots from the trajectories (49.9–50 ns) in (E) and (H). A total of 11 snapshots were taken from an MD simulation.E–JSimulated 3D structures of AMSIN‐1 and CBSIN‐2. Their presentations are in the same pattern with that of AMSIN (E‐G for AMSIN‐1 and H‐J for CBSIN‐2). The interchain disulfide bridge in AMSIN‐1 and the Cys1‐Cys2 vicinal disulfide ring (VDR) in CBSIN‐2 (shown as sticks) are denoted by arrows. The N‐ and C‐termini are labeled. Data information: All structure images are displayed by MOLMOL.

All the bacterial defensins contain six cysteines identical to those of eukaryotic AITDs, which can form three disulfide bridges. In some members, single or a pair of additional cysteines were evolved in their N‐termini (Fig 1; Appendix Fig S2). We explored these cysteine‐based structural variations through nuclear magnetic resonance (NMR), homology modeling, and molecular dynamics (MD) simulations. AMSIN is a defensin encoded by the genome of the oral‐associated species *Actinomyces* sp. oral taxon 171 str. F0337 and shares about 50–65% sequence identity to several eukaryotic defensins (Fig 1D). For the determination of its solution structure, we carried out the chemical synthesis and oxidative refolding (Fig EV3A and B). The structural statistics of AMSIN from the nuclear magnetic resonance (NMR) analysis are summarized in Table EV3 and the summary of its nuclear Overhauser effect (NOE) data in Fig EV3C. The NMR signals of all residues except Ile29, Arg30, and Gln31 were assigned. More than 11.7 constraints per residue were employed for the structure calculation. For the final 20 structures, no violations were found in distance (> 0.3 Å) and angle (> 5 degrees) restraints. The unassigned region (Ile29 to Gln31) is located in the C‐loop region connecting the two β‐strands. This is very similar to the results for micasin (Zhu *et al*, 2012). The NMR ensemble, ribbon model, and the molecular surface colored with charge distribution are shown in Fig 2A–C. AMSIN adopts a typical structure of the cysteine‐stabilized α‐helix and β‐sheet (CSαβ) superfamily (Zhu *et al*, 2005), in which the helical region spans residues 8–16 and the antiparallel β‐sheet is formed by residues 23–26 and 32–35 (Fig 2B). This structure is highly similar to those found in fungi and animals, particularly in their secondary structure elements (Appendix Fig S4).

AMSIN‐1 is a paralog of AMSIN and has the seventh cysteine in its N‐terminus (Fig 1D). Using homology modeling combined with MD simulations (Fig 2D), we were able to build a homodimer structure for AMSIN‐1, where the seventh cysteine is involved in an intermolecular disulfide bridge (Fig 2E–G). In this dimer structure, there exists a continuous hydrophobic surface formed by residues in the N‐terminus and the C‐loop (Fig 1D; Appendix Fig S5). Since some eukaryotic defensins function by forming a dimer to interact with the microbial target (Zhang *et al*, 2010; Song *et al*, 2011), it is likely that such form in AMSIN‐1 is of functional significance. At the same time, using this strategy, we built the structure of CBSIN‐2, a defensin from *Corynebacterium* sp. KPL1995, which contains two adjacent cysteines in its N‐terminus. These two cysteines form a rare structural element called vicinal disulfide bridge (Fig 2H–J), as found in some native proteins or oxidative folding intermediates (Carugo *et al*, 2003; Cemazar *et al*, 2003).

### Antibacterial activity and synergy of AMSIN with oral AMPs

**Figure 3:**
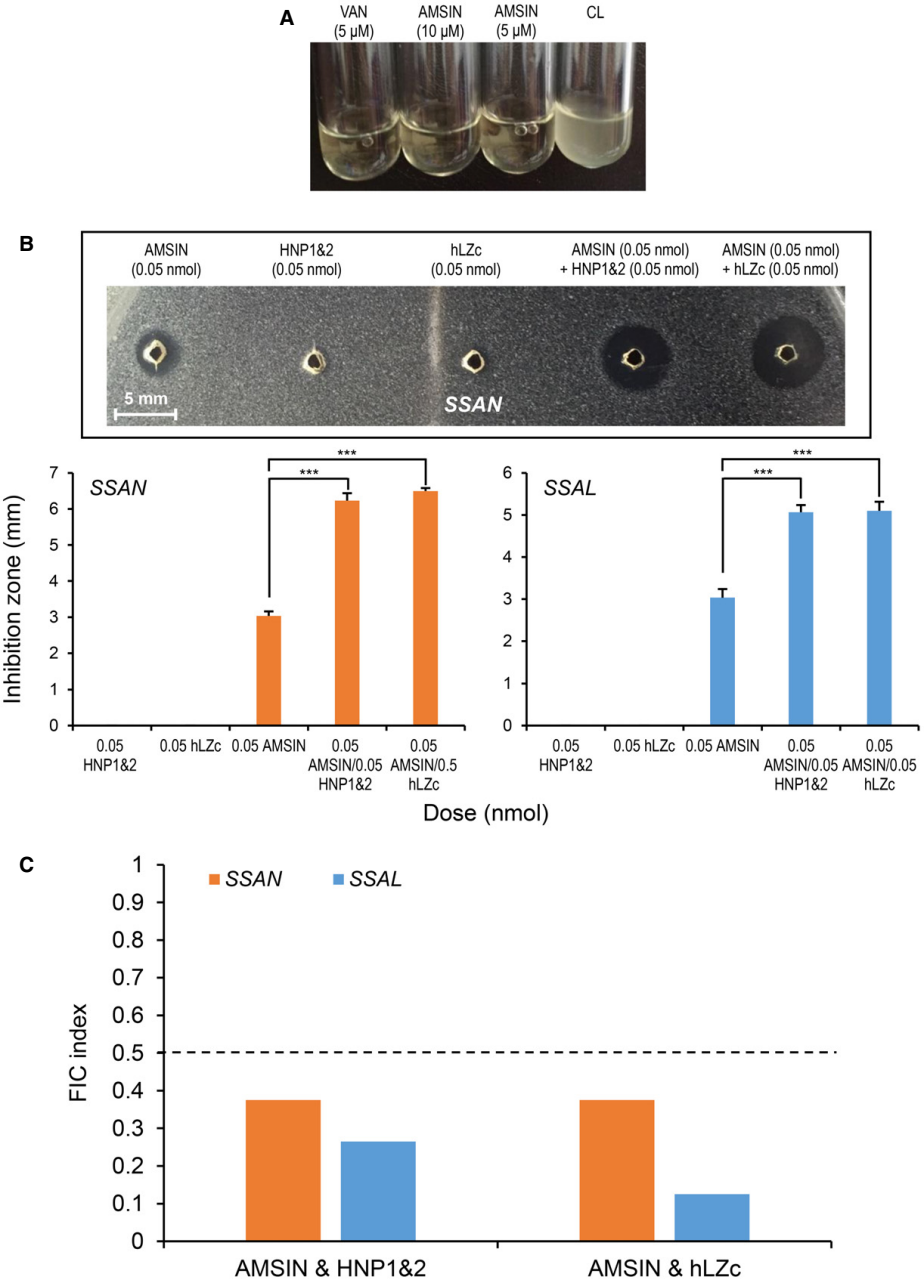
Antibacterial activity of AMSIN and its synergy with HNP1&2 or lysozyme C Lysis of *SSAN* by AMSIN. Vancomycin (abbreviated as VAN) was used as a control.Inhibition‐zone‐based assay for synergy between AMSIN and HNP1&2 or lysozyme C (abbreviated as hLZc) to two oral *Streptococcus* (*SSAN* and *SSAL*). Inset (upper panel, boxed), a representative example of the inhibition‐zone test showing synergy. Peptide doses used here were 0.05 nmol/well for HNP1&2 and hLZc at which no visible inhibition zones were observed for both of them. At the same dose, AMSIN produced a small inhibition zone. For synergetic observation, two peptides were applied to the same well according to the doses indicated. Mean ± SD of three biological replicates is displayed. *P* values were obtained by Student’s *t*‐test (***P < 0.001; exact *P* values are listed in Table EV7). Note: the nanomolar concentration of HNP1&2 was calculated from the average molecular weight of HNP1 and HNP2.The FIC index assay for synergy between AMSIN and HNP1&2 or hLZc to *SSAL* and *SSAN*. The dashed line represents the cutoff of the FIC index (0.5) for synergy. Source data are available online for this figure.

Next, we conducted a series of experiments to assess the antibacterial activity, the interacting target(s) and the therapeutic potential of AMSIN. It showed a lytic effect on bacteria (Fig 3A). Using the classical inhibition‐zone assay (Hultmark, 1998), we quantitatively evaluated its antibacterial potency on various bacteria, primarily including some oral *Streptococcus* strains and clinically relevant strains in human and animal infections (Table 2; Table EV4). The results are summarized as follows: (i) AMSIN exhibited excellent activity against gram‐positive bacteria with the strongest potency toward *SP* (strains D39, R6, ST556, and TIGR4), including a streptomycin‐resistant strain. The lethal concentrations (C*_L_*) determined were about 0.10 μM (Table 2); (ii) It was also highly potent to three oral *Streptococcus* bacteria (*S. mutans*, *S. salivarius*, and *S. sanguinis*; abbreviated as *SM*, *SSAL*, and *SSAN*, respectively) with a C*_L_* of 0.5–1.4 μM; (iii) AMSIN was highly active to the *Staphylococcus* bacteria, including a series of clinical isolates. For example, for methicillin‐resistant strains (i.e., *MRCNS* P1369, *MRSA* P1374, and *MRSA* P1386), the C*_L_* ranged from 0.6 to 1.0 μM and for penicillin‐resistant strains (e.g., *PRSA* P1383 and *PRSE* P1389), the C*_L_* ranged from 1.0 to 5.0 μM; (iv) Its antibacterial spectrum also includes *Enterococcus faecium* (Table 2), a major pathogen of enterococcal sepsis in the neonate (Nizet & Klein, 2011). Since in some AMPs (e.g., protegrin‐1 and HNP1), the disulfide bridges are not functionally essential (Varkey & Nagaraj, 2005; Dawson & Liu, 2010), we assessed this case in AMSIN. The result showed that its reduced peptide exhibited an antibacterial activity comparable to the cyclic version (Appendix Fig S6).

**Table 2:** Lethal concentrations (C*_L_*) of AMSIN against various bacteria.

Bacterial strains	C*_L_* (µM)
*Bacillus megaterium* CGMCC 1.0459	0.11
*Bacillus megaterium* CGMCC 1.0459	0.038^a^
*Bacillus megaterium* CGMCC 1.0459	0.045^b^
*Bacillus subtilis* CGMCC 1.2428	1.00
*Staphylococcus aureus* CGMCC 1.89	5.20
penicillin‐sensitive *Staphylococcus epidermidis* P1111	5.20
penicillin‐resistant *Staphylococcus epidermidis* P1389	1.14
penicillin‐resistant *Staphylococcus aureus* P1383	4.28
Methicillin‐resistant *Staphylococcus aureus* P1374	0.62
Methicillin‐resistant coagulase negative *Staphylococci* P1369	1.07
Methicillin‐resistant *Staphylococcus aureus* P1386	0.74
*Staphylococcus aureus* J685	10.40
*Staphylococcus aureus* J698	5.20
*Staphylococcus aureus* J700	5.20
*Staphylococcus aureus* J706	5.20
*Staphylococcus aureus* J708	1.14
*Staphylococcus aureus* J710	2.60
*Streptococcus pneumoniae* D39	0.09
*Streptococcus pneumoniae* R6	0.14
*Streptococcus pneumoniae* ST556	0.14
*Streptococcus pneumoniae* ST556 (StrR)	< 0.14
*Streptococcus pneumoniae* TIGR4	0.14
*Streptococcus mutans* CGMCC 1.2499 (ATCC 25175)	1.01
*Streptococcus mutans* CGMCC 1.2499 (ATCC 25175)	0.38^a^
*Streptococcus mutans* CGMCC 1.2499 (ATCC 25175)	0.56^b^
*Streptococcus salivarius* CGMCC 1.2498 (ATCC 7073)	0.49
*Streptococcus sanguinis* CGMCC 1.2497 (ATCC 49295)	1.41
*Streptomyces griseus* NBRC 13350	2.89
*Streptomyces scabiei* CGMCC 4.1765 (ATCC 49173)	2.01
*Lysinibacillus fusiformis*	0.27
*Enterococcus faecalis* V583 (ATCC 700802)	4.65

StrR, streptomycin‐resistant.

^a^ In combination with HNP1&2.

^b^ In combination with hLZc.

Because of the origin of AMSIN from an oral bacterium, we assessed its potential synergy with human oral‐derived antibacterial factors (the α‐defensins HNP1&2 and lysozyme C) isolated from human saliva (Appendix Fig S7). HNP1&2 is a mixture of HNP1 and HNP2 co‐eluted in our purification process (Appendix Fig S7) (Note: HNP1 has an extra N‐terminal Ala relative to HNP2, but this does not create an effect on their antibacterial activity) (Ericksen *et al*, 2005). When AMSIN and a sub‐inhibitory dose of HNP1&2 or lysozyme C were jointly applied to the same well, the inhibition zone was significantly increased, compared with AMSIN alone (Fig 3B; Appendix Fig S8), and accordingly its C*_L_* values for *Bacillus megaterium* (abbreviated as *BM*) and *SM* descended (Table 2). Using the liquid fractional inhibitory concentration (FIC) assay, we confirmed that the FIC indexes for *SSAL* and *SSAN* (two representatives of oral bacteria) all were < 0.5 (Fig 3C). Both assays, thus, indicate synergy between AMSIN and human oral antibacterial factors. Using bio‐affinity electrospray ionization mass spectrometry (ESI‐MS), a technique for the evaluation of solution‐phase non‐covalent interactions (McCullough & Gaskell, 2009), we found no complex formation between AMSIN and HNP1&2 or hLZc (Appendix Fig S9), suggesting that their antibacterial synergy is independent on their physical interactions.

### AMSIN is an inhibitor of bacterial cell‐wall biosynthesis

**Figure 4:**
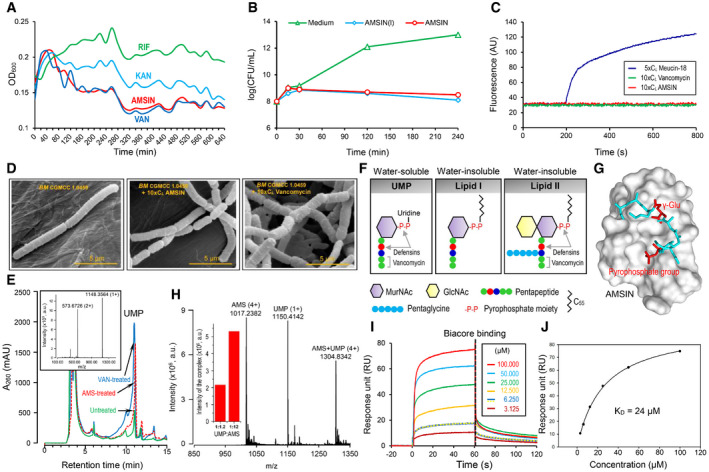
Evidence for inhibiting the cell‐wall synthesis of bacteria by AMSIN Growth kinetic measurements of *SM* exposed to AMSIN or various antibiotics with known cellular targets. The peptide or the compounds used were 4 × C*_L_*. VAN, vancomycin; RIF, rifampicin; KAN, kanamycin. OD_600_ were calculated as measured values subtracting the background value from broth medium (OD_600_ = 0.356).Killing kinetic measurements of *SM* exposed to AMSIN or its linear version. The peptide dose used was 4 × C*_L_*.Effect of AMSIN on the *SM* membrane at a 10 × C*_L_* dose. Propidium iodide, a dead cell stain that emits red fluorescence when bound to DNA, was used to monitor membrane damage by the compounds as a function of time. Vancomycin and Meucin‐18 were used as negative and positive controls, respectively.Scanning electron microscopic observation of AMSIN‐ and vancomycin‐induced *BM* deformation. The dose used was 10 × C*_L_*.Cytoplasmic accumulation of the soluble cell‐wall precursor UMP in vancomycin‐treated (blue line), AMSIN‐treated (red dashed line) or not treated (green line) culture of *MRSA* P1374. Inset: UMP identified by UPLC‐HRMS with singly and doubly protonated *m/z* observed.Water‐soluble and water‐insoluble cell‐wall precursors (UMP, Lipid I and Lipid II). In the pentapeptide (L‐Ala‐γ‐D‐Glu‐L‐Lys‐D‐Ala‐D‐Ala), Ala, Glu, and Lys residues are denoted by a dot in green, red, and blue, respectively. The γ‐D‐glutamic acid and the pyrophosphate group pointed by two arrows are the target sites of defensins in UMP or Lipid II, and the C‐terminal D‐Ala‐D‐Ala residues marked by a single square bracket are the target sites of vancomycin in UMP or Lipid II.The predicted complex structure showing that the γ‐ glutamate and pyrophosphate group (shown as a stick model) are involved in direct interactions of UMP/Lipid II with AMSIN (presented as a molecular surface model).ESI‐MS detecting the non‐covalent complex between AMSIN and UMP. The quadruply protonated complex (*m/z* 1304.8) is observed. Inset: comparison of intensities of the complex between two ratios of UMP:AMSIN (For the full image, see Appendix Fig S11). Here, AMSIN is abbreviated as AMS due to space limitation.Sensorgrams of UMP binding to AMSIN. The peptide was covalently immobilized to CM5 via its amine groups, and UMP with different concentrations flowed by. The 6.250 μM analyte concentration analyzed in duplicate is shown by a yellow dotted line.The dose–response unit curve of UMP binding to AMSIN, which is fitted by the BIAevaluation v2.0.1. software using 1:1 Langmuir binding model. Source data are available online for this figure.

**Figure EV4:**
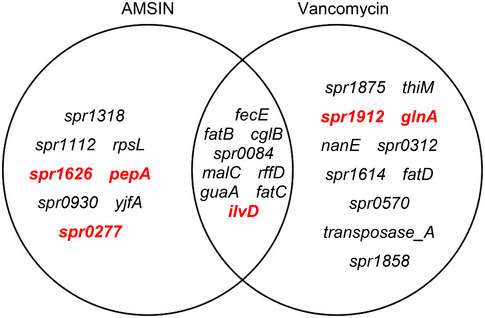
Venn diagram for comparison of the AMSIN and vancomycin stimulon of *S. pneumoniae* R6 The upregulated and downregulated genes stimulated by a sub‐lethal concentration of antibacterial agents are shown in red and black, respectively.

To study the mechanism of action by which AMSIN kills bacteria, we first measured its impact on the growth kinetics of the gram‐positive *SM* and compared it with three antibiotics with completely different bactericidal mechanisms. AMSIN exhibited a kinetic behavior highly similar to vancomycin, a bacterial cell‐wall synthesis inhibitor but different from rifampicin (a bacterial DNA‐dependent RNA polymerase inhibitor) and kanamycin (a protein synthesis inhibitor) (Fig 4A). *In vitro* killing kinetics indicated that AMSIN‐treated *SM* slightly grew within 30 min, in accordance with the growth kinetics curve (Fig 4A and B), and then the number of colony‐forming units (CFU) decreased remarkably relative to the control (Fig 4B). In comparison with the cyclic AMSIN, the linear one displayed a slightly quicker killing rate after 120 min (Fig 4B). Consistently to vancomycin, AMSIN did not impair the integrity of bacterial membrane (Fig 4C), but the treatment of *BM* cells with AMSIN or vancomycin caused a deformation of the cell shape presumably due to the cell‐wall synthesis inhibited (Fig 4D). We obtained further support for the cell‐wall‐interfering activity using RNA sequencing to compare the short‐term alterations in transcription induced by a sub‐lethal concentration of AMSIN or vancomycin with *SP* R6. We found that their transcriptional profile partly overlapped (Fig EV4; Dataset EV5).

**Figure EV5:**
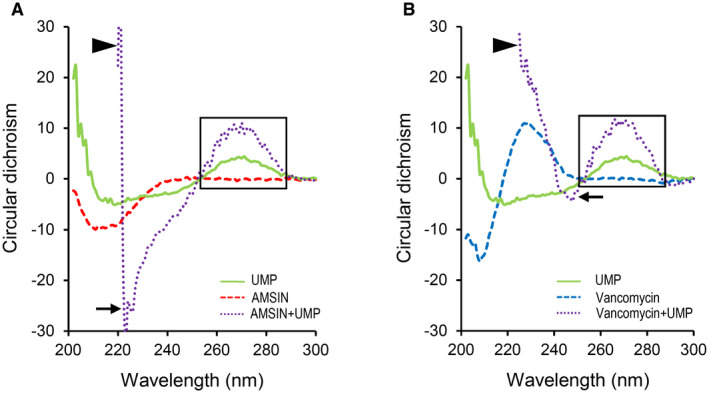
CD spectra of AMSIN and vancomycin in the absence or presence of UMP A, BIn these two mixtures, emerging maximum and minimum CD absorbance peaks are marked by triangle and arrow, respectively, and the positive band around 270 nm common to UMP is boxed. (A) AMSIN. (B) Vancomycin.

Using RP‐HPLC and mass spectrometry techniques, we analyzed the intracellular cell‐wall precursor pool of *MRSA* P1374 treated by AMSIN. The soluble precursor UDP‐MurNAc‐pentapeptide (abbreviated as UMP) was accumulated in AMSIN‐ or vancomycin‐treated cells compared with the untreated cells (Fig 4E), suggesting that the peptide may work in a similar manner to the inhibitors of cell‐wall synthesis. Because UMP contains the pyrophosphate group and the γ‐Glu for the interaction of Lipid II with the fungal defensin—plectasin and the D‐Ala‐D‐Ala motif for vancomycin (Hernout *et al*, 2007; Schneider *et al*, 2010) (Fig 4F; Appendix Fig S10), we inferred that UMP might be used as an alternative to study the interaction of Lipid II with AMSIN. A computational model predicting the interaction is shown in Fig 4G. Because circular dichroism (CD) has been proved useful in the identification of the vancomycin–UMP complex by recognizing differential absorption signatures (Perkins, 1969; Best *et al*, 1970), we first compared the absorption signatures of AMSIN in the absence or presence of UMP by CD. A distinct adsorption minimum at 223–226 nm and a maximum at 221 nm were observed exclusively in the AMSIN–UMP mixture (Fig EV5A) and similarly, two adsorption signatures specific for the vancomycin–UMP mixture were also observed (Fig EV5B), indicating that in these two cases, a complex was formed. Using ESI‐MS, we detected a quadruply protonated complex between AMSIN and UMP at *m/z* 1,304.83 and found that increasing the ratio of AMSIN to UMP induced more complex formation (Fig 4H). In the same manner, we verified the complex formation between vancomycin and UMP but not between drosomycin (a specific antifungal defensin) (Fehlbaum *et al*, 1994) and UMP (Appendix Fig S11). These data confirmed the specificity of the interaction between AMSIN and UMP. Using surface plasmon resonance (SPR) experiments with BIAcore T100 (Fig 4I), we determined the binding affinity of AMSIN to UMP (*K*_D_ = 24 μM) (Fig 4J).

Targeting Lipid II by AMSIN may provide an explanation for its synergy with the oral antibacterial factors observed here: (i) Partial hydrolysis of the β‐1,4 glycosidic linkages of the cell‐wall peptidoglycan by a small amount of lysozyme (Ragland & Criss, 2017) could prompt easier access of AMSIN to the membrane‐anchored Lipid II. (ii) Binding of HNP1&2 to Lipid II might induce a conformation that more facilitates AMSIN’s binding. This is in line with the findings that HNP1 and plectasin bind to two separate regions on Lipid II (Oppedijk *et al*, 2016) (Appendix Fig S12).

### The therapeutic potential of AMSIN

**Figure 5:**
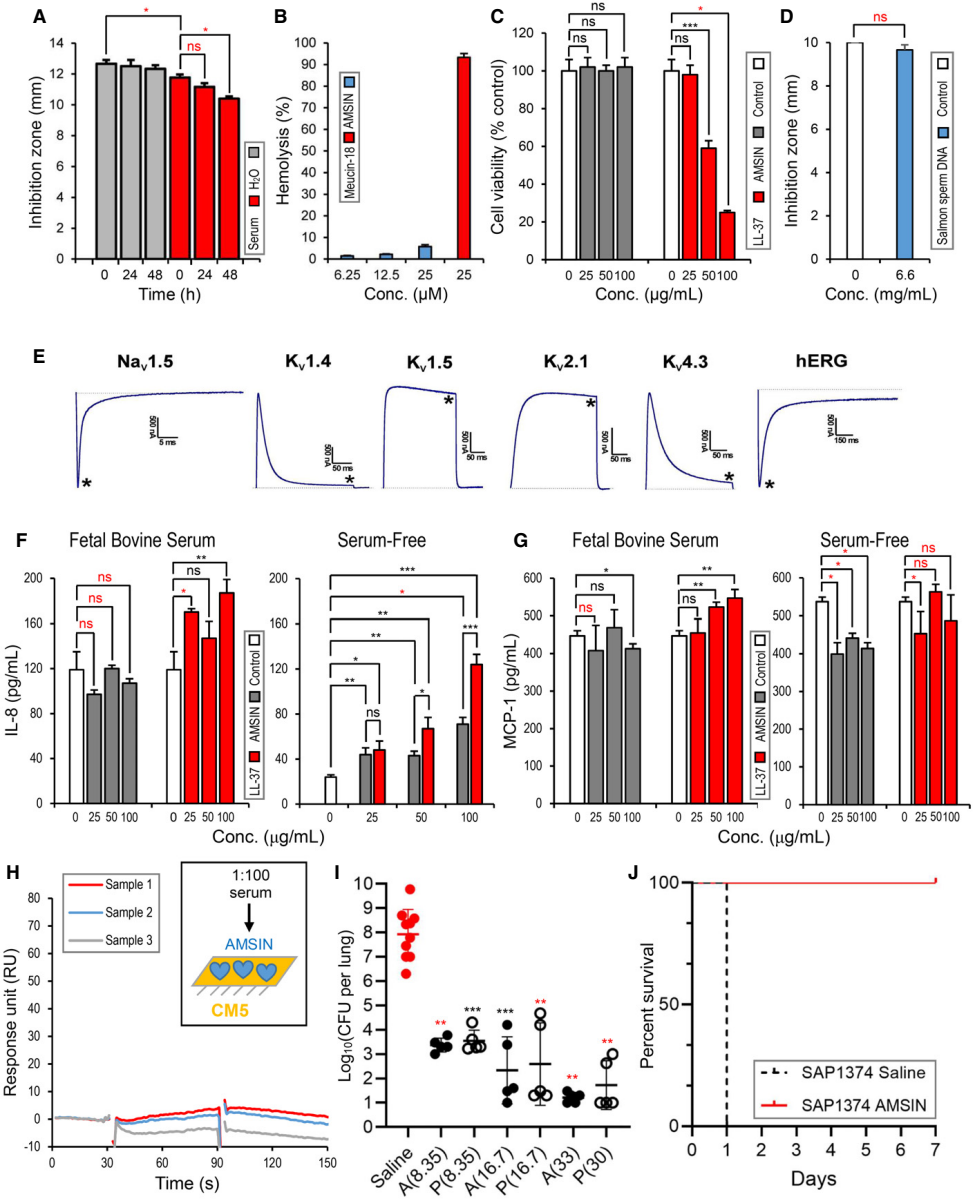
The therapeutic potential of AMSIN An inhibition‐zone‐based assay for evaluating the stability of AMSIN in H_2_O or normal mouse serum. The bacterium used was *BM* and the peptide dose was 0.2 nmol/well. Mean ± SD of three biological replicates is displayed. *P* values were obtained by Mann–Whitney *U*‐test (*P < 0.05, ns: no significance; exact *P* values are listed in Table EV7).Hemolysis by AMSIN. Meucin‐18 was used as a positive control. Mean ± SD of three technical replicates is displayed.Comparison of cytotoxic effects of AMSIN and LL‐37 on HL‐60 cells. Mean ± SD of three biological replicates is displayed. *P* values were obtained by Student’s *t*‐test or Mann–Whitney *U*‐test (the latter indicated by a red asterisk; *P < 0.05, ***P < 0.001, ns: no significance; exact *P* values are listed in Table EV7).An inhibition‐zone assay showing the inability of AMSIN to bind DNA. The bacterium used was *MRSA* P1374 and the peptide dose was 1.0 nmol/well. Mean ± SD of three biological replicates is displayed. *P* values were obtained by Mann–Whitney *U*‐test (ns: no significance; exact *P* values are listed in Table EV7).The effect of AMSIN on human ion channels expressed in the CNS and heart. The asterisks mark steady‐state current traces after administering 100 μM AMSIN.Peptide‐induced IL‐8 release. A549 cells were treated with AMSIN or LL‐37 for 24 h. IL‐8 levels in culture supernatants from the FBS‐containing medium (*left*) or the serum‐free medium (*right*) were measured with an ELISA, as described in Materials and Methods. Mean ± SD of three biological replicates is displayed. *P* values were obtained by Student’s *t*‐test or Mann–Whitney *U*‐test (the latter indicated by a red asterisk or ns; *P < 0.05, **P < 0.01, ***P < 0.001, ns: no significance; exact *P* values are listed in Table EV7).Peptide‐induced MCP‐1 release. HL‐60 cells were treated with AMSIN or LL‐37 for 24 h. MCP‐1 levels in culture supernatants from the FBS‐containing medium (*left*) or the serum‐free medium (*right*) were measured with an ELISA. Mean ± SD of three biological replicates is displayed. *P* values were obtained by Student’s *t*‐test or Mann–Whitney *U*‐test (the latter indicated by a red asterisk or ns; *P < 0.05, **P < 0.01, ns: no significance; exact *P* values are listed in Table EV7).Surface plasmon resonance (SPR)‐based assay detecting anti‐AMSIN antibodies in mouse serum using Biacore T100. Sera were taken from three AMSIN‐treated mice (Samples 1–3) and one control mouse only injected with 0.9% NaCl. The sensorgrams present data of subtracting the background SPR signals from the control. Note: the abnormal peaks at 30 and 90 s yielded by the equipment due to sample injections were artificially removed. Insect, schematic diagram of SPR experiment in which 1:100 diluted serum was used as analyte to flow over the AMSIN‐immobilized CM5 chip.Treatment of pneumococcal pneumonia. Five mice per sampling point were inoculated with *SP* D39 through the nasopharynx and 24 h later the animals received AMSIN or penicillin (as a positive control) at different doses (AMSIN, at the doses of 8.35, 16.7, and 33 mg per kg; penicillin at 8.35, 16.7, and 30 mg per kg) via intramuscular injection (i.m.). One day after treatment, the animals were necropsied and their lungs were removed and homogenized for the determination of the level of *SP*, as described in the Materials and Methods section. Each point represents the determination from a single animal and the line shows the mean log value. Mean ± SD of five to ten biological replicates is displayed. *P* values were obtained by Student’s *t*‐test or Mann–Whitney *U*‐test (the latter indicated by red asterisks; **P < 0.01, ***P < 0.001 denotes significant reduction in CFU compared with the saline group; exact *P* values are listed in Table EV7).Survival after peritoneal infection with *MRSA* P1374. The survival fraction of the vehicle‐treated control group was zero out of 11 mice and the treatment group contained six mice and all survived. Source data are available online for this figure.

Because targeting the cell‐wall synthesis may potentially reduce the risk of non‐specific toxicity and lower the potential of developing resistance compared with those non‐specifically membrane‐targeting human AMPs (Peschel, 2002; Ling *et al*, 2015; Witzke *et al*, 2016), we evaluated the therapeutic potential of AMSIN. In the assay time, AMSIN remained its full antibacterial activity after 48‐h incubation in H_2_O at 37°C and 24 h in fresh normal mouse serum without dilution (Fig 5A). Compared with in H_2_O, the activity of AMSIN in undiluted mouse and human serums only showed a slight decrease in the 48‐h study period (Fig 5A and Appendix Fig S13). These data indicated the stability of AMSIN in both water and serums. In addition, AMSIN exhibited a considerable low hemolysis (Fig 5B) and did not affect the viability of two human‐derived cell lines (human neutrophil‐like cell HL‐60 and human embryonic kidney HEK 293) at concentrations far beyond its lethal concentrations for bacterial killing (Fig 5C; Appendix Fig S14; Table 2). In contrast to AMSIN, the human AMP–LL‐37 (Scott *et al*, 2002) significantly reduced the viability of HL‐60 cells in a concentration‐dependent manner (Fig 5C). In the presence of plenty of DNA molecules, AMSIN retained its full antibacterial activity (Fig 5D), indicating its inability to bind DNA and the safety to human genetic stability. Although having the same fold as the scorpion toxins affecting mammalian and human ion channels, AMSIN showed no any effect on a panel of voltage‐gated ion channels expressed in the human central nervous system (CNS) and the heart at a rather high dose (100 μM) (Fig 5E). In these assayed channels, Na_v_1.5, K_v_1.5, K_v_4.3, and hERG (i.e., human ether‐à‐go‐go‐related gene potassium channel, also known as K_v_11.1) are commonly involved in heart action potential (Wulff *et al*, 2009; Jiang *et al*, 2020). Our study, thus, highlights the safety of AMSIN to human heart.

To evaluate the potential immunomodulatory effect of AMSIN, we assayed its ability in inducing cytokine release by two human‐derived cell lines (human type II alveolar epithelial cell line A549 and HL‐60) with ELISA. LL‐37, the human AMP with immunomodulatory properties (Scott *et al*, 2002), was used as a positive control. Interleukin‐8 (IL‐8) and monocyte chemotactic protein‐1 (MCP‐1), the respective prototypes of α‐chemokines (the CXC family) (Baggiolini & Clark‐Lewis, 1992) and β‐chemokines (the CC family) (Steube *et al*, 1999; Deshmane *et al*, 2009), were chosen in this study given that their elevated expression is often associated with various human diseases (Deshmane *et al*, 2009; Alfaro *et al*, 2017; Ha *et al*, 2017). The results showed that in an FBS‐containing medium, AMSIN did not significantly enhance the release of IL‐8 by A549 and the release of MCP‐1 by HL‐60 at a concentration range (25–100 μg/ml) but LL‐37 did (Fig 5F and G). In the medium without the serum, AMSIN exerted some effect on IL‐8 release by A549, but this effect was weaker than that of LL‐37 (Fig 5F). In this medium, AMSIN and LL‐37 both reduced the release of MCP‐1 by HL‐60 in a concentration‐independent manner (Fig 5G). Due to the cytotoxicity of LL‐37 on HL‐60 leading to the decrease in the number of living cells (Fig 5C), its impact on MCP‐1 release should be considered as just a reference (Fig 5G). Taken together, our data show that AMSIN has a rather weak immunomodulatory effect on human cells.

SPR experiments detected no obvious anti‐drug antibody in the AMSIN‐treated mouse serum, as identified by very weak response units (RUs) when the serum flowed through the AMSIN‐immobilized chip surface (Fig 5H), suggesting that as a peptide‐based drug AMSIN was less immunogenic than other biologic therapeutics (Audie & Boyd, 2010). We also predicted the immunogenicity of AMSIN with VaxiJen and its allergenicity by AllerTOP v. 2.0, and compared them with those of some currently developed peptide drugs (Table EV5) (Yang *et al*, 2017; Koo & Seo, 2019). Overall, AMSIN has a predicted immunogenicity (score 0.76) smaller than the peptides (scores: 0.84–2.19) and is a probable non‐allergen (Table EV5).

The *in vivo* anti‐infective potency of AMSIN was evaluated in two mouse models of systemic infections. In the mouse pneumonia model with the *SP* D39 strain, AMSIN displayed high efficacy on the infected mice, causing a 4.6–6.7 log10 reduction of CFU in lungs when different doses of peptides were used, which was comparable to the efficacy of penicillin (Fig 5I). In the peritonitis model, mice were intraperitoneally (i.p.) injected with *MRSA* P1374 at a bacterial dose (1 × 10^8^ CFU per mouse) that led to 100% death (Fig 5J). AMSIN was introduced intravenously (i.v.) 1 h post‐infection at 20 mg per kg (prim.vic. No2) and then the treatment continued for 5 days at 10 mg per kg once daily. Six mice treated with AMSIN survived the 7‐day study period (Fig 5J) and their survival continued for more than the 30‐day observation period.

## Discussion

Because over‐mining of antibiotics from cultured soil microbes with the traditional method ended the golden era of antibiotic discovery in the 1960s (Waksman *et al*, 2010; Lewis, 2012), exploiting the resources of uncultured bacteria has been proved successful, as exemplified by the discovery of teixobactin (Ling *et al*, 2015). Compared with this strategy, the mining of new AMPs from the sequenced microbial genomes represents an emerging direction in the post‐genomic era. At the beginning of this work 7 years ago, as recorded by the PDB deposition date for AMSIN on 2013/10/17 in the GenBank database (https://www.ncbi.nlm.nih.gov/Structure/pdb/2RU0) and its chemical shift data at BioMagResBank on 2013‐10‐16 (https://bmrb.io/data_library/summary/index.php?bmrbId=11537), bacterial defensins were an untapped resource. In this study, we report this resource and demonstrate that the CSαβ‐type defensins are one class of AMPs commonly present in both prokaryotic and eukaryotic organisms with a 2‐billion‐year history (Futuyma & Kirkpatrick, 2017). Their evolutionary success hints at their role in providing fitness for these organisms through the history of cellular organisms. It is remarkable that in more than 100,000 bacterial genomes screened, which covered nearly 40 microbial subgroups (Fig EV1), only species from actinomycetes and myxobacteria express defensins. Such a patch distribution in bacteria suggests that the evolution of defensins might involve lateral (horizontal) gene transfer between prokaryotes and eukaryotes (Soucy *et al*, 2015; Sieber *et al*, 2017). Since these two subgroups of bacteria also produce most natural antibiotics for antagonism against their competitors (Waksman *et al*, 2010; Wright, 2012; Le *et al*, 2014; Landwehr *et al*, 2016; van der Meij *et al*, 2017; Wink, 2020), we infer that their defensins may exert a similar role in ecological competition to provide a substantial benefit to the producers (e.g., for nutritional competition or predation) (Xiao *et al*, 2011). In support, positive selection has driven their accelerated protein evolution (Fig 1E; Table 1). Conversely, eukaryotic defensins often have other biological functions beyond the antibiotic activity. As do their mammalian counterparts, insect CSαβ‐type defensins might function as stress proteins with chemotactic properties (Boulanger *et al*, 2006). A similar opinion was also proposed for fungal defensins (Contreras *et al*, 2019). The multifunctionality could account for the origin of non‐specific toxicity in these AMPs, in which some basic physiological functions might restrict adaptive changes in these AITDs from fungi and animals (Tables EV1 and EV2).

Therefore, defensins from oral or rumen actinomycetes are more likely leads than multifunctional eukaryotic defensins in drug design given that their antibacterial function has been optimized by complex microflora‐driven natural selection, as mentioned previously. In this work, we have provided experimental evidence confirming that actinomycetes‐derived defensins are indeed a class of promising systemic drugs for treating infections of *SP* and drug‐resistant *S. aureus* through inhibiting their bacterial cell‐wall synthesis. As the representative of actinomycetes‐derived defensins, AMSIN lacked obvious cytotoxicity on mammalian and human cells and displayed weak immunomodulatory effect with relatively high serum stability (Fig 5; Appendix Fig S13). Of all these properties relevant to therapeutic efficacy, AMSIN is superior to eukaryotic AMPs (e.g., human LL‐37 and defensins). The latter often exhibits potent mammalian cytotoxicity with stronger immunomodulatory activity and serum instability and inactivation (Panyutich *et al*, 1995; Johansson *et al*, 1998; Scott *et al*, 2002; Hara *et al*, 2008; Zhu & Gao, 2017; Kudryashova *et al*, 2021). From an evolutionary viewpoint, natural selection on mammalian LL‐37 has been proposed to be driven by their endogenous receptors mediating the immunomodulatory functions rather than by exogenous pathogens (Zhu & Gao, 2017). This may explain the difference in the immunomodulatory activity between AMSIN and LL‐37 and further highlight an inherent advantage of these bacterial defensins over human AMPs as anti‐infective drugs. Encapsulation of AMSIN into liposomes (Pan *et al*, 2012) or PEGylation (Veronese & Mero, 2008) may further improve its therapeutic efficacy through eliminating its minor unwanted properties. From the viewpoint of the drug industry, these bacterial defensins can be considered as completely novel antibacterial scaffolds compared with conventional antibiotics from bacteria, though they are extensively expressed in eukaryotes. Further evaluation of other homologs reported here may have the potential to accelerate their development into practical applications to control human systemic infections.

Of the three AMSIN‐sensitive oral bacteria tested here (Table 2), *SM* is a primary colonizer of teeth and also a major cariogenic pathogen causing human oral disease (dental caries), especially in the case of disease onset (Krzyściak *et al*, 2014; Lemos *et al*, 2019). Based on its potent antibacterial activity and synergy with oral AMPs, we can infer that AMSIN may play certain roles directly related to oral health by inhibiting the overgrowth of *SM* on the tooth surface in the normal physiological condition, and thus likely has a potential as a drug for human dental caries. AMSIN might also be a valuable lead for developing novel drugs for treating infective endocarditis elicited by the opportunistic oral pathogen *SSAN* (Turner *et al*, 2009).

Several classes of peptide antibiotics, such as bacterially produced vancomycin, lantibiotics, and teixobactin as well as eukaryotically derived defensins, have evolved the ability to specifically target the peptidoglycan precursor Lipid II (Oppedijk *et al*, 2016). Differing from the membrane‐disruptive mechanism adopted by most AMPs, this new mode of action will result in a reduced toxicity stemmed from general membrane distortion and a significantly delayed onset of resistance, as mentioned previously. For this class of studies, most Lipid II used is an enzymatically synthesized product, which requires a complicated process with many enzymatic steps and various substances (Huang *et al*, 2014). Recently, a general approach to obtain large quantities of Lipid II has been reported (Qiao *et al*, 2017). However, this method requires using a large amount of toxic chemicals (e.g., chloroform and methanol). Compared with Lipid II and some bacterial cell‐wall‐mimicking peptides (e.g., N,N′‐diacetyl‐L‐Lys‐D‐Ala‐D‐Ala, KAA) (Hernout *et al*, 2007), UMP displays obvious advantages. Firstly, compared with water‐insoluble Lipid II, water‐soluble UMP is highly compatible with current most commonly used interaction assay methods, such as ESI‐MS, surface plasmon resonance (SPR), isothermal titration calorimetry (ITC); secondly, UMP contains more elements than KAA for direct binding to study more kinds of antibiotics; thirdly, ease of preparation from cultured bacteria without using any toxic substances. These advantages will promote the wide application of UMP in the discovery and improvement of Lipid II‐targeted antibiotics via combination with the protein interaction techniques.

In summary, our work indicates that the microbial genome sequencing projects are an emerging resource not only for mining new operons coding for secondary metabolites (Lewis, 2017), but also for new genes encoding AMPs for systemic therapy of infectious diseases. Very recently, an actinomycete (*A. ruminicola*) isolated from sheep feces was reported to express an AMSIN homolog named actifensin that showed antibacterial activity (Sugrue *et al*, 2020). It is thereby evident that ongoing microbial genome sequencing actinomycetes with different origins (e.g., human and mammalian oral cavity, rumen, and feces) will bring more drug discovery innovations with the strategy developed here. Finally, given that humans live in a bacterial world (McFall‐Ngai *et al*, 2013), the discovery of defensins in human oral bacteria provides a new clue for further investigation of the relationships between the oral bacteria and humans (Verma *et al*, 2018) in the context of these newfound peptide antibiotics.

## Materials and Methods

### Animals

Animal experiments were performed according to the procedures approved by the Ethics Committee of the Institute of Zoology, Chinese Academy of Sciences. Institute of Cancer Research (ICR) mice (*Mus musculus*) were purchased from SPF (Beijing) Biotechnology Co., Ltd., Beijing, China. Animals were housed in a temperature‐controlled room (22–25°C) with water and food available ad libitum, and on a 12‐h light/dark cycle.

### Gene discovery

A TBLASTN‐based gene discovery strategy for identifying cysteine‐stabilized structural motif‐containing polypeptides has been described previously (Zhu, 2008; Zhu *et al*, 2012; Zhao *et al*, 2018). To explore potential defensin‐like peptides in bacteria, we downloaded all eukaryotic AMP sequences deposited in the APD3 database (https://aps.unmc.edu/) and then performed cluster analysis by the ClustalX program (http://www.clustal.org/). The clusters whose members were annotated as “defensin” were selected. To reduce the database search workload, we used CD‐HIT to remove redundant sequences with a cutoff of 0.5 (http://weizhongli‐lab.org/cd‐hit/). The collected sequences included *cis*‐ and *trans*‐classes (Shafee *et al*, 2016; Zhou *et al*, 2019). Using these sequences as queries, we conducted an iterative TBLASTN search of the microbial genomes database (https://www.ncbi.nlm.nih.gov/genome/microbes/) (began on June 15, 2011 and updated by February 28, 2019) which contained 39 microbial subgroups with genome assembly at different levels (Chromosome; Contig; Scaffold and Complete) (Fig EV1). GView, an interactive microbial genome visualization tool, was used to map defensin genes to their genomes (Petkau *et al*, 2010). The evolutionary tree of bacterial defensins was constructed based on their protein sequence alignment using the neighbor‐joining (NJ) method based on *p*‐distance model of amino acid substitutions with MEGA7 (Kumar *et al*, 2016).

### DNA sequencing the AMSIN cluster from *Actinomyces*

PCR was used to amplify the AMSIN cluster from the genome of *Actinomyces* sp. oral taxon 171 str. F0337 by two specific primers (FP: 5′‐TCCTCACCCCACACAGAACT‐3′; RP: 5′‐ACAGGCCCCGTGTTACGTCT‐3′). The resultant PCR product was ligated into a T‐vector for DNA sequencing (Tsingke Biological Technology, Beijing, China).

### Positive selection analysis

Detecting positive selection in AITDs from different origins (actinomycetes, fungi, and animals) was performed using the maximum likelihood‐based codon substitution models implemented in the CODEML program of the PAML software package (Yang, 2006). These models account for variable non‐synonymous/synonymous rate ratios (ω) among protein sites and they test positive selection using a likelihood ratio test (LRT) to compute the value twice the log likelihood difference (2Δ*l*) by comparing a null model that does not allow ω > 1 with an alternative model that does (Yang, 2006). Two pairs of models (M1/M2 and M7/M8) were chosen to test positive selection, in which M1 (nearly neutral model) is the null model of the positive selection model M2, and M7 (β‐distribution model) that does not allow for positive selection is the null model of M8 that is a β and ω model that allows for positive selection. The degree of freedom (df) for these two comparisons is two. A posterior probability for each positively selected site (PSS), if any, was then computed using the empirical Bayes method (Yang, 2006). Evolutionary trees constructed for maximum‐likelihood statistical analyses are provided in Appendix Fig S15.

### Creation of the defensin sequence logo

Bacterial defensin sequences were first aligned by ClustalX and then input into the Weblogo server (http://weblogo.berkeley.edu/logo.cgi) for creating the sequence logo using default parameters.

### Prediction of immunogenicity and allergenicity

The immunogenicity (protective antigens) of peptides was predicted by VaxiJen (http://www.ddg‐pharmfac.net/vaxijen/VaxiJen/VaxiJen.html) and the allergenicity (allergens) by AllerTOP v. 2.0 (http://www.ddg‐pharmfac.net/AllerTOP/).

### Chemical synthesis of peptides

LL‐37 and linear AMSIN were chemically synthesized in ChinaPeptides Co., Ltd. (Shanghai, China) with purity > 95%. For oxidative refolding of AMSIN, the synthetic peptide sample was dissolved in water in a concentration of 1 mg/ml and then dimethyl sulfoxide (DMSO) was added to a final concentration of 10% (v/v). The mixture was incubated at room temperature for 30 min and then 0.8 ml of PBS buffer (140 mM NaCl, 2.7 mM KCl, 10 mM Na_2_HPO_4_, and 1.8 mM KH_2_PO_4_, pH 7.5) was added. After incubation overnight at 25°C, the cyclic product was purified by RP‐HPLC and collected peaks were lyophilized by Thermo Scientific SAVANT SPD1010 SpeedVac Concentrator (USA) and analyzed by matrix‐assisted laser desorption/ionization time‐of‐flight mass spectrometry (MALDI‐TOF).

### NMR structural analysis

The purified AMSIN (2.0 mg) was dissolved in 300 µl H_2_O containing 10% D_2_O for the NMR lock, and the sample pH was adjusted to 3.0 with direct reading of a pH meter. After a series of NMR measurements, the sample was lyophilized and dissolved in D_2_O at pH 3.0 for another set of NMR experiments. Slowly exchanging amide protons suggesting that forming hydrogen bonds were identified in NMR data of the fresh D_2_O sample. All NMR data at 303 K were recorded on a Bruker Avance III 800 spectrometer equipped with a triple‐resonance TCI‐cryogenic probe. For both samples, the two‐dimensional NMR data, including TOCSY using 70 ms spin lock time and NOESY with 200 ms mixing time, were obtained (Jeener *et al*, 1979; Bax & Davis, 1985). All the data were processed with NMRPipe (Delaglio *et al*, 1995) and analyzed with SPARKY (http://www.cgl.ucsf.edu/home/sparky/). The three‐dimensional structure of AMSIN was calculated with CYANA ver. 2.1 (López‐Méndez & Güntert, 2006). The structural quality was evaluated with procheck‐NMR (Laskowski *et al*, 1996). The structural images were generated with MOLMOL (Koradi *et al*, 1996).

### Comparative modeling and molecular dynamics (MD) simulations

Initial structural coordinates of AMSIN‐1 and CBSIN‐2 were obtained through comparative modeling at the SWISS‐MODEL server (https://www.expasy.org/), as described previously (Zhu *et al*, 2020), in which the NMR structure of AMSIN determined here was used as the template. To generate a homodimer of AMSIN‐1, we first constructed a complex comprising two monomers by ZDOCK, an initial‐stage protein docking algorithm for the prediction of protein–protein complexes (http://zdock.umassmed.edu/). Then, a 30 ns of MD simulations for these two model peptides were performed with the GROMACS 2020.1 software package (http://www.gromacs.org/) (Gao & Zhu, 2021) using the OPLS (Optimized Potential for Liquid Simulations)‐AA/L all‐atom force field (2001 amino acid dihedrals) (Kaminski *et al*, 2001) and TIP3P model for explicit water. Solvent shell thickness was 1.5 nm in a cubic box and the total charge of the simulated systems was neutralized by adding sodium or chloride ions. Simulations were performed under periodic boundary conditions and energy minimization by the steepest descent minimization algorithm with the maximum force < 1,000.0 kJ/mol/nm. The system was subsequently equilibrated with a two‐step protocol that involved a 100‐ps NVT (Number of particles, volume, and temperature) and a 100‐ps NPT (Number of particles, pressure, and temperature), in which all protein atoms were restrained to their initial position. Following these two equilibration stages, a 30‐ns trajectory was simulated (i.e., production MD) with a time step of 2 fs. The modified Berendsen thermostat method was used to maintain the temperature at 300 K and pressure coupling was done with the Parrinello–Rahman algorithm for 1 bar. The Particle Mesh Ewald (PME) algorithm was used to treat long‐range electrostatic interactions and the Linear Constraint Solver (LINCS) algorithm to constrain all bonds. Short‐range electrostatic and short‐range van der Waals cutoffs were both 1 nm. Simulated structures were extracted from the trajectories (29.9–30 ns) and structural superimposition was performed with Swiss‐PdbViewer (https://spdbv.vital‐it.ch/).

### Antibacterial assays

Bacterial media used here included: Luria‐Bertani (LB) medium, THY medium (30 g Todd Hewitt broth and 5 g Yeast extract per liter), and tryptic soy broth (TSB) (Oxoid, Basingstoke, UK); Broth medium and brain–heart infusion (BHI) medium (Beijing Landbridge technology Limited by Share Ltd., Beijing, China); Potato dextrose (PD) medium (self‐prepared). The bacterial strains used here and their culture conditions are provided in Table EV4.

For the analysis of lysis, 1 ml of culture (*SSAN*) at OD_600_ of 0.2 was added into glass test tubes and treated with 5–10 μM AMSIN or 5 μM vancomycin for 24 h and photographed. The inhibition‐zone assay (Hultmark, 1998) was used to quantify the antibacterial activity of AMSIN. In brief, an overnight bacterial culture from a single colony was inoculated into fresh medium and grew to late log‐phase. A 10‐μl aliquot of each culture was diluted in 6‐ml pre‐heated medium containing 0.8% agar. The mixture was spread on a 9‐cm Petri dish, giving a depth of 1 mm. After settling, 3‐mm wells were punched in the plate and then 2‐μl peptide samples of different concentrations were added to each well. The agar plates were incubated overnight at indicated temperatures (Table EV4). A lethal concentration (C*_L_*) was calculated from a plot of d^2^ against log n, where d is the diameter (in cm) and n is the amount of sample applied in the well (in nmol). The plot is linear, and thus C*_L_* can be calculated from the slope (k) and the intercept (m) of this plot. The formula used here is C*_L_* = 2.93/ak10^m/k^, where a is the thickness of the bacterial plate and C*_L_* is in μM.

An inhibition‐zone assay‐based method (Gao *et al*, 2018) was used to evaluate bactericidal synergy between AMSIN and HNP1&2 or lysozyme C. In this assay, AMSIN was set to a dose (nmol/well) at which only a small inhibition zone was visible and the latter was set to a dose (nmol/well) at which no inhibition zone was formed. Synergy was identified by the inhibition zone produced by the peptide combination significantly larger than that produced by AMSIN alone.

Liquid fractional inhibitory concentration (FIC) assay was carried out according to the method previously described (Fidai *et al*, 1997; Gao *et al*, 2018). *SSAL* and *SSAN* were used in this study. The inoculum concentration of each strain was 5 × 10^5^ CFU/ml. Each well in the 96‐well microtiter tray contained 10 μl of bacteria and the two peptides at different concentrations. The tray was incubated at 37°C overnight and an FIC index was calculated according to the following formula: FIC A + FIC B = [A]/[MIC A]+[B]/[MIC B], where [A] is the concentration of AMSIN in the microtiter well that is the lowest inhibitory concentration of AMSIN in its row. [MIC A] is the MIC of AMSIN alone. FIC A is the fractional inhibitory concentration of AMSIN. [B], [MIC B], and FIC B are defined for HDP1&2 or hLZc in the same way as for AMSIN. An FIC index of ≤ 0.5, = 1 or > 1 indicates synergy, additivity, or antagonism, respectively (Fidai *et al*, 1997; Gao *et al*, 2018).

### Growth kinetics measurements

To classify AMSIN into a certain specific type of antimicrobial compounds, we chose three classical antibiotics (vancomycin, rifampicin, and kanamycin) with different interacting targets to compare their effects on the growth of *SM* with the effect of AMSIN. *SM* cells were grown in broth medium at 37°C to an optical density of OD_600_ of 0.25–0.3 and then diluted one‐fold to OD_600_ of 0.125–0.15 with the medium containing the indicated antimicrobial compounds or AMSIN (4 × C*_L_*). OD_600_ was measured on the Bioscreen C (Lab Systems, Helsinki, Finland) at 37°C with continuous shaking to record growth curves in each well that contained 200 μl of culture.

### Killing kinetics assay

Time killing curves for linear (reduced) and cyclic (oxidized) AMSIN were determined according to the literature (Schneider *et al*, 2010). In brief, *SM* was grown overnight in broth medium and diluted in fresh medium to 1 × 10^8^ CFU/ml. Peptides were added in a concentration corresponding to 5 × *C_L_* to the culture and then aliquots were taken, diluted with 0.9% NaCl, and plated onto broth medium agar plates at defined intervals (0, 15, 30, 120, and 240 min). Viable cell counts were obtained after 24 h of incubation at 37°C. Bacterial growth in the medium without peptides was used as a control.

### Membrane permeability assay

To assess the permeation ability of AMSIN on bacterial membrane, 5 × 10^5^ bacterial cells (*SM*) in 500 μl of PBS buffer (pH 7.3) were mixed with 1‐μM propidium iodide (PI) for 5 min in the dark. Fluorescence was measured with the F‐4500 FL spectrophotometer (Hitachi High‐Technology Company, Japan). Once the basal fluorescence reached a constant value, AMSIN at 10 × C*_L_* were added, and changes in fluorescence arbitrary were monitored (λexc = 525 nm; λems = 595 nm). Meucin‐18, a scorpion venom‐derived lytic peptide (Gao *et al*, 2009), was used as a positive control and vancomycin as a negative control.

### Scanning electron microscope

*BM* cells at the exponential growth phase were treated with AMSIN or vancomycin at 10 × C*_L_* at 37°C for 90 min. After centrifugation, bacterial pellets were fixed with 2.5% glutaraldehyde for 1 h, followed by washing three times with PBS buffer (pH 7.3). Dehydration was carried out with a series of graded ethanol solutions. Cells were then dried by BAL‐TEC CPD‐030 critical point dryer (Bal‐Tec AG, Balzers, Liechtenstein) before being mounted on carbon tape and sputtered with a gold coating (BAL‐TEC SCD005). Images were visualized in FEI QUANTA 450.

### RNA sequencing

*SP* R6 cells were statically cultured in THY medium at 37°C to an OD_600_ of 0.5 and then AMSIN or vancomycin was added at a subinhibitory concentration. Cells were harvested and washed with PBS buffer (pH 7.3) following further incubation for 10 min. Total RNA was isolated using the Bacteria Total RNA Isolation Kit (Sangon, China) according to the manufacturer’s instruction. RNA quality was assessed by NanoDrop and Agilent 2100 Bioanalyzer (Table EV6). After enrichment and fragment, mRNA was reverse transcribed into single‐strand DNA and then synthesized into double‐strand DNA. Following end‐filling, 5′‐phosphorylation, bubble adaptor ligation, and PCR amplification, a single‐strand circled DNA library was established for sequencing in BGISEQ‐500 sequencer (BGI, China) with a combinatorial probe‐anchor synthesis (cPAS) strategy.

### Cytoplasmic accumulation of UMP

To analyze the cytoplasmic nucleotide pool, we carried out the cytoplasmic UMP accumulation experiment according to the literature (Brötz *et al*, 1997) with some modifications. *MRSA* P1374 cells were grown in 40‐ml broth medium at 37°C to an OD_600_ of 0.5. The culture was supplemented with 130 μg/ml chloramphenicol. Following 30 min of incubation, the culture was divided into three equal portions, and one portion was treated with AMSIN (20 × C*_L_*), a second with vancomycin (20 × C*_L_*), and the third was used as an untreated control. After 30 min of incubation, UMP was extracted from the harvested cells by stirring them into boiling water. The suspension was then centrifuged (43,000 *g*, 60 min) at 4°C and the supernatant was lyophilized and dissolved in 400 μl of 50 mM ammonium acetate (pH 5.2) and adjusted to pH 2.0 with acetic acid. After acidification, the supernatant was fractionated by RP‐HPLC on Hypersil ODS (250 × 4.6 mm; Thermo Scientific) and UMP was eluted from the column with a linear gradient from 0 to 40% methanol in 50 mM NH_4_Ac (pH 5.2) within 40 min with a flow rate of 1 ml/min. UV absorbance was monitored at 260 nm. UMP was identified by ultraperformance liquid chromatography–high resolution mass spectrometry (UPLC‐HRMS) and MALDI‐TOF. Both methods yielded consistent results.

### Circular dichroism (CD) spectroscopy

Differential UV absorption of AMSIN and vancomycin in the presence or absence of UMP was studied by CD spectroscopy analysis on a Chirascan Plus spectropolarimeter v.4.4.0 (Applied Photophysics Ltd, UK). The spectra were measured at room temperature from 300 to 200 nm by using a quartz cell of 1.0 mm thickness. Data were collected at 1‐nm intervals with a scan rate of 2 nm/s. All samples were dissolved in 50 mM NH_4_OAc at the indicated concentrations: AMSIN (0.15 mg/ml); vancomycin (0.1 mg/ml); UMP (0.05 mg/ml); UMP (0.0125 mg/ml) + vancomycin (0.025 mg/ml); UMP (0.0125 mg/ml) + AMSIN (0.0375 mg/ml).

### Mass spectrometry

MALDI‐TOF was performed with an Ultraflextreme instrument (Brucker, Germany) using α‐acyano‐4‐hydroxycinnamic acid (α‐CHCA) as matrix and positive mode for AMSIN and negative mode for UMP.

Bioaffinity ESI‐MS was used to study non‐covalent interactions between AMSIN and the *SA* cell‐wall precursor—UMP. All components for interactions were dissolved in 25 mM ammonium acetate (pH5.2). Electrospray spectra were recorded with Waters SynaptG2‐Si mass spectrometer (Waters, Manchester, UK) and measurements were performed with positive ESI in resolution mode. Ions were scanned in a mass range of 500–2,000 *m/z* with the following conditions: the capillary voltage at 1,500 V; the cone voltage at 40 V; the source block temperature at 30°C. Spectra were recorded and processed by the software Masslynx4.1 (Waters, Manchester, UK).

UPLC–HRMS was used to determine the molecular weight of UMP with the UltiMate™ 3000 system (Thermo Scientific, USA) and TripleTOF^TM^ 5600 LC/MS/MS (AB SCIEX, USA). The column used was an Acquity UPLC CSH C_18_ column (1.7 μm, 2.1 × 100 mm) (Waters Corporation, Milford, MA, USA). The mobile phase A was methanol:acetonitrile:H_2_O = 1:1:1 (5 mM NH_4_Ac, pH5.2), and B was 80% isopropyl alcohol:20% methanol (5mM NH_4_Ac, pH5.2). The flow rate was 0.2 ml/min. The gradient used was as follows: 0–1 min, 60% A + 40% B; 1–14 min, 100% B; 14–18 min, 100% B; 18–18.1 min, 60% A + 40% B; 18.1–22 min, 60% A + 40% B. Optimal HRMS parameters were as follows: the electrospray voltage was 5.5 kV; the ion source temperature was 550°C; ion source gas 1 and gas 2 were both 55 psi; the curtain gas was 35 psi. The data were analyzed with LipidView^TM^ and PeakView^TM^ softwares (AB SCIEX, USA).

### Determination of the binding affinity of AMSIN to UMP by SPR

Surface plasmon resonance (SPR) analysis was performed on a Biacore T100 instrument with a CM‐5 sensor chip (GE Healthcare Life Sciences, USA) at 25°C. An amide coupling chemistry strategy was used to immobilize AMSIN on the chip surface. To this end, the chip surface was firstly activated with two injections of 1‐ethyl‐3‐(3‐dimethylaminopropyl)‐carbodiimide (EDC, 0.4 M) and N‐hydroxysuccinimide (NHS, 0.1 M) (v:v = 1:1) at a flow rate of 10 μl/min and then AMSIN solubilized in 10 mM sodium acetate (pH 5.5) at a final concentration of 25 μg/ml was injected. Non‐reacted carboxylic groups on the sensor chip surface were blocked by ethanolamine‐HCl (1 M, pH 8.5) for 420 s at a flow rate of 10 μl/min. The final immobilization level was 1,778 RUs. For detecting binding, the analyte UMP was serially diluted twofold with the running buffer PBS‐T (PBS buffer containing 0.05% Tween 20, pH 7.5) at final concentrations of 100–3.125 μM. Then, they were used to flow over the chip surface at a flow rate of 30 μl/min for 60 s. Dissociation was monitored for 120 s by injecting the running buffer suddenly after samples at a flow rate of 30 μl/min for complete removal of specifically and non‐specifically bound biological material from the surface. Responses were measured in RUs as the difference between active and reference channels. The binding curve was fitted with the software BIAevaluation v2.0.1 using 1:1 Langmuir binding model.

### Stability and hemolysis assays

To assess the stability, AMSIN was incubated in H_2_O or fresh normal mouse serum at the indicated times at 37°C and their residual activity was measured by the inhibition‐zone assay (Hultmark, 1998). Human Serum AB (Gemini Bio‐Products, Woodland, CA, USA) was used to evaluate the antibacterial activity of AMSIN in human serum without dilution. Hemolytic activity of peptides against fresh erythrocytes from the female ICR mice (*Mus musculus*) (25 g, 6 weeks of age) was assayed according to the standard method (Zhu *et al*, 2012). Peptides were diluted with 0.9% NaCl to the indicated concentration. The percentage of hemolysis is determined as (*A*_pep_–*A*_blank_)/(*A*_tot_–*A*_blank_) × 100, in which “A” represents absorbance measured at 570 nm. *A*_blank_ and *A*_pep_ were evaluated in the absence or presence of peptides. One hundred percent hemolysis (*A*_tot_) was obtained in the presence of 1% Triton X‐100.

### Cytotoxicity assay

HL‐60 and HEK 293 were used in CCK‐8 assays for the evaluation of cytotoxic effects of peptides. Cells were cultured at 37°C in 5% CO_2_: 95% air incubator in Minimum Essential Medium (MEM) supplemented with 10% (v/v) fetal bovine serum (FBS). Cells were seeded at a density of 7 × 10^3^ cells per well in 96‐well plates and incubated for 24 h. Media were then replaced with 200 μl of media containing various concentrations of peptides. The cells were incubated for 72 h and cytotoxicity was assessed with CCK‐8 Kits (Dojindo Molecular Technologies, Tokyo, Japan). Absorbance was detected at 450 nm with the TECAN Infinite M200 microplate reader (Tecan, Durham, USA).

### DNA‐binding assay

The inhibition‐zone assay was used to examine the DNA‐binding ability of AMSIN. The rationality behind this experiment is that if a reduction in the inhibition‐zone size in the presence of DNA, it would indicate that the peptide binding to the DNA causes the loss of antibacterial activity. To this end, 1.0 nmol AMSIN mixed with the salmon sperm DNA (Solarbio Life Sciences, Beijing, China) (a final concentration of 6.6 mg/ml) was added to a well of the *MRSA* P1374 plate and incubated at 37°C overnight. Inhibition zones were recorded and compared with the control without the DNA.

### Electrophysiological recordings

For the expression of Na_v_1.5, K_v_1.4, K_v_1.5, K_v_2.1, K_v_4.3, and hERG in *Xenopus* oocytes, the linearized plasmids were transcribed using the T7 or SP6 mMESSAGE‐mMACHINE transcription kit (Ambion). The harvesting of stage V–VI oocytes from an anesthetized female *Xenopus laevis* frog was previously described (Zhu *et al*, 2020). Oocytes were injected with 50 nl of cRNA at a concentration of 1 ng/nl using a micro‐injector (Drummond Scientific, USA). The oocytes were incubated in a solution containing (in mM): NaCl, 96; KCl, 2; CaCl_2_, 1.8; MgCl_2_, 2 and HEPES, 5 (pH 7.4), supplemented with 50 mg/l gentamycin sulfate. Two‐electrode voltage‐clamp recordings were performed at room temperature (18–22°C) using a Geneclamp 500 amplifier (Axon Instruments, USA) controlled by a pClamp data acquisition system (Axon Instruments, USA). Whole‐cell currents from oocytes were recorded 1–4 days after injection. Bath solution composition was (in mM): NaCl, 96; KCl, 2; CaCl_2_, 1.8; MgCl_2_, 2 and HEPES, 5 (pH 7.4). Voltage and current electrodes were filled with 3 M KCl. Resistances of both electrodes were kept between 0.7 and 1.5 M Ω. Elicited currents were sampled at 1 kHz and filtered at 0.5 kHz (for potassium currents) or sampled at 20 kHz and filtered at 2 kHz (for sodium currents) using a four‐pole low‐pass Bessel filter. Leak subtraction was performed using a‐P/4 protocol. Currents were evoked by a 100 ms (Nav) or 500 ms (Kv) depolarization to the voltage corresponding to the maximal activation of the channels in control conditions from a holding potential of −90 mV. Current traces of hERG channels were elicited by applying a +40 mV prepulse for 2 s followed by a step to −120 mV for 2 s.

### Measurement of peptide‐induced cytokine release in human‐derived cell lines

A549 and HL‐60 cells were purchased from the Cell Resource Center of the Chinese Academy of Medical Sciences (Beijing, China). They were tested for mycoplasma contamination with the Mycoplasma Detection Kit (Solarbio, China) and only negative cultures were used in our experiments. The cells were cultured in complete Dulbecco’s Modified Eagle Medium (DMEM) supplemented with 10% fetal bovine serum (FBS), penicillin (100 units/ml), and streptomycin (100 mg/ml) at 37°C in 5% CO_2_. The culture medium was changed every 2 days. Cells were passaged every 4–5 days with 0.25% trypsin (Gibco, USA). For enzyme‐linked immunosorbent assay (ELISA) (Hara *et al*, 2008), A549 or HL‐60 cells were seeded in six‐well plates at a density of 1 × 10^5^ cells/well and were incubated at 37°C in 5% CO_2_ overnight. Medium was aspirated and replaced with fresh complete DMEM supplemented with 10% FBS or serum‐free medium, both containing different concentrations of polypeptides for 24 h. Then, the supernatants were harvested for quantification of the IL‐8 amount in A549 by the Human CXCL8/IL‐8 Quantikine ELISA Kit (R&D Systems Inc., Minneapolis, MN, USA) and the MCP‐1 amount in HL‐60 by the Human MCP‐1 ELISA Kit (Boster Biological Technology Co., Ltd, Wuhan, China) as per the manufactures’ instructions.

### Detecting anti‐drug antibody (ADA) by SPR

SPR was used to evaluate the immunogenicity of AMSIN (Hermeling *et al*, 2005; Real‐Fernández *et al*, 2015). Firstly, three female ICR mice (25 g each mouse, 6 weeks of age) were injected intraperitoneally (i.p.) with 10‐μg protein dissolved in 0.9% NaCl once daily (OD) for 3 days. One female ICR mouse of 25 g (6 weeks of age) injected with 0.9% NaCl in the same manner was used as control. Blood samples were then collected from the mouse retro‐orbital plexus after 2 days. Sera were obtained by centrifugation and stored at –20°C until further use. To detect possible ADA, one aliquot of serum diluted by 1:100 by PBS‐T was used to flow over the AMSIN‐immobilized CM‐5 chip. In this process, bovine serum albumin (BSA) was added at a final concentration of 0.25 mg/ml to inhibit non‐specific binding. The amount of bound material, if any, was determined by measuring the increase in RUs. Schematic representation is provided in Appendix Fig S16A.

### Mouse lung infection model

A mouse lung infection model (Mygind *et al*, 2005; Ling *et al*, 2015) was used to evaluate the potential of AMSIN in the treatment of acute respiratory infection. To this end, ICR female mice (30 g each mouse; 9–10 weeks of age) were anesthetized with 1.25% 2,2,2‐Tribromoethanol (Avertin) (Macklin, Shanghai, China) and hung by their front teeth on a steel wire. Then, they were inoculated intranasally with 10–20 µl bacterial suspension of *SP* D39 of fresh overnight culture (1.5 × 10^6^ CFU per mouse) and then were removed from the wire to their cages. A control group of untreated mice was included. AMSIN in different doses were given 24 h after inoculation via intramuscular injection (i.m.). Mice were treated with penicillin as a positive control. At 48 h post‐infection, the mice were sacrificed and lungs removed and homogenized in 0.9% saline. Colony counts from lung homogenates were determined and the total number of colonies/lungs per mouse was calculated. Schematic representation is provided in Appendix Fig S16B.

### Mouse peritonitis model

A mouse peritonitis model (Mygind *et al*, 2005; Ling *et al*, 2015) was used to assess the *in vivo* therapeutic efficacy of AMSIN for systemic infection. In this model, AMSIN was tested against the clinical isolate *MRSA* P1374. ICR male mice (30 g each mouse; 6 weeks of age) were infected with 0.5 ml of bacterial suspension (1 × 10^8^ CFU per mouse) via intraperitoneal injection (i.p.), a dose that achieved 100% mortality within 24 h after infection. After inoculation for 1 h, treatment was initiated with AMSIN intravenous injection (i.v.) to six mice and the treatment continued for 5 days (the first day at 20 mg per kg once and the second to the fifth day at 10 mg per kg once daily). Vehicle (0.9%) treated control group included 11 mice. Schematic representation is provided in Appendix Fig S16C.

### Statistics

Statistical analyses were carried out using SPSS Statistics 17.0 (SPSS Inc.). Data are expressed as mean ± standard deviation (SD) (10 ≥ *n* ≥ 3) which were calculated by Excel. Statistical significance of means between two groups was determined by unpaired two‐tailed Student’s *t*‐test for the data that accorded with normal distribution and homogeneity of variance. For the data that did not meet any one of the two conditions, we used the non‐parametric Mann–Whitney *U*‐test to compare the difference of means between two groups (Hoffman, 2015). Before these two tests, we firstly tested data for normal distribution by Shapiro–Wilk test and homogeneity of variance by Levene’s test. Generally, *P ≤ *0.05 was considered statistically significant (*), *P ≤ *0.01 was considered highly significant (**) and *P ≤ *0.001 was considered very highly significant (***) (Chen *et al*, 2016). *P*‐values are listed in Table EV7.

## Author contributions

SZ conceived and designed research and performed gene discovery, structural, and evolutionary analyses as well as the literature search. BG performed all experiments except the NMR structure determination of AMSIN by YU and SO, the electrophysiological assay by SP and JT, and the cytotoxicity and ELISA assays by PL. The manuscript was written by SZ with assistance from all other authors. The funders of the study had no role in study design, data collection, data analysis, data interpretation, or manuscript writing.

## Conflict of interest

The authors declare that they have no conflict of interest.

**For more information**

1. The antimicrobial peptide database (APD3): https://aps.unmc.edu/
2. CD‐HIT: http://weizhongli‐lab.org/cd‐hit/
3. The microbial genomes database: https://www.ncbi.nlm.nih.gov/genome/microbes/
4. VaxiJen: http://www.ddg‐pharmfac.net/vaxijen/VaxiJen/VaxiJen.html
5. AllerTOP v. 2.0: http://www.ddg‐pharmfac.net/AllerTOP/
6. ClustalX: http://www.clustal.org/
7. Weblogo: http://weblogo.berkeley.edu/logo.cgi
8. SPARKY: http://www.cgl.ucsf.edu/home/sparky/
9. SWISS‐MODEL: https://www.expasy.org/
10. ZDOCK: http://zdock.umassmed.edu/
11. GROMACS 2020.1: http://www.gromacs.org/
12. Swiss‐PdbViewer: https://spdbv.vital‐it.ch/
13. GeneDoc: https://github.com/karlnicholas/GeneDoc/
14. SecretomeP 2.0: https://services.healthtech.dtu.dk/service.php?SecretomeP‐2.0
15. MEGA7: https://www.megasoftware.net/

## Supporting information



Appendix

Expanded View Figures PDF

Table EV1

Table EV2

Table EV3

Table EV4

Table EV5

Table EV6

Table EV7

Dataset EV1

Dataset EV2

Dataset EV3

Dataset EV4

Dataset EV5

Source Data for Figure 3

Source Data for Figure 4

Source Data for Figure 5

## Data Availability

The chemical shift data and coordinates of AMSIN were deposited to BioMagResBank (BMRB Entry 11537; https://bmrb.io/data_library/summary/?bmrbId=11537) and Protein Data Bank (accession number 2RU0; https://www.rcsb.org/structure/2RU0), respectively. RNAseq data that included the transcriptional response of *SP* R6 cells to AMSIN or vancomycin at a subinhibitory concentration and the transcriptional profile of the untreated cells were deposited to the Genbank database (accession number PRJNA778040; https://www.ncbi.nlm.nih.gov/sra/?term=PRJNA778040).
