# Supplementary material for: Adaptively evolved human oral actinomyces‐sourced defensins show therapeutic potential
Source: EMBO Mol Med. 2021 Dec 20;14(2):e14499. doi: 10.15252/emmm.202114499 (PMC8819291; doi:10.15252/emmm.202114499)
Supplement: Supplementary file 1 — Appendix [file EMMM-14-e14499-s007.pdf]

Appendix

Appendix Figure S1. Circular genome plots for five bacterial strains containing genes encoding AITD-like peptides.

Appendix Figure S2. Multiple sequence alignment of AITDs from Actinobacteria (A) and Myxobacteria (B).

Appendix Figure S3. SecP scores of precursors (A) and the net charges of the mature peptides of bacterial AITDs (B).

Appendix Figure S4. Structural comparisons between AMSIN and eukaryotic AITDs.

Appendix Figure S5. Molecular surface of the AMSIN-1 dimer.

Appendix Figure S6. Comparison of the antibacterial activity of linear and cyclic AMSIN against two oral bacteria.

Appendix Figure S7. Isolation and identification of HNP1&2 and Lysozyme C from human saliva.

Appendix Figure S8. General synergy between AMSIN and HNP1&2 or hLZc on different bacterial strains.

Appendix Figure S9. ESI-MS detecting non-covalent complexes between AMSIN and HNP1&2 (A) or hLZc (B).

Appendix Figure S10. The structure of AMSIN complexed with Lipid II based on structural replacement from the plectasin-Lipid II complex.

Appendix Figure S11. ESI-MS detecting non-covalent complexes.

Appendix Figure S12. A proposed structural basis for explaining the antibacterial synergy between HNP1 and AMSIN.

Appendix Figure S13. Inhibition-zone assay showing that AMSIN overall retained its antibacterial activity in undiluted human serum (HS).

Appendix Figure S14. The cytotoxic effects of AMSIN on HEK 293 cells.

Appendix Figure S15. Phylogenetic trees of AITDs used for testing positive selection with the maximum likelihood-based codon substitution models.

Appendix Figure S16. Schematic representation of the *in vitro* and animal experiments.

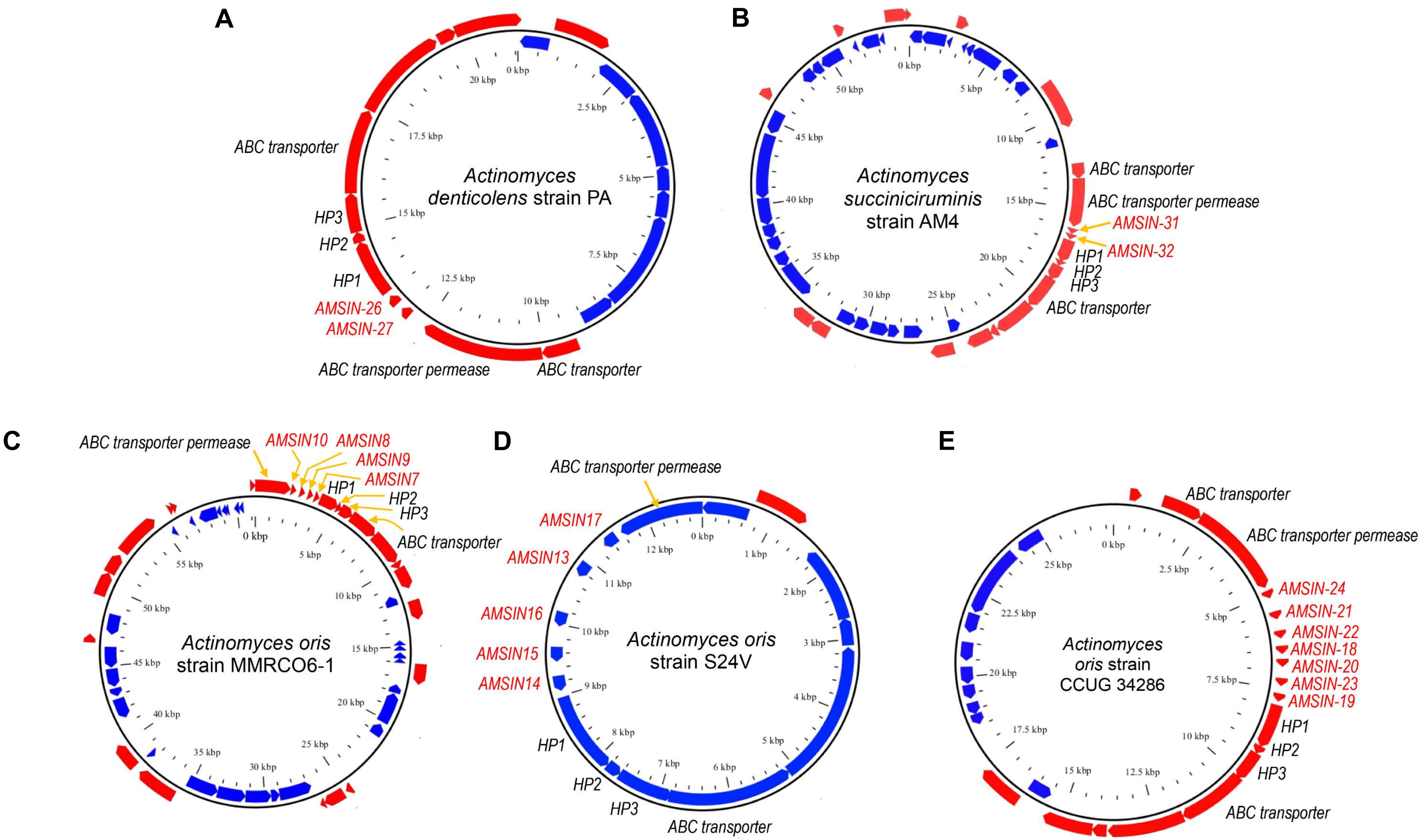

Appendix Figure S1. Circular genome plots for five bacterial strains containing genes encoding AITD-like peptides. In these GView images, red and blue tracks show genes on forward and reverse strands, respectively. AMSIN genes and their immediate upstream and downstream genes are shown. ABC, ATP binding cassette. HP, hypothetical protein. The BioProject database accession numbers for the genomes described here: PRJEB18284 (A). PRJEB6322 (B). PRJNA347929 (C). PRJNA347929 (D). PRJEB17053 (E).

A

	NCSS		Mature peptide	Identity (%)	Similarity (%)
AMSIN	*MDKFTRRTANL-VDAD--KALNAETHAPIEGA*	E	GFGC-P-WNAYECDRHCVSK-GYTGGNCRGK-IRQTCHCY---	100	100
AMSIN-7	*MDKFTRRTANL-VDAD--KALNAETHAPIEGA*	E	GFGC-P-WNAYECDRHCMSK-GYTGGNCRGK-IRQTCHCY---	97	100
AMSIN-26	*MDKFTRRTADL-AANELGDDINAETRTPLEDA*	E	GFGC-P-FNAYQCHSHCLSI-GRRGGYCRGL-VRQTCVCYR--	64	75
AMSIN-32	*MKNFIRRSSSL-DAVSFEQALRSETHAPLEGA*	E	GFGC-P-FSERSCDTHCMTK-GYRGGYCKGA-VRQTCVCYK--	59	75
AMSIN-34	*MSQFIRRTTAL-AGADFTQALRSETHAPTEGG*	E	PFGC-P-FNSFTCHRHCKSIPGYRGGYCKGR-LNQTCKCYR--	55	71
AMSIN-25	*MRKFIRRSSSL-AAASFEQALRSETQAPLEGA*	E	GFGC-P--NEYKCNRHCKSV-NYRGGYCDFWTARLRCTCY---	54	59
AMSIN-13	*MDKFTRRTAPL-SDADSKHAISSETQAPIEGA*	E	GFGC-P--NEYRCNAHCQSV-GYQGGYCDFWTARRRCTCY---	54	59
AMSIN-2	*MDKFTRRTTTL-SDSDFSQAVSSETQAPIEGT*	E	DLSC-P-WAPSVCNRHCLSH-GYRGGYCAGP-IKLVCHCY---	52	63
AMSIN-20	*MDKFTRRTAPL-SAADFKQAISSETQAPIEGT*	E	GFSC-P-GAEYACNAHCRSI-GYRGGYCGSW-LNLRCRCY---	47	52
AMSIN-11	*MDKFTRRTTTL-SDSDFSQAVSSETQAPIEGA*	E	GIGC-P-GAEYGCNKRCRSI-GYRGGYCGSL-FNLRCHCY---	47	52
AMSIN-31	*MKKFIRRSSSL-DAVSFEQALKSETHVPLEGA*	E	GFGC-P-FRPGDCYKYCRSK-GFRVGVCDSL-ANMRCHCYY--	43	56
AMSIN-8	*MDKFTRRTTTL-SDSDFSQAVSSETQAPIEGA*	E	GFSC-P-GAEYACNNRCRSI-GYRGGYCGSW-LNLQCRCY---	44	50
AMSIN-21	*MDKFTRRTAPL-SDADFKQAVSSETQAPIESA*	E	GHGC-P-ADEYRCYRDCRAM-GYRGGYCDSRTLWLRCTCY---	43	54
AMSIN-18	*MDKFTRRTAPL-SDADFKQAVSSETQAPIEGA*	E	GFGC-P-NDEYTCNAHCQSV-GYRGGYCDFWTAWRRCTCY---	48	56
AMSIN-19	*MDKFTRRTAPL-SDADFKQAVSSETQAPIEGA*	E	GFGC-P-NDEYTCNAHCQSV-GYRGGYCDFWTGWRRCTCY---	48	56
AMSIN-14	*MDKFTRRTASL-SDSDFKQAISSETHAPIEGA*	E	GFGC-P--DESRCNAHCQNN-GFDRGRCDSI-FALRCHCSYYR	35	46
AMSIN-16	*MDKFTRRAAPL-SDASFNQAISSETQAPIEGA*	E	-YGC-P-GAEYGCNNRCRSI-GYRGGYCGSL-FNLRCLCY---	41	47
AMSIN-4	*M-KFIRRSNNL-TDVSFTQALHSETRAPLEGA*	E	GYGCSPLSSDYQCTEHCRYI-GYRGGYCA-W--GIVCTCY---	42	50
AMSIN-30	*MNRFVRRSRSL-ADVTFASVLRSETHAPLEGA*	E	GHGCRPFGSDFECDEWCTYK-GYRGGYCS-W--GVVCTCYGG-	42	47
AMSIN-29	*MSRFIRRSRSL-ANINLSSTLRNEMQPPLE-A*	N	GLGC-P--NEWECDSRCRVD-GARGGYCDAWTLWTTCKCY---	45	54
AMSIN-28	*MSQFIRRSSKL-TNVTFNQSLLSETCPPLEGG*	E	GFGC-P-GQEYWCDGHCKAN-GFQYGKCDSL-FWHRCHCFE--	43	54
AMSIN-5	*MSQFIRRTSTL-TDISFSDALHSESHMPLEGA*	E	G-PC-P-HNETKCGEVCRGM-GYTGGYCHSW-FNLICKCY---	41	44
AMSIN-27	*MKQFARRTATL-ADATFTQALDSETKPPTEGA*	E	-FSC-P-MTDYPCIMHCKAI-GYRGGYCGGF-LNLSCRCH---	41	52
AMSIN-15	*MPRFVRRSTAL-ADVTFAQALHSETHAPTEGA*	E	-YNC-P-TDESPCDRHCRYS-GYRGGYCGGA-LKTSCHCY---	47	61
AMSIN-23	*MPRFVRRSTAL-ADVTFAQALHSETHPPTEGA*	E	-YNC-P-TDESPCDRHCRYS-GYRGGYCGGA-LKTSCRCY---	44	58
AMSIN-22	*MPQFVRRSTAL-ADVTFEQALHSETHAPTEGA*	E	-YNC-P-TDESPCDRHCRYS-GYRGGYCGGA-LKTSCRCY---	44	58
AMSIN-3a	*MPRFVRRSTAL-ADVTFEQALHSETHAPTEGA*	E	-YNC-P-TDESPCDRHCRYS-GYRGGYCGGI-LKTSCRCY---	44	58
AMSIN-3	*MPHFVRRSTAL-ADVTFEQALHSETHTPTEGA*	E	-YNC-P-TDESPCDRHCRYS-GYRGGYCGGI-LKTSCRCY---	44	58
AMSIN-9	*MPHFVRRSTAL-ADVTFEQALHSETHTPTEGA*	E	-YKC-P-TDESPCDRHCRYS-GYRGGYCGGI-LKTSCRCY---	44	58
AMSIN-10	*MDMFSRRRTSL-SDNRFRGTMNAETRNPLETS*	N	CFAC-P-FNEHQCHNHCLSK-GYRGGFCGGF-AAATCRCH---	50	63
AMSIN-1	*MDMFSRRRKSL-NDSRFNDAMNAETRSPLETS*	D	CFAC-P-FNEHQCHNHCLSK-GYRGGFCGGF-AAATCRCH---	50	63
AMSIN-6	*MELFSRRRKSL-SDSRFNDTMNAETRIPLETS*	D	CFAC-P-FNEQQCHNHCLST-GYRGGFCGGF-AAATCRCY---	50	58
AMSIN-12	*MELFSRRCTSL-SDSRFSDTMNAETRNPLETS*	D	CFAC-P-FNEHQCHNHCLST-GYRGGFCGGF-AAATCRCH---	47	61
AMSIN-17	*MELFSRRCKSL-SDSRFSDAMNAETRNPLETS*	D	CFAC-P-FNEHQCHNHCLST-GYRGGFCGGF-AAATCRCY---	50	61
AMSIN-24	*MELFSRRCKSL-SDSRFSDAMNAETRNPLETS*	N	CFAC-P-FNEHQCHNHCLST-GYRGGFCGGF-AAATCRCY---	50	61
AMSIN-33	*MSQFIRRTTTL-AGADFTQALRSETHAPTEGA*	E	-GPC-P-LNEKKCSQICRAK-GYKGGYCGSF-ANLVCKCY---	38	47
AMSIN-35	*MPHFIRRTTTL-AGADFTQALRSETHAPTEGA*	E	PFGC-P-ALEFVCNRHCRSIANYYKGKCVGM-FKQTCKCFSY-	43	53
AMSIN-36	*MHPLIRRTTAL-AGADFAQALRSETHAPVEGS*	E	SFPC-L-GHPARCFAHCRKA-GFRGGYCVP--I-RRCVCY---	36	44
AMSIN-39	*MPPFIRRTTAL-AGADFTQALRSETHAPVEGS*	E	SFPC-L-GHPARCVAHCRKV-GFRGGYCVP--I-RRCVCY---	36	44
AMSIN-37	*MSQFIRRTTAL-AGADFSQALRSETHAPTEGA*	E	-GPC-P-HNEGKCNRICKAK-GYHGGYCGSF-ANLVCKCYG--	40	48
AMSIN-38	*MSPFIRRTTAL-AGADFTQALRSETHAPTEGA*	E	DFGCGPFVGNGKCRRHCRNT-GRRDGYCMGM-FKQTCKCHG--	41	51
CBSIN-1	*MSTFIRNTSS--NVATIDATISNELLAPLEGA*	D	GFGC-P--DDYKCSDYCRSI-GYNNGYCSIWSFNRRCVCK---	40	51
CBSIN-2	*MPSFVLRTVS--TESTRNLNLHAIT-AP---A*	E	CCGC-P--LESRCAAYCRDNYGSRGGYCEGF-LDLRCVCI---	32	37
CBSIN-3	*MPGFALRTES--TESARNLNLHATT-AP---A*	E	CCGC-P--SEGRCAAYCRDNYNSAGGYCEGF-LDLRCVCV---	29	37
CBSIN-4	*MPTFALRAES--TQSARNTNLHATT-AP---A*	E	CCGC-P--SESRCAAYCRDNYQTAGGYCEGF-LDLRCVCV---	29	37
MMSIN	*MSHFVHRSEAVPTDGSVAVHLDQET-APP--L*	E	CCGC-P--DDNACRQYCVANYGARGGYCEGF-LDLRCVCVY--	34	47

B

	Signal peptide	Propeptide
MTSIN	*MKKTQWRALALAPMVFILSVC-----GGA*	*-EEAH-APKLS--QVR-MSQ-AE-----AAPTGDD----VTTMGGR*
CBTSIN	*MMTQKWMNGALIAVAVVLGAW--STDGIA*	*APEAHDKATTSATQVEWWSSSGERTGQQSSSSVDQSEKPNSTVGGQ*

	Mature peptide	Identity (%)	Similarity (%)
MTSIN	GHGCPRQGGECRAYCQREGHTSGGCSGFGRGECVCRDSDSVK	100	100
CBTSIN	-FGCPFNEYQCNDHCRSIGYKHGECGGFLWQECHCHRK----	30	40

Appendix Figure S2. Multiple sequence alignment of AITDs from Actinobacteria (A) and Myxobacteria (B). In panel A, putative nonclassical signal sequences (NCSSs) are boxed in green, in which the scissors denotes the cleavage site, most being occurred by an acidic residue (E or D) shown in red. In AMSIN-35, an arginine between AN (underlined) is omitted for arrangement. For the mature peptides, identical and conserved replacement sites are shadowed in black and grey, respectively. Percentages of identity and similarity were calculated with GeneDoc (https://github.com/karlnicholas/GeneDoc). Note: some of these public available sequences are also included in a recent publication without named (Sugrue *et al*, 2020).

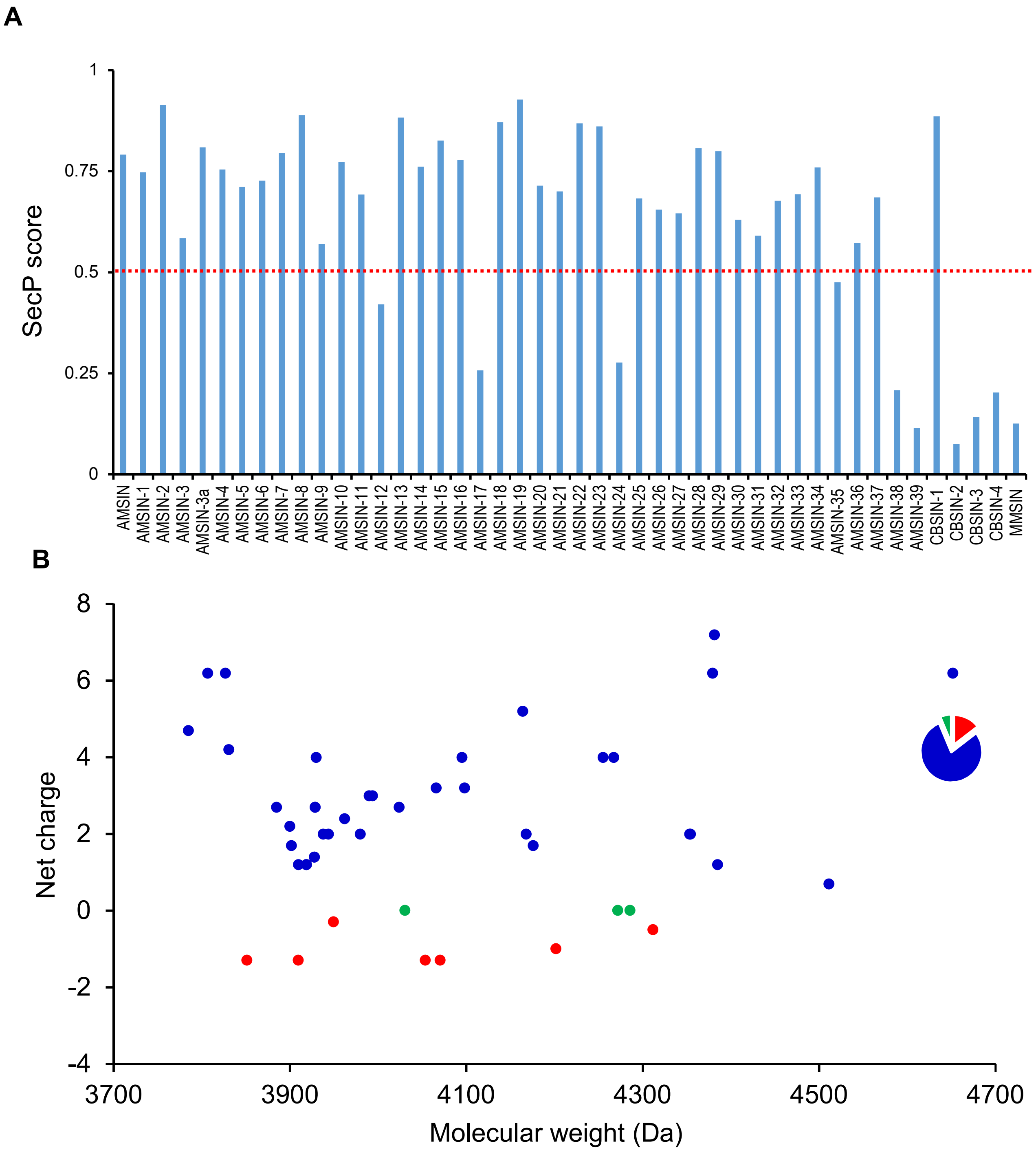

Appendix Figure S3. SecP scores of precursors (A) and the net charges of the mature peptides of bacterial AITDs (B). SecP scores were calculated by the SecretomeP 2.0 (http://www.cbs.dtu.dk/services/SecretomeP/). The red horizontal dashed line denotes the threshold ≥ 0.5 for non-classically secreted proteins. In the net charge and molecular weight relationship plot, peptides with neutral, net positive and negative charges are denoted by green, blue, and red dots, respectively. The pie chart shows the percentages of peptides with different net charges.

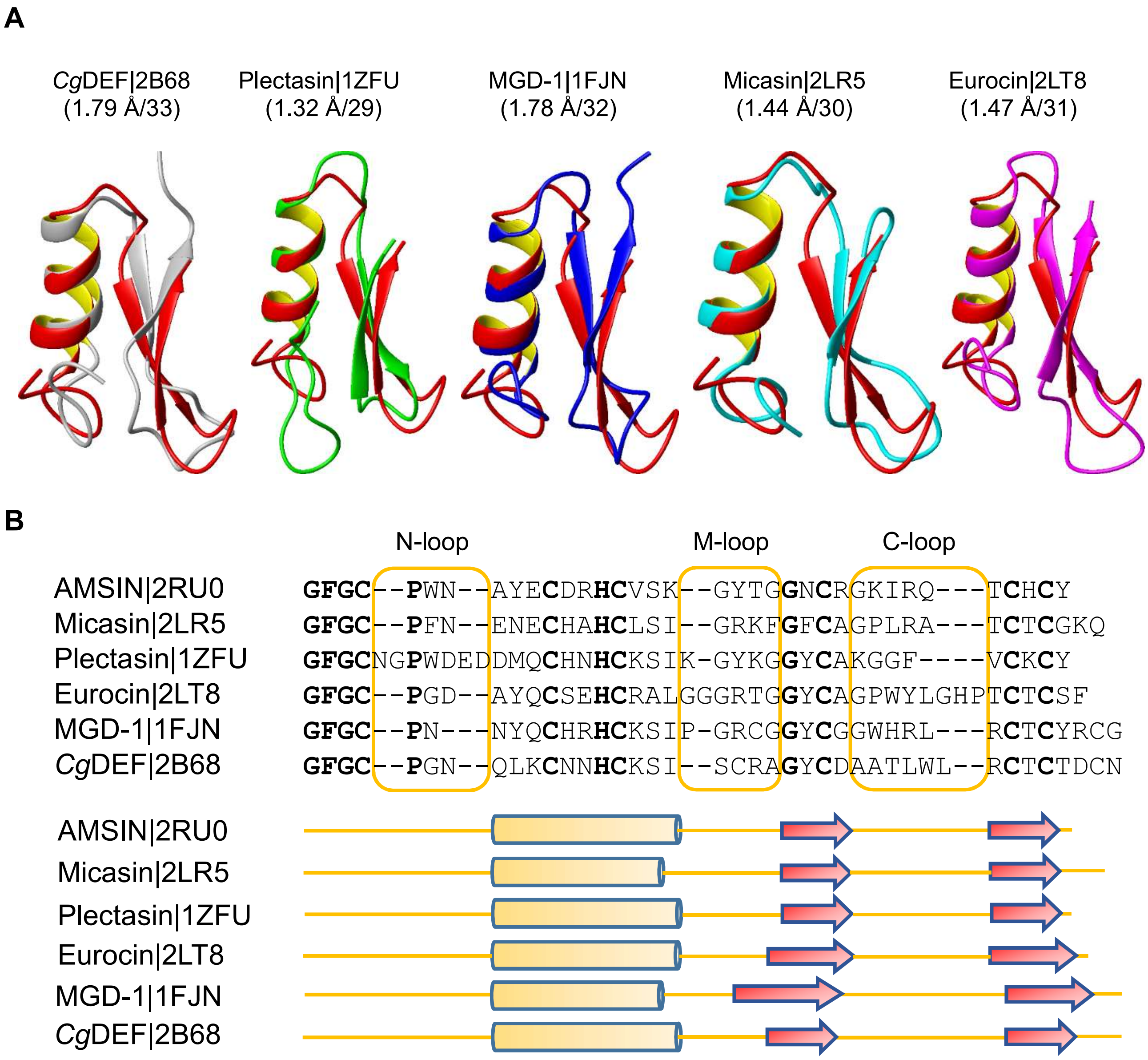

Appendix Figure S4. Structural comparisons between AMSIN and eukaryotic AITDs.

A Structural superimposition. AMSIN is colored in red and others are in different colors. Values in brackets represent root-mean-square deviation (RMSD) calculated for the aligned Cα atoms by MultiProt (http://bioinfo3d.cs.tau.ac.il/MultiProt/).

B Structure-based sequence alignment showing the conservation of secondary structural elements in AITDs (α-helix, cylinder; β-strand, arrow). Amino acid residues conserved across the alignment are shadowed in yellow and the flexible loops are boxed in orange. The elements below the sequence alignment are extracted from their experimental coordinates (see the PDB entries following their names).

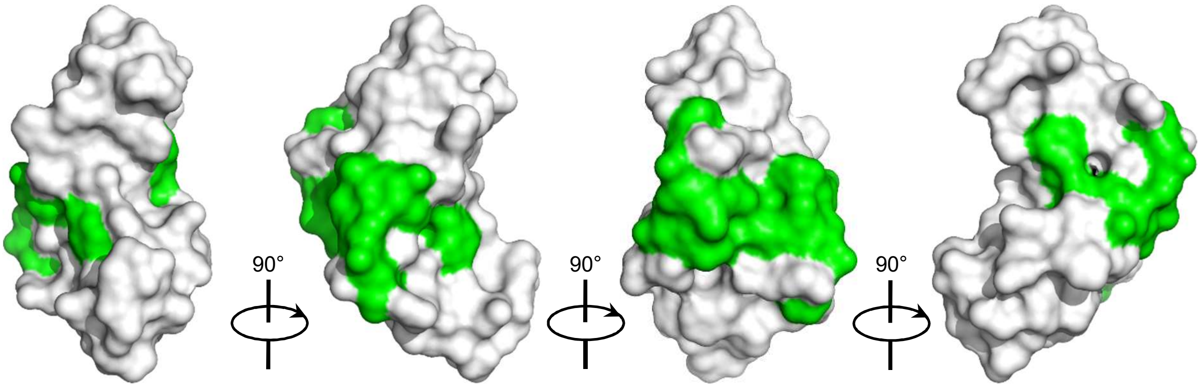

Appendix Figure S5. Molecular surface of the AMSIN-1 dimer. Hydrophobic residues located on the N-terminus and the C-loop of this peptide (see Fig 1D) are shown in *green*. The four images are continuously rotated 90° along the *y* axis.

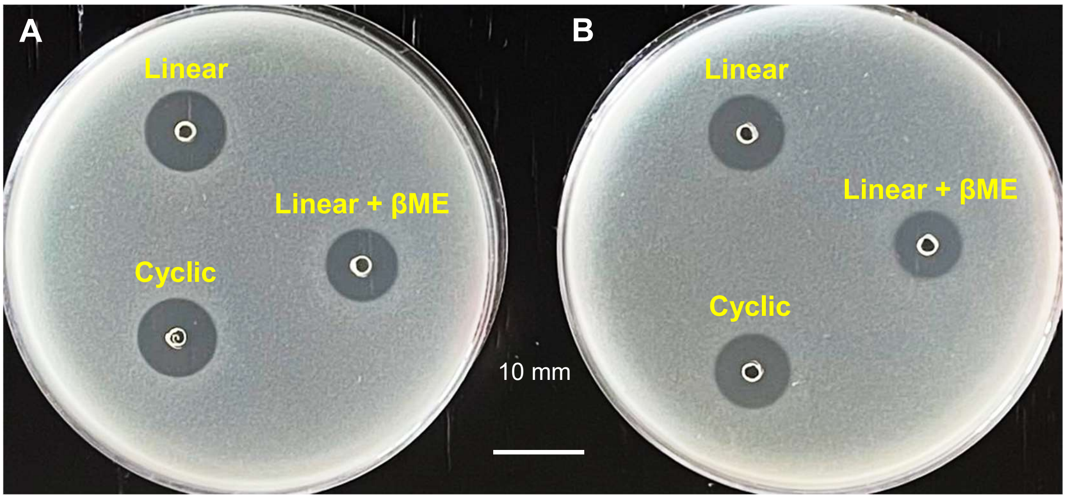

Appendix Figure S6. Comparison of the antibacterial activity of linear and cyclic AMSIN against two oral bacteria. The dose of peptides used here was 0.5 nmol/well. To ensure that the linear AMSIN stayed in the non-cyclized (reduced) form, we added 5 mM β-mercaptoethanol (βME) in one well with the linear peptide. (A) *Streptococcus mutans*. (B) *Streptococcus salivarius*.

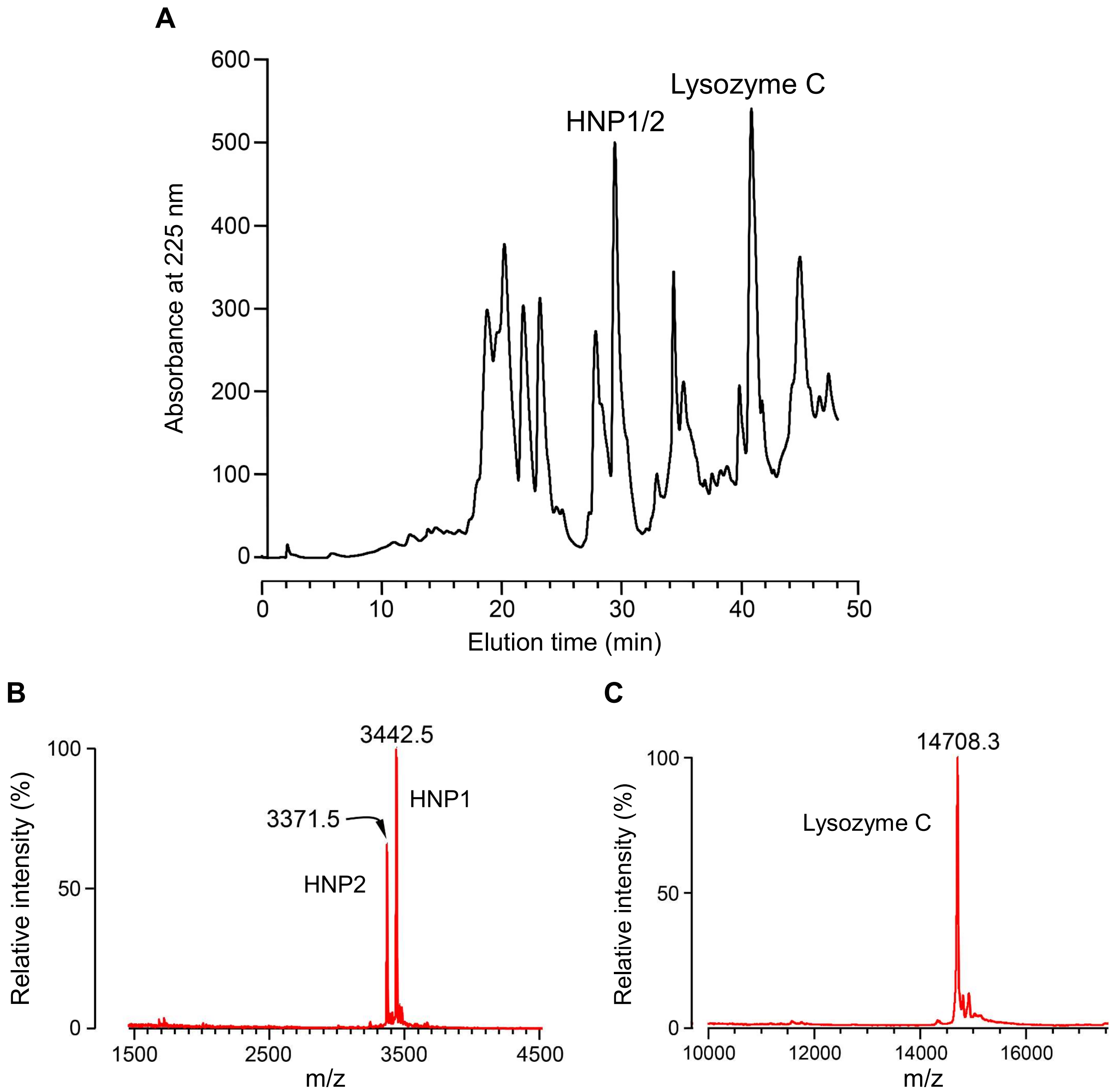

Appendix Figure S7. Isolation and identification of HNP1&2 and Lysozyme C from human saliva.

A RP-HPLC. The saliva was first extracted by 10% acetic acid and then the acidic extract was subjected to RP-HPLC analysis.

B, C MALDI-TOF identification of three previously known human saliva antibacterial factors (HNP1&2 and lysozyme C).

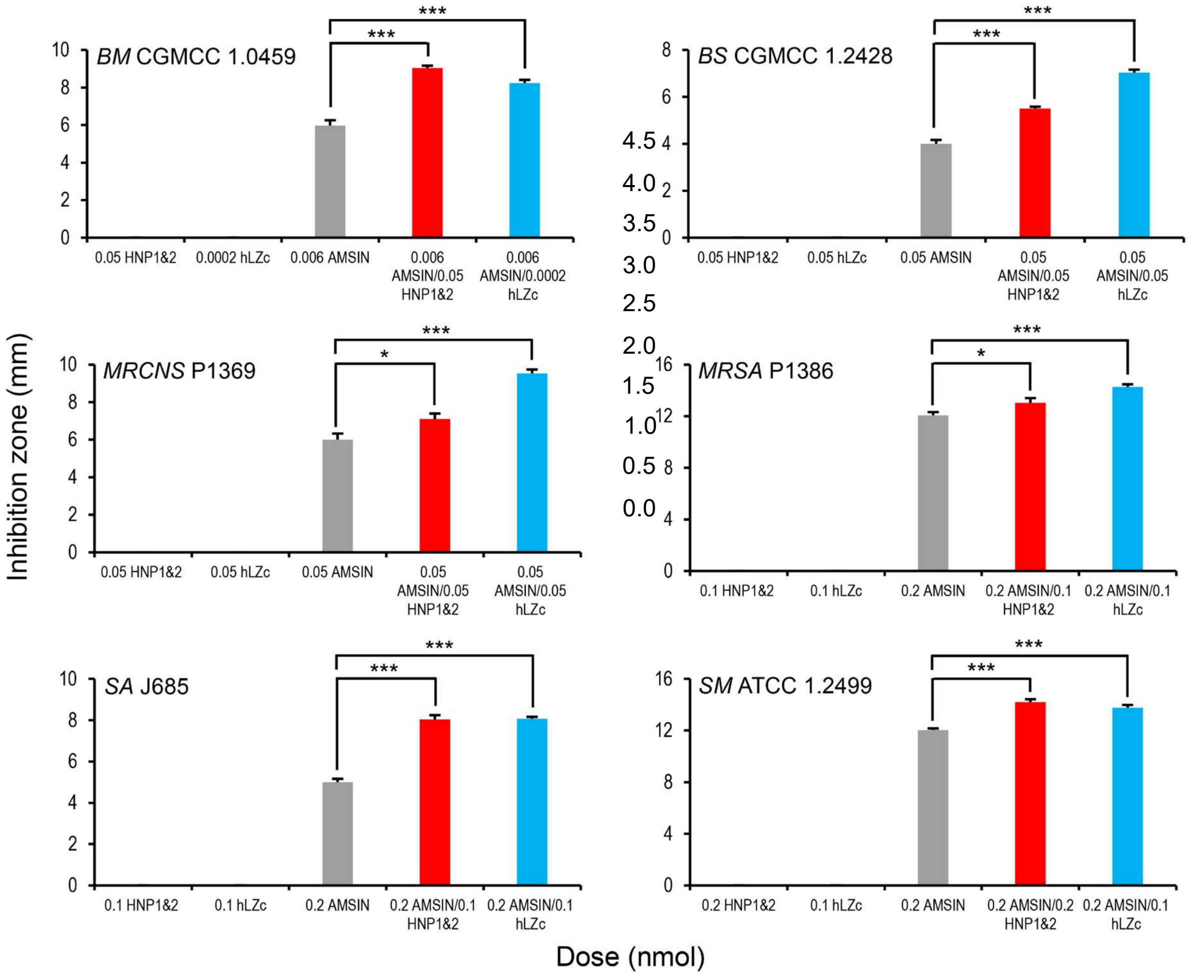

Appendix Figure S8. General synergy between AMSIN and HNP1&2 or hLZc on different bacterial strains.

Abbreviations for species names of bacteria: *BM*, *B. megaterium*; *BS*, *B. subtilis*; *MRCNS*, methicillin-resistant coagulase-negative *Staphylococcus*; *MRSA*, methicillin-resistant *S. aureus*; *SA*, *S. aureus*; *SM*, *S. mutans*. Mean ± SD of three biological replicates is displayed. *P* values were obtained by Student's t-test (**P*<0.05, ****P*<0.001, ns: no significance; exact *P* values are listed in Table EV7).

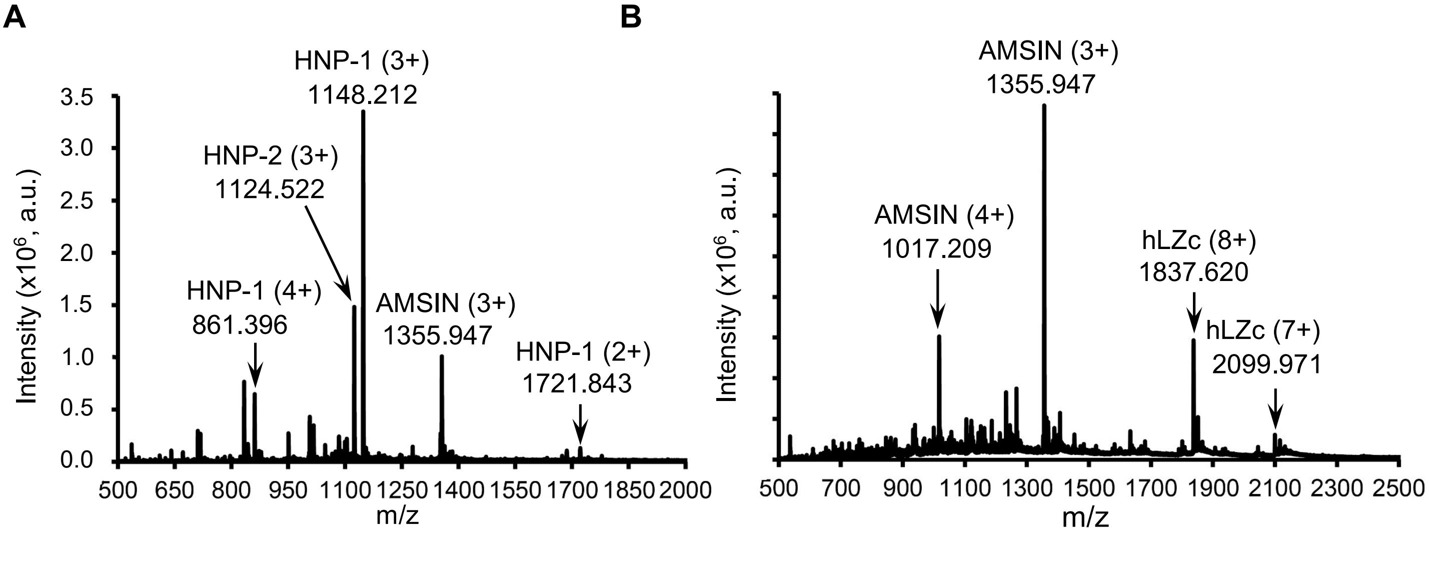

Appendix Figure S9. ESI-MS detecting non-covalent complexes between AMSIN and HNP1&2 (A) or hLZc (B).

A The mixture of 52.5 µM AMSIN and 73.5 µM HNP1&2 in 25 mM NH4OAc (pH5.2).

B The mixture of 52.5 µM AMSIN and and 50 µM hLZc in 25 mM NH4OAc (pH5.2).

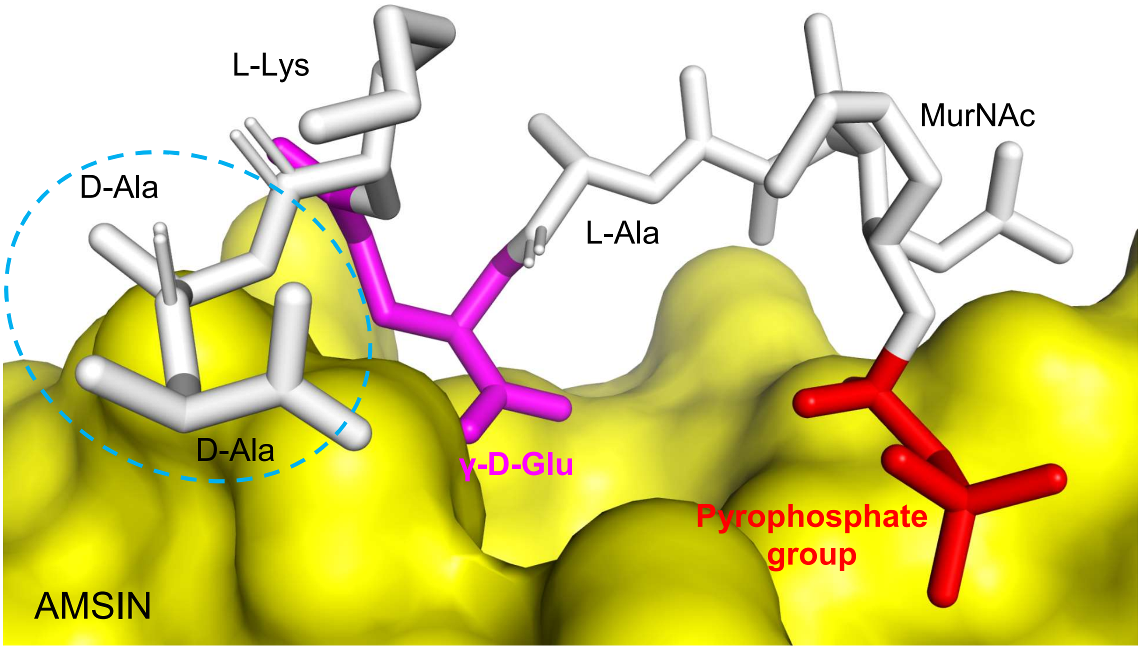

Appendix Figure S10. The structure of AMSIN complexed with Lipid II based on structural replacement from the plectasin-Lipid II complex. For clarity, only the structural units corresponding to the pentapeptide attached to the MurNAc amino sugar are shown. In this complex, the γ-D-glutamic acid and the pyrophosphate group predicted to bind AMSIN (displayed as yellow molecular surface) are shown as colorful sticks and the two C-terminal alanines for vancomycin binding is circled by a cyan dashed circle.

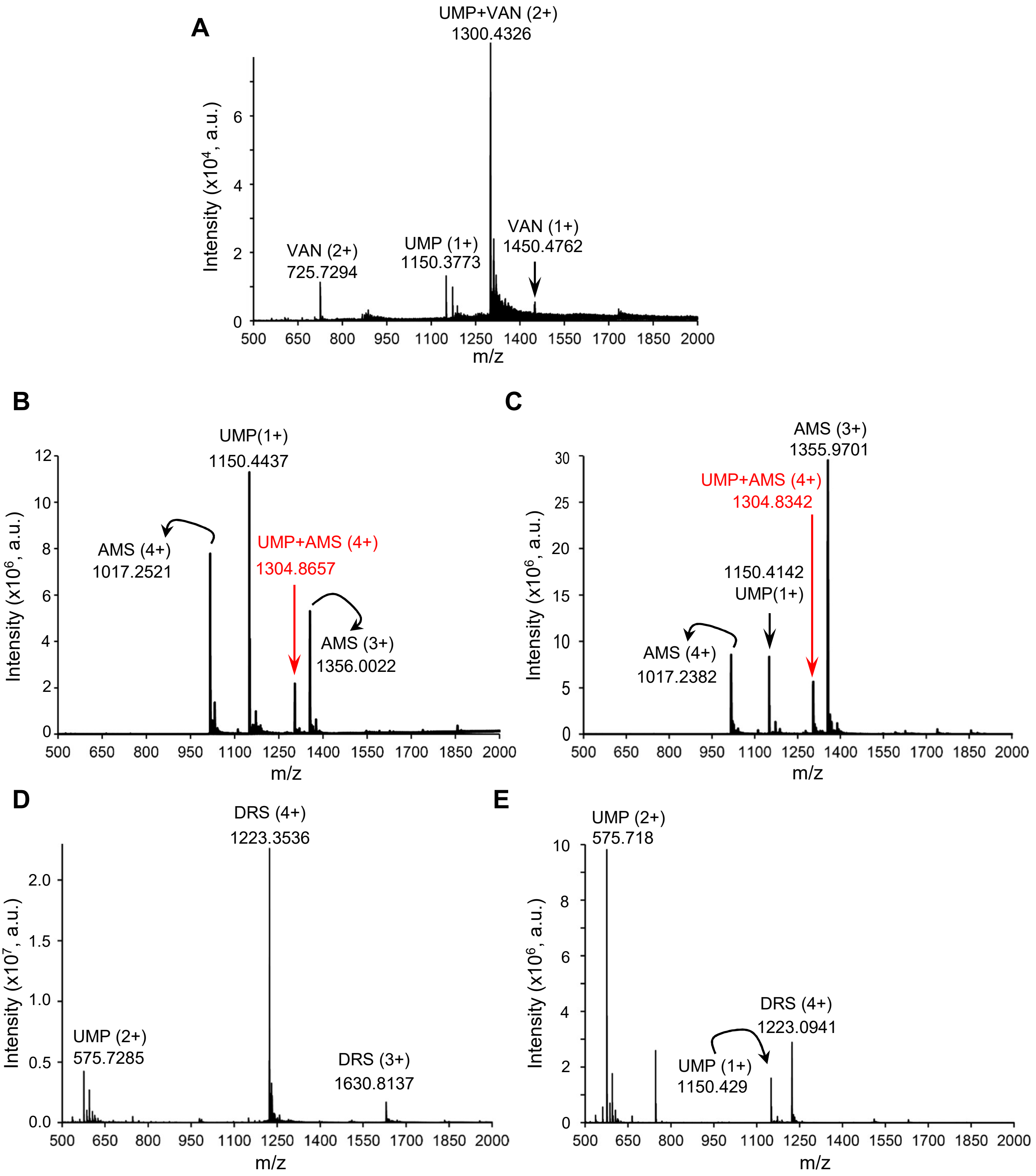

Appendix Figure S11. ESI-MS detecting non-covalent complexes.

A The mixture of 25 μM UMP and 673 μM vancomycin (VAN).

B The mixture of 125 μM UMP and 154 μM AMSIN.

C The mixture of 25 μM UMP and 308 μM AMSIN.

D The mixture of 25 μM UMP and 212 μM drosomycin (DRS).

E The mixture of 125 μM UMP and 106 μM DRS.

These components were dissolved in 25 mM ammonium acetate (pH5.2). Singly, doubly, triply and quadruply protonated species are denoted as 1+, 2+, 3+ and 4+.

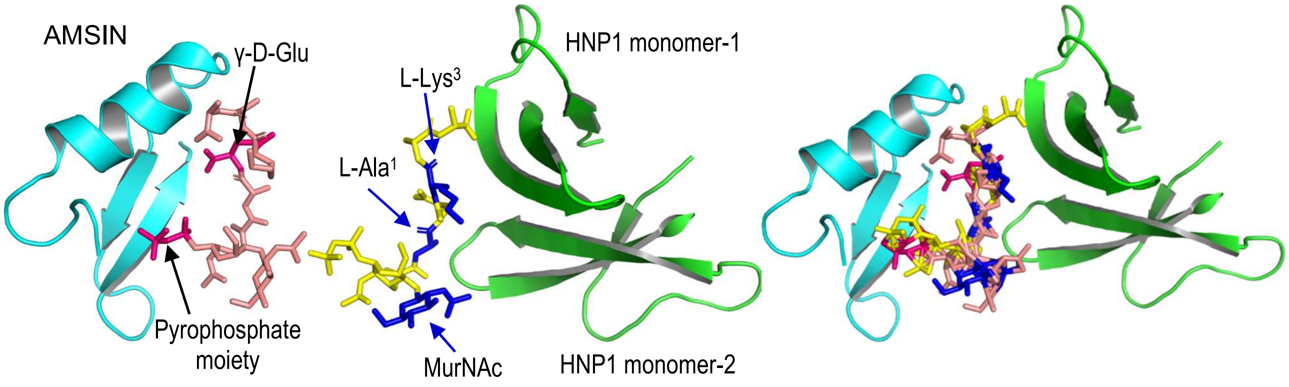

Appendix Figure S12. A proposed structural basis for explaining the antibacterial synergy between HNP1 and AMSIN. The AMSIN-Lipid II complex (left) and the previously published HNP1-Lipid II complex (Oppedijk *et al*, 2016) (middle) is superimposed over their bound Lipid II (right). The peptides are displayed as cartoons and Lipid II as sticks. For clarity, bactoprenol not involved the interactions is omitted. Arrows denote the Lipid II elements involved in peptide binding (hot pink for AMSIN and blue for HNP1).

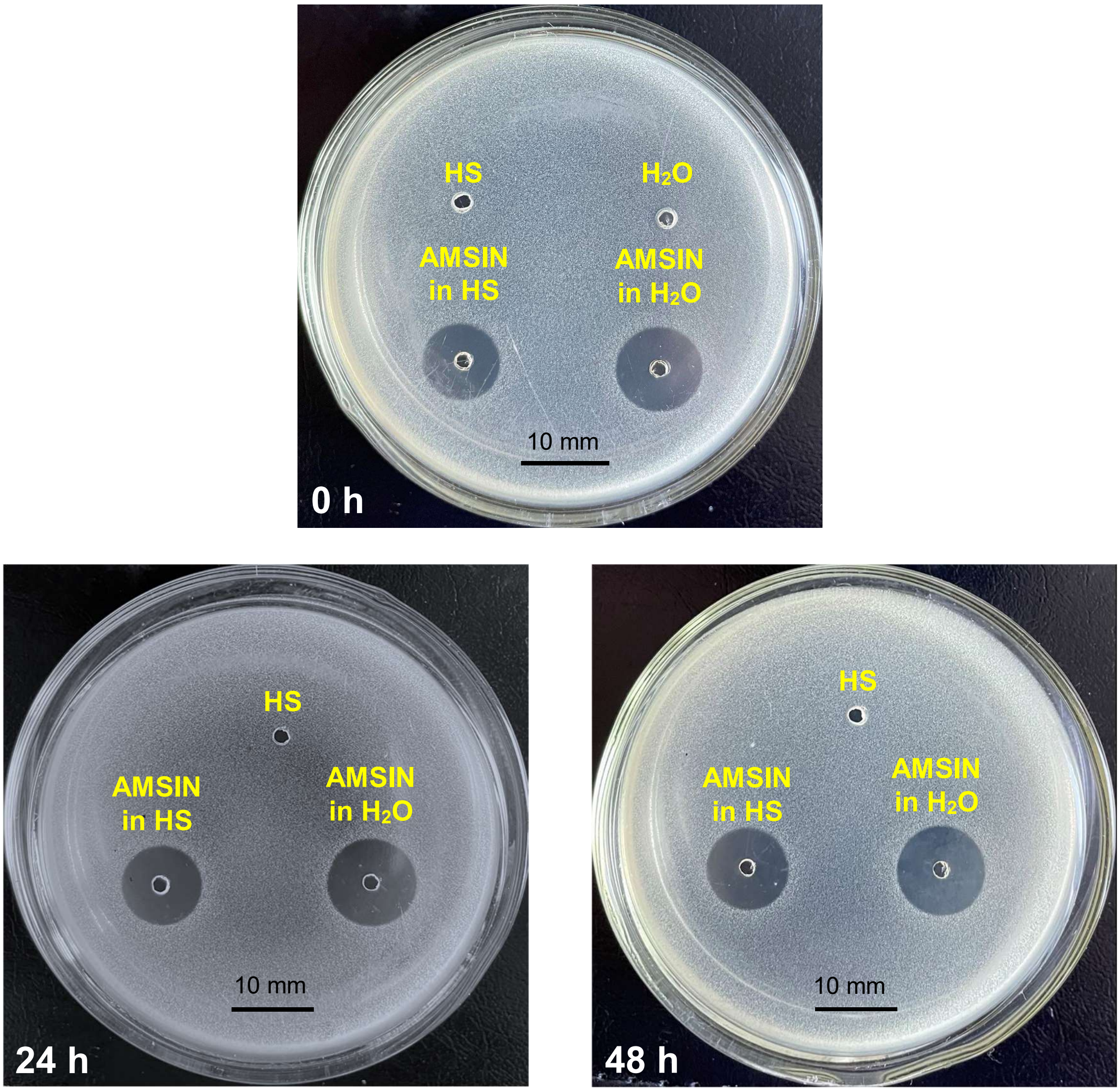

Appendix Figure S13. Evaluation of the antibacterial activity of AMSIN after treated with undiluted human serum (HS). The assay time was set at 0, 24 and 48 h and the temperature was 37 °C. Treatment with water was used as comparison. The bacterium used was MRSA P1374 and the peptide dose was 1.0 nmol/well.

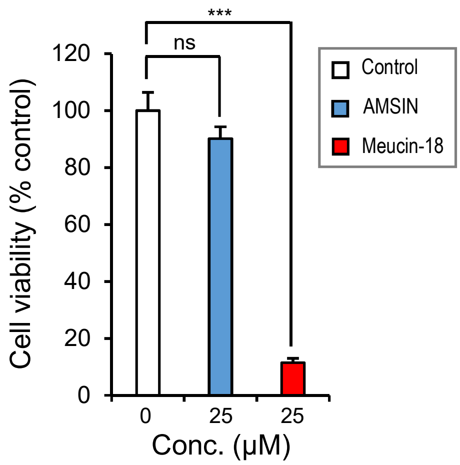

Appendix Figure S14. The cytotoxic effects of AMSIN on HEK 293. Mean ± SD of three biological replicates is displayed. *P* values were obtained by Student's t-test (****P*<0.001, ns: no significance; exact *P* values are listed in Table EV7).

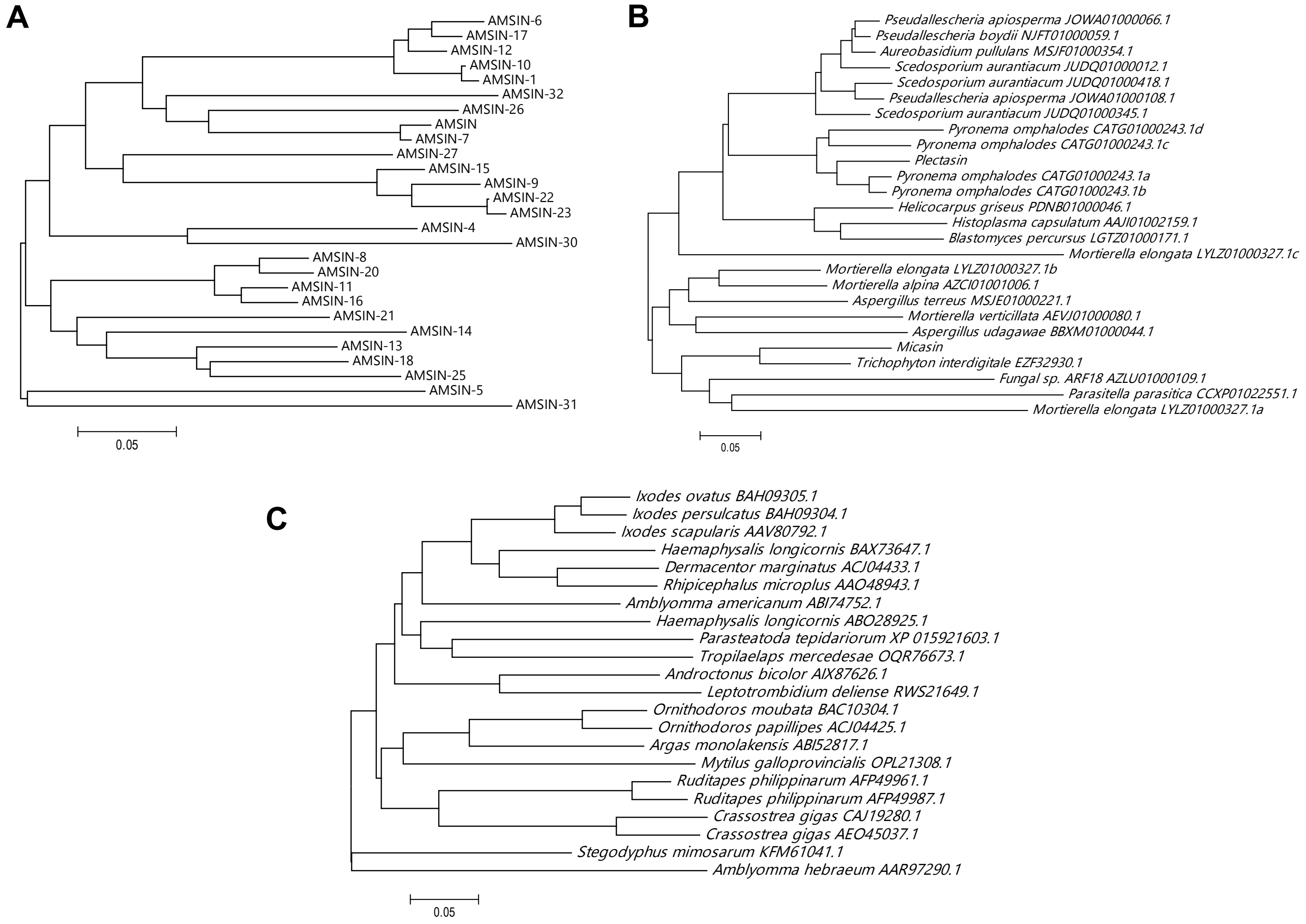

Appendix Figure S15. Phylogenetic trees of AITDs used for testing positive selection with the maximum likelihood-based codon substitution models. These trees were constructed based on gene sequences and inferred by the neighbor joining method with *p* distance to compute the evolutionary distances (NJp method) in the units of the number of base differences per site, which is implemented in MEGA7 (https://www.megasoftware.net/). They are drawn to scale with branch lengths in the same units as those of used to infer the phylogenetic tree. Most peptides in panels B and C are referred to as their species names followed by their GenBank accession numbers to minimize confusion. (A) Actinomyces. (B) Fungi. (C) Animals.

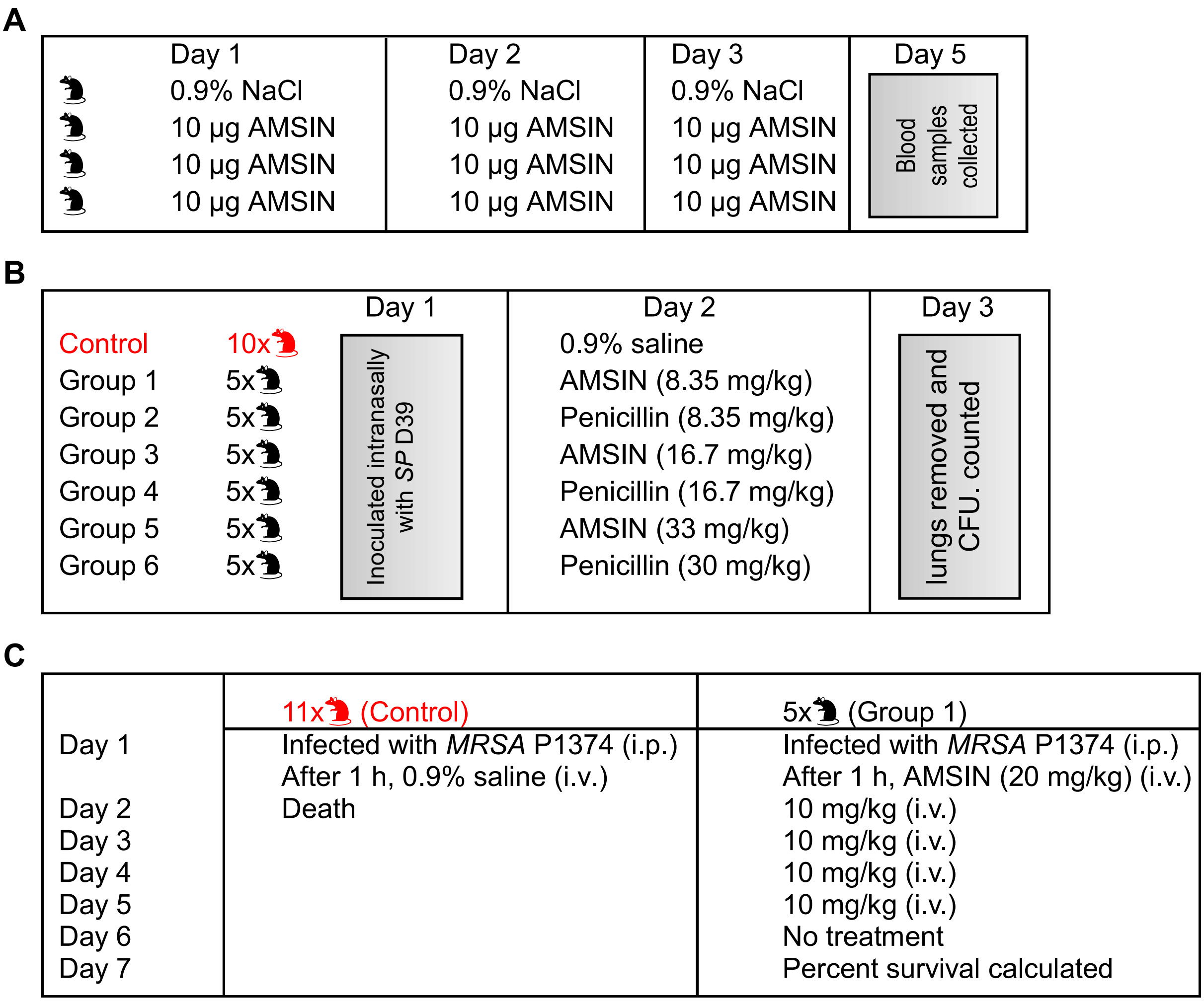

Appendix Figure S16. Schematic representation of the *in vitro* and animal experiments.

A Detecting anti-drug antibody (ADA) by SPR.

B Mouse lung infection model.

C Mouse peritonitis model.
