# Supplementary material for: Adaptively evolved human oral actinomyces‐sourced defensins show therapeutic potential
Source: EMBO Mol Med. 2021 Dec 20;14(2):e14499. doi: 10.15252/emmm.202114499 (PMC8819291; doi:10.15252/emmm.202114499)
Supplement: Supplementary file 2 — Expanded View Figures PDF [file EMMM-14-e14499-s017.pdf]

Expanded View Figures

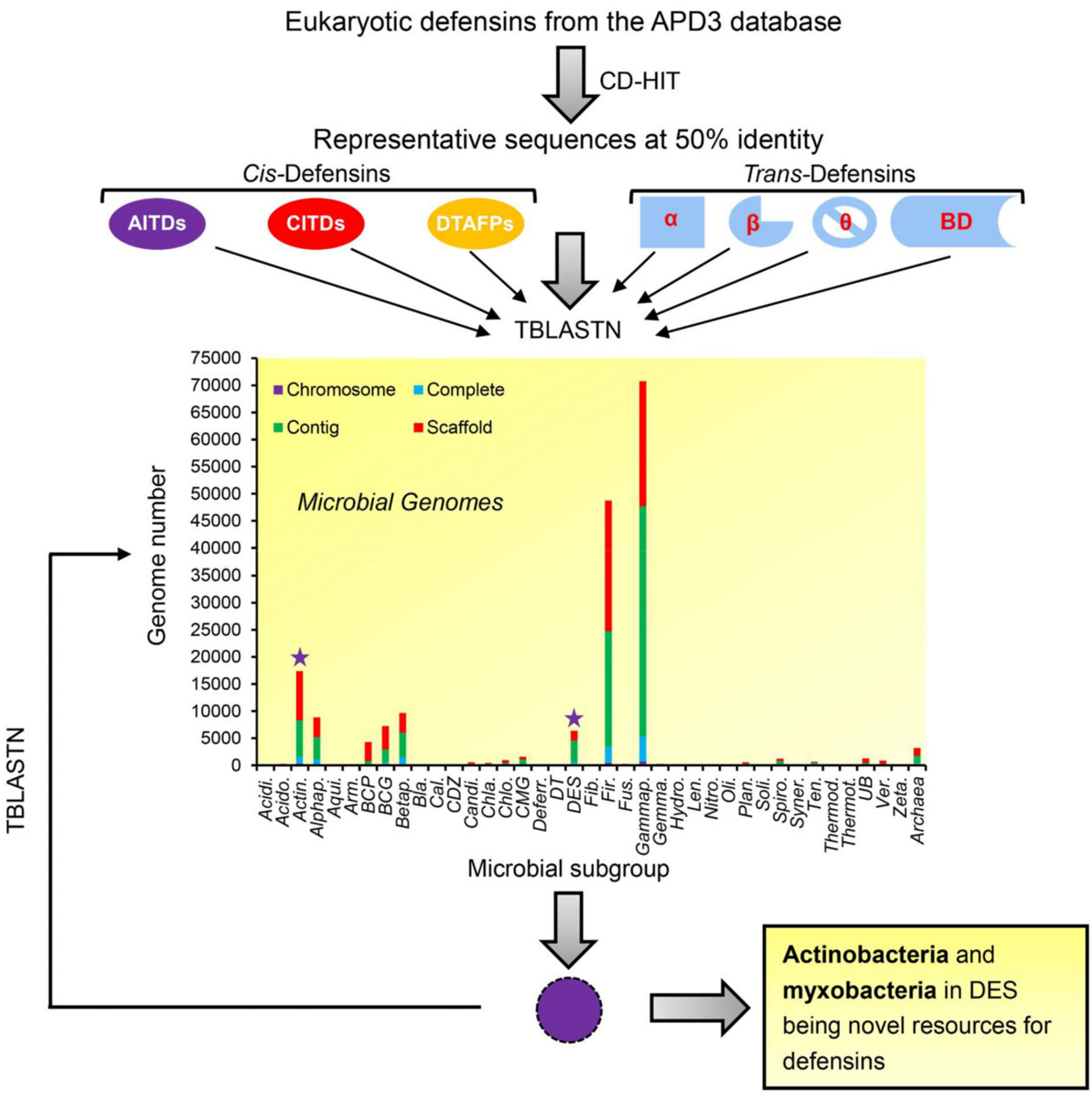

Figure EV1. Database search for discovery of bacterial homologs of eukaryotic defensins.

Queries extracted from the APD3 database included *cis*- and *trans*-defensins (Shafee *et al*, 2016; Zhou *et al*, 2019) and their redundancy was removed by CD-HIT to 50% identity. The sign "★" denotes the microbial subgroups identified as the sources of bacterial defensin-like peptides. "Actin." and "DES" are the abbreviations of actinobacteria and delta/epsilon subdivisions, respectively. For other abbreviations, see Dataset EV1.

© 2021 The Authors

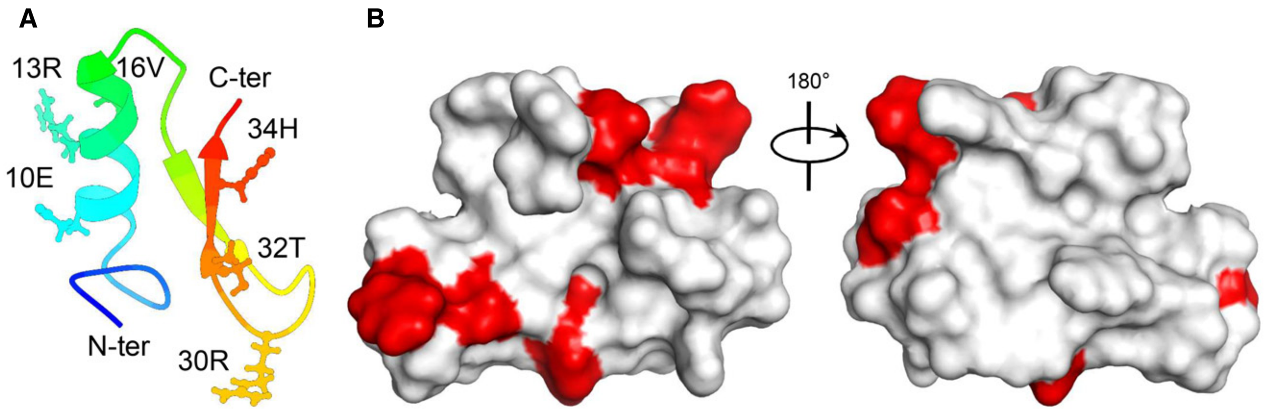

Figure EV2. Mapping of PSSs of the actinomyces-sourced defensins on the structure of AMSIN.

A The ribbon drawing of AMSIN with PSSs shown as ball-and-stick model.

B The molecular surface of AMSIN. PSSs are marked in red.

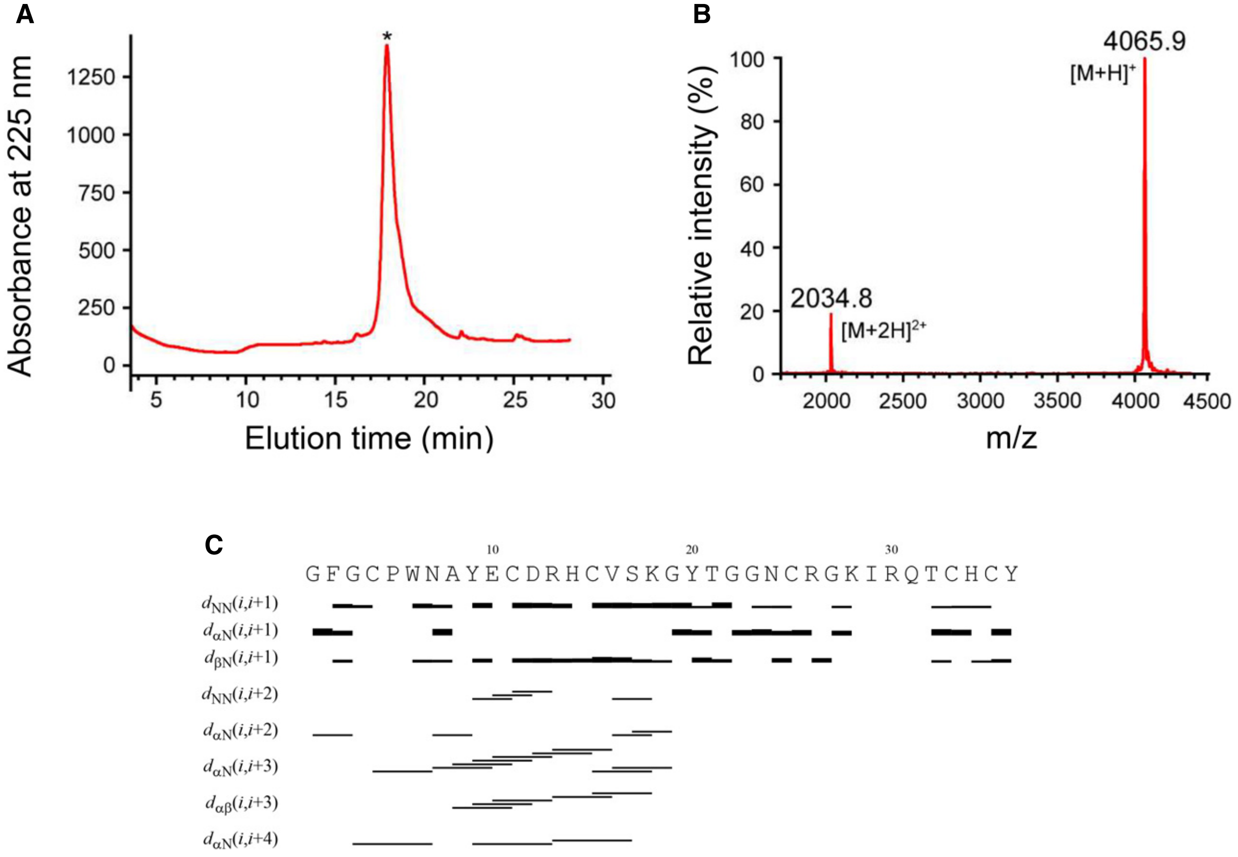

Figure EV3. Oxidative refolding and identification of AMSIN.

A Reversed-phase high-performance liquid chromatographic (RP-HPLC) profile of oxidized AMSIN (marked by an asterisk).

B MALDI-TOF MS. The two main peaks correspond to the singly and doubly protonated forms of AMSIN, respectively.

C Summary of nuclear Overhauser effect (NOE) data of AMSIN. The thickness of the bars indicates the intensity of the NOEs.

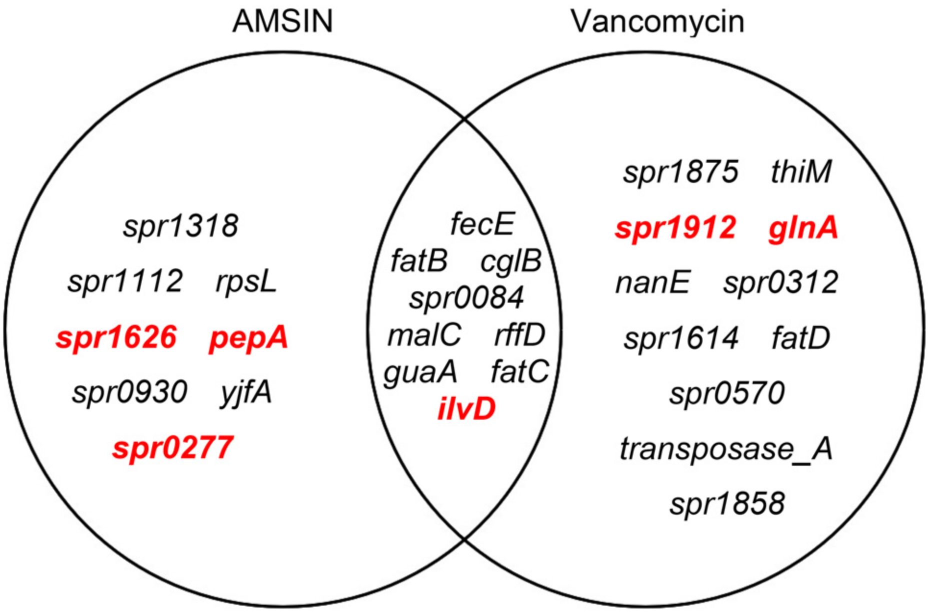

Figure EV4. Venn diagram for comparison of the AMSIN and vancomycin stimulon of *S. pneumoniae* R6.

The upregulated and downregulated genes stimulated by a sub-lethal concentration of antibacterial agents are shown in red and black, respectively.

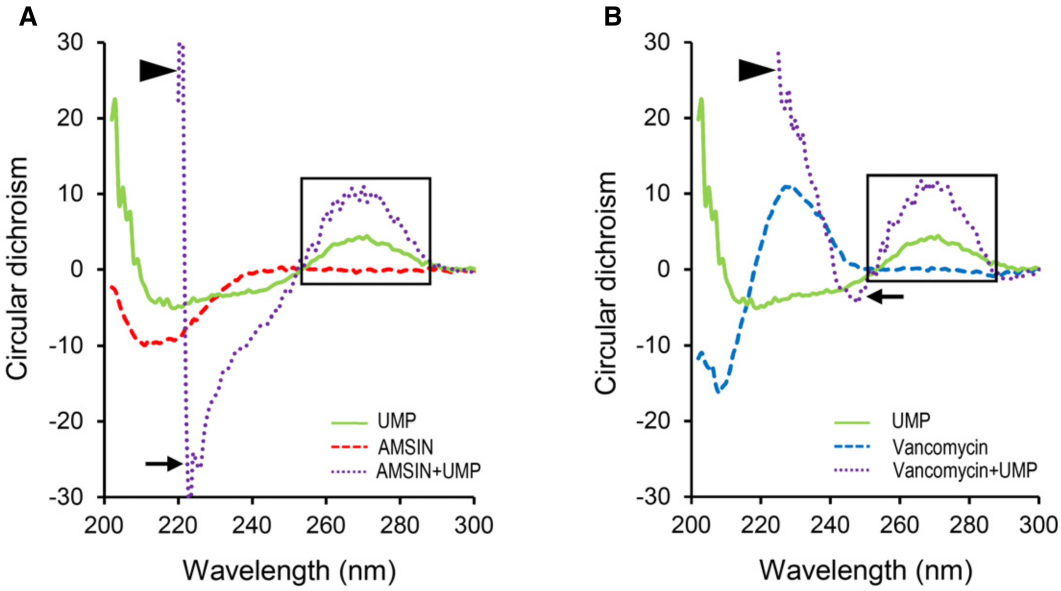

Figure EV5. CD spectra of AMSIN and vancomycin in the absence or presence of UMP.

A, B In these two mixtures, emerging maximum and minimum CD absorbance peaks are marked by triangle and arrow, respectively, and the positive band around 270 nm common to UMP is boxed. (A) AMSIN. (B) Vancomycin.

© 2021 The Authors
