# Supplementary material for: Adaptively evolved human oral actinomyces‐sourced defensins show therapeutic potential
Source: EMBO Mol Med. 2021 Dec 20;14(2):e14499. doi: 10.15252/emmm.202114499 (PMC8819291; doi:10.15252/emmm.202114499)
Supplement: Supplementary file 3 — Table EV1 [file EMMM-14-e14499-s006.docx]

**Table EV1.** Maximum likelihood estimates of parameters in fungal AITDs

Model S *p l* Estimates of parameters PSSs

M0 (one-ratio) 24.04 1 -1643.69 *ω* = 0.28 None

M1 (Nearly Neutral) 23.27 2 -1581.13 *p*_0_ = 0.29 (*p*_1_ = 0.71) Not allowed

*ω*_0_ = 0.02 (*ω*_1_ = 1.00)

M2 (Positive Selection) 23.27 4 -1581.13 *p*_0_ = 0.29 None

*p*_1_ = 0.54 (*p*_2_ = 0.17)

*ω*_0_ = 0.02

*ω*_1_ = 1.00 (*ω*_2_ = 1.00)

M7 (beta) 31.68 2 -1559.82 *p* = 0.38, *q* = 0.7 Not allowed

M8 (beta&ω>1) 31.68 4 -1559.82 *p* = 0.38, *q* = 0.75 None

*p*_0_ = 1.00 (*p*_1_ = 0.00)

*ω*_s_ = 1.00

Note: Twice the log likelihood difference (2Δ*l*) are 0 between M1 and M2 (p value = 1) and between M7 and M8 (p value = 1).
