# Supplementary material for: Adaptively evolved human oral actinomyces‐sourced defensins show therapeutic potential
Source: EMBO Mol Med. 2021 Dec 20;14(2):e14499. doi: 10.15252/emmm.202114499 (PMC8819291; doi:10.15252/emmm.202114499)
Supplement: Supplementary file 4 — Table EV2 [file EMMM-14-e14499-s010.docx]

**Table EV2.** Maximum likelihood estimates of parameters in animal AITDs

Model S *p l* Estimates of parameters PSSs

M0 (one-ratio) 31.57 1 -1596.21 *ω* = 0.09 None

M1 (Nearly Neutral) 24.37 2 -1569.13 *p*_0_ = 0.63 (*p*_1_ = 0.37) Not allowed

*ω*_0_ = 0.06 (*ω*_1_ = 1.00)

M2 (Positive Selection) 24.37 4 -1569.13 *p*_0_ = 0.63 None

*p*_1_ = 0.37 (*p*_2_ = 0.00)

*ω*_0_ = 0.06

*ω*_1_ = 1.00 (*ω*_2_ = 4.26)

M7 (beta) 35.92 2 -1519.35 *p* = 0.43, *q* = 3.18 Not allowed

M8 (beta&ω>1) 35.92 4 -1519.35 *p* = 0.43, *q* = 3.18 None

*p*_0_ = 1.00 (*p*_1_ = 0.00)

*ω*_s_ = 3.90

Note: Twice the log likelihood difference (2Δ*l*) are 0 between M1 and M2 (p value = 1) and between M7 and M8 (p value = 1).
