# Supplementary material for: Adaptively evolved human oral actinomyces‐sourced defensins show therapeutic potential
Source: EMBO Mol Med. 2021 Dec 20;14(2):e14499. doi: 10.15252/emmm.202114499 (PMC8819291; doi:10.15252/emmm.202114499)
Supplement: Supplementary file 5 — Table EV3 [file EMMM-14-e14499-s003.docx]

**Table EV3.** Input NMR data and structural statistics for the final 20 structures of AMSIN

Number of experimental restraints

Total Number of NOEs 397

Short-range | i - j | ≤ 1 235

Medium range 1 < | i - j | < 5 87

Long range 5 ≤ | i - j | 75

Number of dihedral angle restraints (ϕ) 17

Hydrogen bond restraints 8

Structure statistics, 20 conformers

CYANA target function value (Å2) 0.38 ± 0.01

Maximum residual distance constraint violation (Å) 0.08 ± 0.01

Average pairwise r.m.s.d. (Å)

Backbone atoms (residues 4-35) 0.28 ± 0.10

Heavy atoms (residues 4-35) 0.87 ± 0.14

PROCHECK Ramachandran plot analysis

Residues in favored regions (%) 67.9

Residues in additionally allowed regions (%) 22.3

Residues in generously allowed regions (%) 9.8

Residues in disallowed regions (%) 0.0
