# Supplementary material for: Adaptively evolved human oral actinomyces‐sourced defensins show therapeutic potential
Source: EMBO Mol Med. 2021 Dec 20;14(2):e14499. doi: 10.15252/emmm.202114499 (PMC8819291; doi:10.15252/emmm.202114499)
Supplement: Supplementary file 6 — Table EV4 [file EMMM-14-e14499-s002.docx]

**Table EV4.** Sources and culture conditions of the bacteria used in this study

**Strains Sources Culture conditions**

*Bacillus megaterium* CGMCC 1.0459 Center for Microbial Resources, Institute of Microbiology, Beijing, China LB, 37°C

*Bacillus subtilis* CGMCC 1.2428 Center for Microbial Resources, Institute of Microbiology, Beijing, China LB, 37°C

*Staphylococcus aureus* CGMCC1.89 MSSA Center for Microbial Resources, Institute of Microbiology, Beijing, China Broth Medium, 37°C

*S. epidermidis* PSSE P1111 302^nd^ Military Hospital, Beijing, China Broth Medium, 37°C

*Staphylococcus* MRCNS P1369 302^nd^ Military Hospital, Beijing, China Broth Medium, 37°C

*S. aureus* MRSA P1374 302^nd^ Military Hospital, Beijing, China Broth Medium, 37°C

*S. aureus* PRSA P1383 302^nd^ Military Hospital, Beijing, China Broth Medium, 37°C

*S. aureus* MRSA P1386 302^nd^ Military Hospital, Beijing, China Broth Medium, 37°C

*S. epidermidis* PRSE P1389 302^nd^ Military Hospital, Beijing, China Broth Medium, 37°C

*Staphylococcus aureus*  Gifted from Dr. Jing Qi (Shandong Academy Broth Medium, 37°C

(Strain J685,J698,J700 J706,J708,J710) of Agricultural Sciences, Jinan, China)

*Streptococcus pneumoniae* Gifted from Prof. Jingren Zhang (Division of

(Strain D39, R6, ST556, TIGR4) Medicine, Tsinghua University) THY^*^, 5% CO_2_, 37°C

*Streptococcus sanguinis* ATCC 1.2497 Center for Microbial Resources, Institute of Microbiology, Beijing, China Broth Medium, 37°C

*Streptococcus salivarius* ATCC 1.2498 Center for Microbial Resources, Institute of Microbiology, Beijing, China Broth Medium, 37°C

*Streptococcus mutans* ATCC 1.2499 Center for Microbial Resources, Institute of Microbiology, Beijing, China Broth Medium, 37°C

*Streptomyces griseus* NBRC 13350 Center for Microbial Resources, Institute of Microbiology, Beijing, China PD, 28°C

*Streptomyces scabiei* CGMCC 4.1765 Center for Microbial Resources, Institute of Microbiology, Beijing, China Tryptone Soy Broth (TSB), 30°C

*Lysinibacillus fusiformis* Isolation and characterization by our own lab [sequencing 16S rDNA] Broth Medium, 37°C

*Enterococcus faecalis V583* (ATCC 700802) Gifted from Dr. Jing Yuan from Academy of Military Medical Sciences BHI, 37°C

Note: *Supplemented with 3% sheep blood in plates.
