# Supplementary material for: Adaptively evolved human oral actinomyces‐sourced defensins show therapeutic potential
Source: EMBO Mol Med. 2021 Dec 20;14(2):e14499. doi: 10.15252/emmm.202114499 (PMC8819291; doi:10.15252/emmm.202114499)
Supplement: Supplementary file 7 — Table EV5 [file EMMM-14-e14499-s011.docx]

**Table EV5.** Comparisons of the predicted immunogenicity and allergenicity between AMSIN and other peptides in the clinical trials and the drug - insulin

| Peptide | Immunogenicity* | Allergenicity** | Origin |
| --- | --- | --- | --- |
| AMSIN | 0.7589 (PAN) | PNAL | Bacterium |
| C16G2 | 0.4388 (PAN) | PAL | Bacterium |
| LL-37 | Not applicable | PAL | Human |
| p2TA (AB103) | Not applicable | PAL | Macaques |
| Omiganan (CLS001) | Not applicable | PAL | Cattle |
| Insulin | Not applicable | PAL | Human |
| AADNAKTKSFPV | 0.8359 (PAN) | PNAL | Virus |
| CDCRGDCFC | 1.4206 (PAN) | PAL | Virus |
| CKRGARSTC | 1.4769 (PAN) | PNAL | Virus |
| CPATAERPC | 1.0104 (PAN) | PNAL | Virus |
| CLEVSRKNC | 2.1919 (PAN) | PAL | Virus |
| SADSTKTTHLTL | 1.1187 (PAN) | PNAL | Virus |
| NRGTEWD | 1.6301 (PAN) | PNAL | Virus |

Note: * The immunogenicity of peptides was predicted by VaxiJen and the results are shown as probable antigen (PAN) or probable non-antigen (PNAN). The values represent ACC coefficients. ** The allergenicity of peptides was predicted by AllerTOP v. 2.0 and the results are shown as probable allergen (PAL) or probable non-allergen (PNAL). Peptide sequences were obtained from the literatures (Yang *et al*, 2017; Koo and Seo, 2019).
