# Supplementary material for: Adaptively evolved human oral actinomyces‐sourced defensins show therapeutic potential
Source: EMBO Mol Med. 2021 Dec 20;14(2):e14499. doi: 10.15252/emmm.202114499 (PMC8819291; doi:10.15252/emmm.202114499)
Supplement: Supplementary file 8 — Table EV6 [file EMMM-14-e14499-s014.docx]

**Table EV6.** Quality assessment of the total RNA extracted from *Streptococcus pneumoniae* R6

|  | OD_260/280_ | OD_260/230_ | RIN | 23S/16S |
| --- | --- | --- | --- | --- |
| *SP* R6 | 2.1 | 2.51 | 8.0 | 2.8 |
| *SP* R6 treated by AMSIN | 1.98 | 2.33 | 8.5 | 2.8 |
| *SP* R6 treated by vancomycin | 2.09 | 2.37 | 8.4 | 2.7 |

Note: The ratios of OD_260/280_ and OD_260/230_ were determined by NanoDrop and RNA Integrity Number (RIN) and 23S/16S were analyzed by Agilent 2100 Bioanalyzer.
