# Supplementary material for: Adaptively evolved human oral actinomyces‐sourced defensins show therapeutic potential
Source: EMBO Mol Med. 2021 Dec 20;14(2):e14499. doi: 10.15252/emmm.202114499 (PMC8819291; doi:10.15252/emmm.202114499)
Supplement: Supplementary file 9 — Table EV7 [file EMMM-14-e14499-s013.docx]

**Table EV7.** *P* values for Fig 3, Fig 5, and Appendix Figures S8 and S14

| Figure | Comparison | *P* value |
| --- | --- | --- |
| 3B | AMSIN vs AMSIN + HNP1&2 (*S. sanguinis*) | <0.0001 |
| 3B | AMSIN vs AMSIN + hLZc (*S. sanguinis*) | <0.0001 |
| 3B | AMSIN vs AMSIN + HNP1&2 (*S. salivarius*) | <0.0001 |
| 3B | AMSIN vs AMSIN + hLZc (*S. salivarius*) | 0.001 |
| 5A | 0 h vs 24 h | 0.072 |
| 5A | 0 h vs 48 h | 0.046 |
| 5A | H_2_O h vs Serum | 0.046 |
| 5C | 0 vs AMSIN (25 μg/mL) | 0.685 |
| 5C | 0 vs AMSIN (50 μg/mL) | 1.000 |
| 5C | 0 vs AMSIN (100 μg/mL) | 0.710 |
| 5C | 0 vs LL-37 (25 μg/mL) | 0.653 |
| 5C | 0 vs LL-37 (50 μg/mL) | 0.001 |
| 5C | 0 vs LL-37 (100 μg/mL) | 0.046 |
| 5D | 0 vs 6.6 mg/mL | 0.114 |
| 5F | 0 vs AMSIN (25 μg/mL) in FBS | 0.077 |
| 5F | 0 vs AMSIN (50 μg/mL) in FBS | 0.513 |
| 5F | 0 vs AMSIN (100 μg/mL) in FBS | 0.513 |
| 5F | 0 vs LL-37 (25 μg/mL) in FBS | 0.046 |
| 5F | 0 vs LL-37 (50 μg/mL) in FBS | 0.098 |
| 5F | 0 vs LL-37 (100 μg/mL) in FBS | 0.004 |
| 5F | 0 vs AMSIN (25 μg/mL) in Serum-Free | 0.004 |
| 5F | 0 vs AMSIN (50 μg/mL) in Serum-Free | 0.002 |
| 5F | 0 vs AMSIN (100 μg/mL) in Serum-Free | 0.046 |
| 5F | 0 vs LL-37 (25 μg/mL) in Serum-Free | 0.007 |
| 5F | 0 vs LL-37 (50 μg/mL) in Serum-Free | 0.002 |
| 5F | 0 vs LL-37 (100 μg/mL) in Serum-Free | 0.001 |
| 5F | AMSIN vs LL37 (25 μg/mL) | 0.526 |
| 5F | AMSIN vs LL37 (50 μg/mL) | 0.022 |
| 5F | AMSIN vs LL37 (100 μg/mL) | 0.001 |
| 5G | 0 vs AMSIN (25 μg/mL) in FBS | 0.513 |
| 5G | 0 vs AMSIN (50 μg/mL) in FBS | 0.480 |
| 5G | 0 vs AMSIN (100 μg/mL) in FBS | 0.040 |
| 5G | 0 vs LL-37 (25 μg/mL) in FBS | 0.744 |
| 5G | 0 vs LL-37 (50 μg/mL) in FBS | 0.002 |
| 5G | 0 vs LL-37 (100 μg/mL) in FBS | 0.003 |
| 5G | 0 vs AMSIN (25 μg/mL) in Serum-Free | 0.046 |
| 5G | 0 vs AMSIN (50 μg/mL) in Serum-Free | 0.046 |
| 5G | 0 vs AMSIN (100 μg/mL) in Serum-Free | 0.046 |
| 5G | 0 vs LL-37 (25 μg/mL) in Serum-Free | 0.046 |
| 5G | 0 vs LL-37 (50 μg/mL) in Serum-Free | 0.268 |
| 5G | 0 vs LL-37 (100 μg/mL) in Serum-Free | 0.507 |
| 5I | Saline vs AMSIN (8.35 mg/kg) | 0.002 |
| 5I | Saline vs Penicillin (8.35 mg/kg) | <0.0001 |
| 5I | Saline vs AMSIN (16.7 mg/kg) | <0.0001 |
| 5I | Saline vs Penicillin (16.7 mg/kg) | 0.002 |
| 5I | Saline vs AMSIN (33 mg/kg) | 0.002 |
| 5I | Saline vs Penicillin (30 mg/kg) | 0.002 |
| Appendix Figure S8 | AMSIN vs AMSIN + HNP1&2 (*B. megaterium*) | <0.0001 |
| Appendix Figure S8 | AMSIN vs AMSIN + hLZc (*B. megaterium*) | 0.001 |
| Appendix Figure S8 | AMSIN vs AMSIN + HNP1&2 (*B. subtilis*) | <0.0001 |
| Appendix Figure S8 | AMSIN vs AMSIN + hLZc (*B. subtilis*) | <0.0001 |
| Appendix Figure S8 | AMSIN vs AMSIN + HNP1&2 (MRCNS P1369) | 0.024 |
| Appendix Figure S8 | AMSIN vs AMSIN + hLZc (MRCNS P1369) | <0.0001 |
| Appendix Figure S8 | AMSIN vs AMSIN + HNP1&2 (MRSA P1386) | 0.037 |
| Appendix Figure S8 | AMSIN vs AMSIN + hLZc (MRSA P1386) | 0.001 |
| Appendix Figure S8 | AMSIN vs AMSIN + HNP1&2 (*S. aureus* J685) | <0.0001 |
| Appendix Figure S8 | AMSIN vs AMSIN + hLZc (*S. aureus* J685) | <0.0001 |
| Appendix Figure S8 | AMSIN vs AMSIN + HNP1&2 (*S. mutans*) | <0.0001 |
| Appendix Figure S8 | AMSIN vs AMSIN + hLZc (*S. mutans*) | 0.001 |
| Appendix Figure S14 | 0 vs 25 μM (AMSIN) | 0.089 |
| Appendix Figure S14 | 0 vs 25 μM (Meucin-18) | <0.0001 |

Note: *P* values derived from Mann-Whitney U test are shown in red and others from t-test.
