# Supplementary material for: Adaptively evolved human oral actinomyces‐sourced defensins show therapeutic potential
Source: EMBO Mol Med. 2021 Dec 20;14(2):e14499. doi: 10.15252/emmm.202114499 (PMC8819291; doi:10.15252/emmm.202114499)
Supplement: Supplementary file 18 [file EMMM-14-e14499-s012.pdf]

EMBO
Molecular Medicine

Adaptively evolved human oral actinomyces-sourced defensins show therapeutic potential

Shunyi Zhu, Bin Gao, Yoshitaka Umetsu, Steve Peigneur, Ping Li, Shinya Ohki, and Jan Tytgat
DOI: 10.15252/emmm.202114499

Corresponding author: Shunyi Zhu (Zhusy@ioz.ac.cn)

Review Timeline:

Submission Date: :	6th May 21
Editorial Decision:	9th Jun 21
Revision Received:	8th Oct 21
Editorial Decision:	3rd Nov 21
Revision Received:	8th Nov 21
Editorial Decision:	10th Nov 21
Revision Received:	29th Nov 21
Accepted:	3rd Dec 21

Editor: Lise Roth

Transaction Report:

(Note: With the exception of the correction of typographical or spelling errors that could be a source of ambiguity, letters and reports are not edited. Depending on transfer agreements, referee reports obtained elsewhere may or may not be included in this compilation. Referee reports are anonymous unless the Referee chooses to sign their reports.)

9th Jun 2021

Dear Prof. Zhu,

Thank you for the submission of your manuscript to EMBO Molecular Medicine, and please accept my apologies for the delay in getting back to you, as one referee needed more time to complete his/her review. We have now received feedback from the three reviewers who agreed to evaluate your manuscript. As you will see from the reports below, the referees acknowledge the interest of the study and are overall supporting publication of your work pending appropriate revisions.

Addressing the reviewers' concerns in full will be necessary for further considering the manuscript in our journal, and acceptance of the manuscript will entail a second round of review. EMBO Molecular Medicine encourages a single round of revision only and therefore, acceptance or rejection of the manuscript will depend on the completeness of your responses included in the next, final version of the manuscript. For this reason, and to save you from any frustrations in the end, I would strongly advise against returning an incomplete revision.

Revised manuscripts should be submitted within three months of a request for revision; they will otherwise be treated as new submissions, except under exceptional circumstances in which a short extension is obtained from the editor.

When submitting your revised manuscript, please carefully review the instructions that follow below. We perform an initial quality control of all revised manuscripts before re-review; failure to include requested items will delay the evaluation of your revision.

1) A .docx formatted version of the manuscript text (including legends for main figures, EV figures and tables). Please make sure that the changes are highlighted to be clearly visible.

2) Individual production quality figure files as .eps, .tif, .jpg (one file per figure). For guidance, download the 'Figure Guide PDF' (https://www.embopress.org/page/journal/17574684/authorguide#figureformat).

3) A .docx formatted letter INCLUDING the reviewers' reports and your detailed point-by-point responses to their comments. As part of the EMBO Press transparent editorial process, the point-by-point response is part of the Review Process File (RPF), which will be published alongside your paper.

4) A complete author checklist, which you can download from our author guidelines (https://www.embopress.org/page/journal/17574684/authorguide#submissionofrevisions). Please insert information in the checklist that is also reflected in the manuscript. The completed author checklist will also be part of the RPF.

5) It is mandatory to include a 'Data Availability' section after the Materials and Methods. Before submitting your revision, primary datasets produced in this study need to be deposited in an appropriate public database, and the accession numbers and database listed under 'Data Availability'. Please remember to provide a reviewer password if the datasets are not yet public (see https://www.embopress.org/page/journal/17574684/authorguide#dataavailability).

In case you have no data that requires deposition in a public database, please state so in this section. Note that the Data Availability Section is restricted to new primary data that are part of this study.

6) For data quantification: please specify the name of the statistical test used to generate error bars and P values, the number (n) of independent experiments (specify technical or biological replicates) underlying each data point and the test used to calculate p-values in each figure legend. The figure legends should contain a basic description of n, P and the test applied. Graphs must include a description of the bars and the error bars (s.d., s.e.m.).

7) We would also encourage you to include the source data for figure panels that show essential data. Numerical data should be provided as individual .xls or .csv files (including a tab describing the data). For blots or microscopy, uncropped images should be submitted (using a zip archive if multiple images need to be supplied for one panel). Additional information on source data and instruction on how to label the files are available at
.

8) Our journal encourages inclusion of *data citations in the reference list* to directly cite datasets that were re-used and obtained from public databases. Data citations in the article text are distinct from normal bibliographical citations and should directly link to the database records from which the data can be accessed. In the main text, data citations are formatted as follows: "Data ref: Smith et al, 2001" or "Data ref: NCBI Sequence Read Archive PRJNA342805, 2017". In the Reference list, data citations must be labeled with "[DATASET]". A data reference must provide the database name, accession number/identifiers and a resolvable link to the landing page from which the data can be accessed at the end of the reference. Further instructions are available at .

9) We replaced Supplementary Information with Expanded View (EV) Figures and Tables that are collapsible/expandable online. A maximum of 5 EV Figures can be typeset. EV Figures should be cited as 'Figure EV1, Figure EV2" etc... in the text and their respective legends should be included in the main text after the legends of regular figures.
- For the figures that you do NOT wish to display as Expanded View figures, they should be bundled together with their legends in a single PDF file called *Appendix*, which should start with a short Table of Content. Appendix figures should be referred to in the main text as: "Appendix Figure S1, Appendix Figure S2" etc.
- Additional Tables/Datasets should be labeled and referred to as Table EV1, Dataset EV1, etc. Legends have to be provided in a separate tab in case of .xls files. Alternatively, the legend can be supplied as a separate text file (README) and zipped together with the Table/Dataset file.
See detailed instructions here:
.

10) For more information: There is space at the end of each article to list relevant web links for further consultation by our readers. Could you identify some relevant ones and provide such information as well? Some examples are patient associations, relevant databases, OMIM/proteins/genes links, author's websites, etc...

11) Every published paper now includes a 'Synopsis' to further enhance discoverability. Synopses are displayed on the journal webpage and are freely accessible to all readers. They include a short stand first (maximum of 300 characters, including space) as well as 2-5 one-sentences bullet points that summarizes the paper. Please write the bullet points to summarize the key NEW findings. They should be designed to be complementary to the abstract - i.e. not repeat the same text. We encourage inclusion of key acronyms and quantitative information (maximum of 30 words / bullet point). Please use the passive voice. Please attach these in a separate file or send them by email, we will incorporate them accordingly.

Please also suggest a striking image or visual abstract to illustrate your article as a PNG file 550 px wide x 300-600 px high.

EMBO Molecular Medicine has a "scooping protection" policy, whereby similar findings that are published by others during review or revision are not a criterion for rejection. Should you decide to submit a revised version, I do ask that you get in touch after three months if you have not completed it, to update us on the status.

I look forward to seeing a revised form of your manuscript as soon as possible. Use this link to login to the manuscript system and submit your revision: https://embomolmed.msubmit.net/cgi-bin/main.plex

Yours sincerely,

Lise Roth

Lise Roth, Ph.D
Editor
EMBO Molecular Medicine

***** Reviewer's comments *****

Referee #1 (Comments on Novelty/Model System for Author):

Im surprised all tests were t tests and no requirement for ANOVA?

Referee #1 (Remarks for Author):

Discovering new antimicrobials is essential for human health. This paper seeks to determine whether oral cavity, bacterially derived antimicrobials may be effective against pathogens when delivered systemically. The authors identify by genome search a cluster of ancient invertebrate-type defensins (AITDs) in actinobacteria and myxobacteria that are present in human mouth and ruminants. These are evolving through positive selection. The authors show that one synthetically made actinomyces peptide (AMSIN) is of similar structure to defensins and with seven cysteines oxidises to a dimer. Finally they show that it is able to kill a variety of bacteria of both Gram +ve and -ve strain and reduces the lethality of systemic MRSA and infection level of mice given airway delivered Strep pneumoniae.
A few comments/questions are
Was charge a feature of the peptides? Useful to have in figure 1.

How much serum was present in figure 5A and molarity is more useful than nM/well?
Did denaturation of the disulfide bonds or a linear molecule reduce effectiveness?
The infection model very interesting. As peptide delivery is distant and/or delayed by 1 hour one assumes its direct killing causing the benefit but cytokine response, weight loss and cellular changes in BAL for example would be useful if you have the data.
A very interesting paper.

Referee #2 (Remarks for Author):

Summarise the main findings of the study:
This article majorly focuses on "Adaptively Evolved Human Oral Actinomyces-Sourced Defensins Show Therapeutic Potential". Human oral bacteria are a possible new source of peptide antibiotics by the author. This paper explores the use of AMPs from unicellular, prokaryotic organisms as a replacement for AMPs derived from humans and other vertebrates. To prove this, the author used defensins to take advantage of the structural diversity and therapeutic potential of AMPs derived from actinobacteria and myxobacteria.

Highlight the limitations and strengths;
Along with the manuscript, extensive experimental work has been carried out. The outcomes are both intriguing and novel. The experimental design and statistics used are both satisfactory. The discussion is organised around the findings and includes the most relevant literature. Significant changes are required before the acceptance of the manuscript for the journal.

Comment on the methods, results, and data interpretation;
All results are exciting and innovative. The entire manuscript comprises of five noteworthy findings, with the experimental design and statistics in "Structural diversity of bacterial defensins, Antibacterial activity, and Synergy of AMSIN with Oral AMPs, AMSIN is an inhibitor of bacterial cell wall biosynthesis" being appropriate. There is a lack of evidence to support the concept in "Discovery and evolution of bacterial defensins, and the therapeutic potential of AMSIN," which necessitates significant revision.

Major points:
• An inhibition zone-based assay, hemolysis by AMSIN, the effect of AMSIN on human ion channels expressed in the CNS and heart, the cytotoxic effects of AMSIN, and treatment of pneumococcal pneumonia in vivo, are among the assays performed by the author in order to determine the therapeutic potential of AMSIN. All of them are well-done and interpreted. However, despite the author's claims that this antibacterial peptide is a systemic drug. How does the author justify the antibacterial peptide's safety in human use?

• They did not conduct any assays or experiments to demonstrate the immunogenicity and allergenicity of AMSIN because "systemic drugs work by entering the circulatory system and affecting the whole body". This peptide can activate an immune response in the circulatory system, where all immune cells are present, despite its evolutionary distance from humans.

• I recommend performing assays dependent on AMSIN's immunogenicity and allergenicity, at the very least, in mice. And also, using various online bioinformatics resources such as VaxiJen 2.0, AllerTOP, EpiTOP, and any others, please predict the antigenicity-immunogenicity of your peptide sequence in humans.

• I also recommend that authors perform a yeast two hybridisation assay to show whether HNP1 & 2 and hLZc physically interact with AMSIN. It will reveal whether the AMSIN peptide molecules' functions are dependent or independent (in Figure 3.B.). It will also clarify whether or not there are any physical interactions with HNP1 & 2 and hLZc and provide evidence for combining AMSIN treatment with HNP1 & 2 and/or hLZc.

• The author compares fewer species (in Figure 1D); I have curiosity on what basis did the author choose selective invertebrates for multiple sequence alignment (MSA), or did they handpick these eukaryotes (multicellular AITDs)? I suggest providing the decisive reason for their selection in the list.

• In Fig EV4, the author should show "the MSA of AITDs from Actinobacteria (A) and Myxobacteria" as a percentage similarity and conservation index for each AMPs.

• Why did the author not use human defensins for MSA and phylogenetic tree construction in silico (in Figure 1)?

• The authors have suggested the nonspecific cytotoxicity of AMPs. The liposomes can also be used to encapsulate the AMPs that masks their unwanted effects during systemic delivery.
• The type of interaction of AMPs with the bacterial cell membrane and the mammalian cell membrane is different. The outer layer of bacterial cell membrane comprised of mostly lipids with negatively charged groups whereas the outer leaflet of mammalian lipid membrane is neutrally charged with the presence of zwitterionic phospholipids and cholesterol. Therefore, AMPs have weaker interaction with the mammalian cell membrane and more vital electrostatic interaction with the bacterial cell

membrane. What could be the explanation provided by the authors about the nonspecific cytotoxicity of AMPs? It is not clearly statement.

• The authors have mentioned that human and vertebrates derived. AMPs are ineffective due to their immunomodulatory properties. However, in the present study, the authors have used the AMSIN, which is also the human-derived AMP from actinomyces colonised in the human cavity, how the authors explained the non-immunomodulatory nature of this AMP during the systemic delivery in in vivo system.

• Why only actinobacteria and myxobacteria selected?

• Why only mouse peritoneal model 20 mg dose selected, it should be 10, 15,20?

• Is the dose for heart and lung safety check selected according to human equivalent dose?

• How this study directly related to oral health?

• Time-kill test in co-cultures for the antimicrobial activity should be done.

• Hematological analysis and enzymatic assay for liver and kidney test for the safety assessment should also be done.

• For cytotoxicity: Neutral red can also do? Other experiments related to streptococcus/staphylococcus infection in cell line (heart or liver) and protective effects of AMP can be studied.

• Include the animal experimental and In vitro schematic representation in the Material and method section.

• To explore potential defensin-like peptides in bacteria, how authors selected the eukaryotic defensins as queries?

• While determining lethal concentrations of AMSIN against various bacteria, is there any criteria to choose only these strains or how to decide which strains need to check?

• As stated, HNP1, contains an extra N terminal Ala relative to HNP2. Does it create any effect on the antibacterial activity?

• Why the main emphasis only on cysteine-based structural variations (in structural diversity of bacterial defensins)?

• Actinomycesin specifically inhibits the bacterial peptidoglycan synthesis and shows no toxicity on a panel of human ion channels relevant in the heart and the central nervous system, and mammalian cells. Why only heart and CNS were chosen?
• To check the therapeutic effect of the AMSIN gene, why only cell viability was checked? Other parameters can also be considered, like- the cell line can be Staphylococcus and Streptococcus in vivo and in vitro, and the effect of the AMSIN gene can be checked.

• While determining the in vivo anti-infective potency of AMSIN, why the two mouse models of systemic infections- the mouse pneumonia model and the peritonitis model were chosen?

• AMSIN was introduced intravenously (i.v.) one hour post-infection 20 mg per kg. This treatment continued for five days (once each day). How this concentration and time - period was determined?

• While assessing the effect of AMSIN on human ion channels expressed in the CNS and heart, the peptide dose used was 100 μM. Why 100 μM?

Minor correction require;
• The author used vague scientific terminology in their writing; they should have been more precise in their writing, as they often used words like antibiotics and AMSIN.

• In the material method section specify the animal species and ethical number.

• The author emphasises that "they had described 48 such peptides from two microbial subgroups (actinobacteria and myxobacteria belonging to deltaproteobacteria)," but they do not specify which isoform of AMSIN they are operating on throughout the manuscript. The author should state this.

• An author suggested submitting an English proficiency certificate for the concerned manuscript before final submission because the English language is not of good quality, and there is also wordiness.

• The author has written many too many long sentences, making this explanation challenging to comprehend. Below is a list of sentences that must be changed;

o Actinomycesin and its homologs from actinobacteria and myxobacteria share similarity to a group of ancient invertebrate-type defensins from fungi and invertebrates, and those from actinomyces colonised in the human oral cavity and ruminant rumen and dental plaque are evolving under positive selection, supporting their role implicated in adaptation to these complex multispecies bacterial communities.

o Defensins from actinomyces colonised in the human oral cavity and ruminant rumen and dental plaque other than from fungi and animals are evolving under positive Darwinian selection at their molecular surface, in favour of their role in ecological adaptation to the complex oral and rumen multispecies communities.

o Analysis of the intracellular pool of cell-wall precursors by means of reversed-phase high-performance liquid chromatography (RP-HPLC) combined with mass spectrometry revealed the accumulation of the soluble molecule UDP-MurNAc-pentapeptide (abbreviated as UMP) in AMSIN- or vancomycin-treated cells (Fig 4D), suggesting that the peptide may work in a similar manner to the inhibitors of cell-wall synthesis.

o We found that AMSIN exhibited a kinetic behaviour highly similar to vancomycin, a typical bacterial cell wall synthesis inhibitor, but differed from rifampicin that targets bacterial DNA-dependent RNA polymerase to inhibit RNA synthesis and kanamycin that works by interfering with protein synthesis (Fig 4A).

o UMP contains the elements responsible for direct interaction of Lipid II with plectasin (i.e. the pyrophosphate group and the γ-Glu in the pentapeptide) (Schneider et al 2010) or vancomycin (i.e. the D-Ala-D-Ala 20 motif) (Hernout et al, 2007) (Fig 4E; Fig EV13), thus allowing to investigate a possible interaction between AMSIN and Lipid II by using UMP as an alternative.

o As verified by AMSIN whose high efficacy and low toxicity as well as serum stability, together with a bacterial origin and potential complex microflora-driven adaptive evolution, all indicate that actinomycetes-derived defensins are promising Lipid II-bound antibiotic leads with greater therapeutic potential than their counterparts in eukaryotes.

Referee #3 (Comments on Novelty/Model System for Author):

The authors used both in silico, in vitro and in vivo system to characterize the functions of the molecule studied.
Methods used are adequate however the use of T-test as statistical test should be justified.
The topic of the study is quiet novel and with strong medical impact.

Referee #3 (Remarks for Author):

EMM-2021-14499
The present study reports and characterized a human oral actinomyces-derived defensin (actinomycesin) with therapeutic potential. After in silico analyses of microbial-derived defensins, the authors biologically characterized actinomycesin. The authors demonstrated:
sing NOD mouse model and streptozotocin-induced model in C57BL/6 mice, the authors aim to demonstrate that:
-Actinomycesin shows a lytic effect on various pathogenic bacteria.
-Lytic effect synergizes with oral human antimicrobial peptides (alpha-defensin 1&2 and lysozyme C).
-Actinomycesin acts on bacteria by inhibiting bacterial cell wall biosynthesis.
-Actinomycesin has anti-infective potency in mouse models of infection (pneumonia and peritonitis).
The present study provides important insights to the current knowledge but the authors need to provide additional data to strengthen their conclusion.
The authors need to further characterize the potential toxicity of actinomycesin in vivo via different routes of administration in animal models.
Regarding the growing knowledge regarding the immunomodulatory roles of defensins, this aspect need to be investigate almost in vitro (chemotactic activity, pro- or anti-inflammatory activity...).

The number of samples analyzed and the number of experiments need to be mentioned in the figure legends. Indeed, this is mandatory to determine if the T-test used by the authors is adapted to analyze the data. T-test requires 20 independent values per group. If it is not the case, a non-parametric Mann-Whitney test must be used.

EMM-2021-14499

Adaptively Evolved Human Oral Actinomyces-Sourced Defensins Show Therapeutic Potential

Response to *EMBO Molecular Medicine* Reviewers Comments

Referee #1

Thank you very much for your positive comments and constructive suggestions on our work that are very valuable for improving our manuscript as well as guiding our future research. Please see below our point-to-point response to your issues:

Point 1. *Comments on Novelty/Model System for Author: I'm surprised all tests were t tests and no requirement for ANOVA?*

--In this revised version, statistical significance between two groups of means was determined by Student's t-test for the data that accord with normal distribution and homogeneity of variance, as reported in other studies (e.g. Monteil *et al.* 2020. *Cell* 181: 905-913: "Statistics: Statistical analysi was conducted using GraphPad Prism 8 (GraphPad) and significance was determined by Student's test"). For the data that did not meet any one of the two conditions, we used the nonparametric Mann-Whitney U test to compare the difference between two groups of means (Hoffman 2015. *Biostatistics for Medical and Biomedical Practitioners*, Academic Press, pp. 337-362). Although one-way ANOVA is also suitable for comparison of two-samples data to determine their significance, the sample sizes analyzed here are smaller than 30 (i.e. $10 \geq n \geq 3$) and do not meet the requirement of ANOVA ($n>30$). Thus, we chose t-test or Mann-Whitney U test, a version of ANOVA restricted to two groups and a smaller sample size (<30), for our statistical analysis (please see "Statistics" in the Materials and methods section).

Remarks for Author: Discovering new antimicrobials is essential for human health. This paper seeks to determine whether oral cavity, bacterially derived antimicrobials may be effective against pathogens when delivered systemically. The authors identify by genome search a cluster of ancient invertebrate-type defensins (AITDs) in actinobacteria and myxobacteria that are present in human mouth and ruminants. These are evolving through

positive selection. The authors show that one synthetically made actinomyces peptide (AMSIN) is of similar structure to defensins and with seven cysteines oxidises to a dimer. Finally they show that it is able to kill a variety of bacteria of both Gram +ve and -ve strain and reduces the lethality of systemic MRSA and infection level of mice given airway delivered Strep pneumoniae. A few comments/questions are:

Point 2. *Was charge a feature of the peptides? Useful to have in figure 1.*

--We have added the charge information, described as net charge and isoelectric point (pI), for each peptide (please see Fig. 1 in the revised version).

Point 3. How much serum was present in figure 5A and molarity is more useful than nM/well?

--The serum used was fresh normal mouse serum without dilution. In this assay, 2 μl of 100 μM AMSIN was added to each well of bacterial plates, which equals 0.2 nmol/well of peptide dose. Because in inhibition-zone assays the calculation of lethal concentration (C_L) of a peptide requires the amount of sample applied in the well (in nmol), we thus used a peptide dose instead of using peptide concentration (please see "Antibacterial Assays" in the Materials and methods section).

Point 4. Did denaturation of the disulfide bonds or a linear molecule reduce effectiveness?

--We have compared the antibacterial activity between the linear (reduced) AMSIN without disulfide bonds formed and the cyclic one (oxidized) with the disulfide bonds formed. The result showed that this peptide in its reduced form still exhibited activity comparable to the cyclic version (please see Appendix Figure S6 and the associated text on page 8: lines 241-245).

Point 5. The infection model very interesting. As peptide delivery is distant and/or delayed by 1 hour one assumes its direct killing causing the benefit but cytokine response, weight loss and cellular changes in BAL for example would be useful if you have the data. A very interesting paper.

--In this revised version, we have added new data regarding the cytokine response by assaying the effect of AMSIN on IL-8 (interleukin-8) release by A549 cells with an ELISA (please see Figure 5F and the associated text on pages 10 and 11: lines 331-340). When

evaluating the *in vivo* therapeutic efficacy of AMSIN, we referenced two related papers reporting the discovery of new peptide antibiotics (Mygind *et al.* 2005. *Nature* 437: 975-980; Ling *et al*. 2015. *Nature* 517: 455-459) without recording the data related to weight loss and cellular changes. However, your instructive suggestions are valuable for our future research to exploit AMSIN as a clinical drug.

Referee #2

Thank you very much for your positive comments and constructive suggestions on our work that are very valuable for improving our manuscript as well as guiding our future research. Please see below our point-to-point response to your issues:

Point 1. *Remarks for Author:*

Summarise the main findings of the study: This article majorly focuses on "Adaptively Evolved Human Oral Actinomyces-Sourced Defensins Show Therapeutic Potential". Human oral bacteria are a possible new source of peptide antibiotics by the author. This paper explores the use of AMPs from unicellular, prokaryotic organisms as a replacement for AMPs derived from humans and other vertebrates. To prove this, the author used defensins to take advantage of the structural diversity and therapeutic potential of AMPs derived from actinobacteria and myxobacteria.

Highlight the limitations and strengths; Along with the manuscript, extensive experimental work has been carried out. The outcomes are both intriguing and novel. The experimental design and statistics used are both satisfactory. The discussion is organised around the findings and includes the most relevant literature. Significant changes are required before the acceptance of the manuscript for the journal.

Comment on the methods, results, and data interpretation; All results are exciting and innovative. The entire manuscript comprises of five noteworthy findings, with the experimental design and statistics in "Structural diversity of bacterial defensins, Antibacterial activity, and Synergy of AMSIN with Oral AMPs, AMSIN is an inhibitor of bacterial cell wall biosynthesis" being appropriate. There is a lack of evidence to support the concept in "Discovery and evolution of bacterial defensins, and the therapeutic potential of AMSIN," which necessitates significant revision.

--To address your comments, we have revised these two sections. For “Discovery and evolution of bacterial defensins”, we have provided the surveyed database information for

TBLASTN search, which contained 39 microbial subgroups with four different levels of genome assembly (please see page 4: lines 133-135). We also provided the detail about how the eukaryotic defensins were chosen as queries (please see pages 14 and 15: lines 474-480). To emphasize our finding in the evolution of bacterial defensins, we have removed the original Expanded View Table EV7. “Maximum likelihood estimates of parameters and sites inferred to be under positive selection in the actinomyces-sourced defensins” to the body of the work as Table 1 to present significant signatures of natural selection. To strength our opinion, we revised and improved our discussion about the potential of bacterial defensins as anti-infective drugs in the context of natural selection (positive selection) imposed by the complex oral and rumen microflora (please see page 6: lines 179-187).

For the section “the therapeutic potential of AMSIN”, we have added new experimental data to strengthen the evidence for AMSIN as a systemic drug. We showed that AMSIN displayed no detectable immunomodulatory activity in the fetal bovine serum (FBS)-containing medium and weak immunomodulatory activity in the absence of the serum via using an ELISA to evaluate its ability in stimulating IL-8 release. Our data indicate that AMSIN has a relatively high stability in both undiluted FBS and human serums without detectable immunogenicity (please see Figure 5 and Appendix Figure S13 and the associated text on pages 10 and 11: 316-320; lines 331-343). Therefore, of all of these properties relevant to therapeutic efficacy, AMSIN is superior to some eukaryotic AMPs (e.g. human LL-37 and various defensins). The latter often exhibits potent mammalian cytotoxicity with strong immunomodulatory activity, serum instability and inactivation (Panyutich *et al*. 1995. A*m J Respir Cell Mol Biol* 12: 351-357; Johansson *et al*. 1998. *J Biol Chem* 273: 3718-3724; Scott *et al*. 2002. *J Immunol* 169: 3883-3891; Hara *et al*. 2008. *Biochem Biophys Res Commun* 374: 709-713; Zhu and Gao 2017. *Heredity* (Edinb). 118: 453-465; Kudryashova *et al*. 2021. *J Mol Biol* 167225. doi: 10.1016/j.jmb.2021.167225).

Major points:

Point 2. *An inhibition zone-based assay, hemolysis by AMSIN, the effect of AMSIN on human ion channels expressed in the CNS and heart, the cytotoxic effects of AMSIN, and treatment of pneumococcal pneumonia in vivo, are among the assays performed by the author in order to determine the therapeutic potential of AMSIN. All of them are well-done and interpreted. However, despite the author's claims that this antibacterial peptide is a systemic drug. How does the author justify the antibacterial peptide's safety in human use?*

--Our current study is still in the laboratory stage without data regarding the safety of this peptide in human use. However, the use of mice as a model system for investigation of the therapeutic potential of newfound antibiotics has been a standard method extensively used, as the studies reported previously (Mygind *et al.* 2005. *Nature* 437: 9759-80; Ling *et al.* 2015. *Nature* 517: 455-459). The rationality behind this is the relatively close evolutionary relationship between humans and mice. The safety of AMSIN confirmed in mice suggests that it has a clinical translational prospect for further investigations of its safety in human use. In addition, the lack of toxicity on human hERG K^+ channel reported here points outs the safety of AMSIN to human heart.

Point 3. *They did not conduct any assays or experiments to demonstrate the immunogenicity and allergenicity of AMSIN because "systemic drugs work by entering the circulatory system and affecting the whole body". This peptide can activate an immune response in the circulatory system, where all immune cells are present, despite its evolutionary distance from humans. I recommend performing assays dependent on AMSIN's immunogenicity and allergenicity, at the very least, in mice. And also, using various online bioinformatics resources such as VaxiJen 2.0, AllerTOP, EpiTOP, and any others, please predict the antigenicity-immunogenicity of your peptide sequence in humans.*

--According to your suggestions, we have evaluated the immunogenicity of AMSIN by using the surface plasmon resonance (SPR) technique (Hermeling *et al*. 2005. *Pharm Res* 22: 1997-2006; Real-Fernández *et al*. 2015. *Anal Bioanal Chem* 407: 7477-7485). SPR experiments detected no obvious anti-drug antibody in the AMSIN-treated mouse serum, as identified by very weak response units (RUs) when the serum taken from AMSIN-treated mice flowed through the AMSIN-immobilized chip surface, suggesting that as a peptide-based drug AMSIN was less immunogenic than other biologic therapeutics. Thank you for your suggestion, we added prediction of the immunogenicity of AMSIN with VaxiJen and its allergenicity by AllerTOP v. 2.0, and compared them with those of some currently developed peptide drugs. AMSIN has a predicted immunogenicity (score 0.76) smaller than the peptides (scores: 0.84-2.19) and is a probable non-allergen. The prediction is overall consistent with the experimental data (please see Figure 5G and the associated text on page 11: lines 340-348; Table EV5).

Point 4. *I also recommend that authors perform a yeast two hybridisation assay to show*

whether HNP1 & 2 and hLZc physically interact with AMSIN. It will reveal whether the AMSIN peptide molecules' functions are dependent or independent (in Figure 3.B.). It will also clarify whether or not there are any physical interactions with HNP1 & 2 and hLZc and provide evidence for combining AMSIN treatment with HNP1 & 2 and/or hLZc.

--To study the potential interactions between AMSIN and HNP1&2 or hLZc, we used bio-affinity electrospray ionization mass spectrometry (ESI-MS) instead of a yeast two-hybrid assay. ESI-MS is an efficient technique for the evaluation of solution-phase non-covalent interactions, e.g. lysozyme and ginsenosides (McCullough and Gaskell 2009. *Comb Chem High Throughput Screen* 12: 203-211; Tang *et al*, 2015. *Rapid Commun Mass Spectrom* 29: 2031-2038). Using ESI-MS, we confirmed no complex formation between AMSIN and HNP1&2 or hLZc, suggesting that their antibacterial synergy may be independent on their physical interactions (please see Appendix Figure S9 and the associated text on page 8: lines 258-261). On the basis of this result combined with the target identification, we proposed an explanation for the synergy: (1) Partial hydrolysis of the β-1,4 glucosidic linkages of the cell wall peptidoglycan by a small amount of lysozyme (Ragland and Criss 2017) could prompt easier access of AMSIN to the membrane-anchored Lipid II. (2) Binding of HNP1&2 to Lipid II might induce a conformation that more facilitates AMSIN's binding. This is in line with the findings that HNP1 and plectasin bind to two separate regions on Lipid II (please see pages 9-10: lines 304-310).

Point 5. The author compares fewer species (in Figure 1D); I have curiosity on what basis did the author choose selective invertebrates for multiple sequence alignment (MSA), or did they handpick these eukaryotes (multicellular AITDs)? I suggest providing the decisive reason for their selection in the list.

--In Figure 1D, we chose the top BLASTP hits obtained by AMSIN as query to search against the GenBank non-redundant protein sequences database (www.ncbi.nlm.nih.gov) for alignment and comparison with AMSIN (please see the legend of Figure 1D).

Point 6. In Fig EV4, the author should show "the MSA of AITDs from Actinobacteria (A) and Myxobacteria" as a percentage similarity and conservation index for each AMPs. • Why did the author not use human defensins for MSA and phylogenetic tree construction in silico (in Figure 1)?

--We have provided such information for the alignment (initial Fig EV4 now changed to Appendix Figure S2), in which both percentages of identity and similarity were calculated with GeneDoc (https://github.com/karlnicholas/GeneDoc). That we excluded human defensins in the MSA and phylogenetic tree presented in Figure 1 was because these defensins and AITDs belong to different structural classes, namely *trans*- and *cis*- with different disulfide bridge connectivity patterns and loop sizes (Shafee *et al.* 2016. *Mol Biol Evol* 33: 2345-2356), all these hampering a reliable MSA and thus a phylogenetic tree construction.

Point 7. The authors have suggested the nonspecific cytotoxicity of AMPs. The liposomes can also be used to encapsulate the AMPs that masks their unwanted effects during systemic delivery.

--We agree with your comment on the postformulation of AMPs in reducing their nonspecific cytotoxicity. We added one sentence in the revised version "Encapsulation of AMSIN into liposomes (Pan *et al,* 2012) or PEGylation (Veronese and Mero, 2008) may further improve its therapeutic efficacy through eliminating the minor unwanted properties." (please see page 13: lines 410-412)

Point 8. The type of interaction of AMPs with the bacterial cell membrane and the mammalian cell membrane is different. The outer layer of bacterial cell membrane comprised of mostly lipids with negatively charged groups whereas the outer leaflet of mammalian lipid membrane is neutrally charged with the presence of zwitterionic phospholipids and cholesterol. Therefore, AMPs have weaker interaction with the mammalian cell membrane and more vital electrostatic interaction with the bacterial cell membrane. What could be the explanation provided by the authors about the nonspecific cytotoxicity of AMPs? It is not clearly statement.

--We agree with your comments about the differences between bacterial and mammalian membrane components. Although there is such a difference, many eukaryotic AMPs are found to exhibit a nonspecific toxicity on both of them though the molecular bases remains poorly understood. For example, melittin, marmelittin, Meucin-13, MeuFSPL-1, MeuFSPL-2, Meucin-18, Meucin-22, Marcin-22, Meucin-49, etc. All these peptides can create a lytic effect on mammalian erythrocytes (e.g. Gao *et al.* 2018. *Amino Acids* 50: 1025-1043; Gao *et al.* 2009. *FASEB J* 23: 1230-1245; Gao and Zhu 2018. *Front Microbiol* 9: 320), as shown

here for Meucin-18 (please see Fig 5B). This phenomenon was recently reviewed as: “In spite of their generally favorable safety profile, AMPs can also, in parallel with microbial membrane affinity, interact and disassemble the membrane of eukaryotic cells, particularly erythrocytes, leading to cytotoxicity (especially hemolysis). This represents a challenge” (Mahlapuu *et al.* 2020. *Crit Rev Biotechnol* 40: 978-992). To address the reviewer comments, we have added several related literature (please see page 3, lines 76-79).

Point 9. The authors have mentioned that human and vertebrates derived. AMPs are ineffective due to their immunomodulatory properties. However, in the present study, the authors have used the AMSIN, which is also the human-derived AMP from actinomyces colonised in the human cavity, how the authors explained the non-immunomodulatory nature of this AMP during the systemic delivery in in vivo system.

--In the revised version, we compared the immunomodulatory activity of AMSIN with that of LL-37, a human cathelicidin antimicrobial peptide LL37 with immunomodulatory properties on various human cells (Scott *et al*. 2002. *J Immunol* 169: 3883-3891; Burton and Steel 2009. *Nat Prod Rep* 26: 1572-1584; Alford *et al.* 2020. *Front Microbiol* 11: 1902) through assaying its effect on IL-8 (interleukin-8) release by A549 cells, a human alveolar type II cell line. We found that remarkably different from LL-37, AMSIN did not induce an elevated IL-8 release at a concentration range (25-100 μg/mL) when the cells were cultured in the FBS-containing medium. Without the serum, it showed some activity but the activity was generally weaker than that of LL-37. This provides further support for our opinion proposed in this work.

From an evolutionary viewpoint, natural selection acting on the molecular surface of the bacterial defensins may be a consequence of species competition for the actinomycetes adapting to the complex oral and rumen microflora. On the contrary, natural selection of mammalian LL-37 is associated with the evolutionary variability of their endogenous receptors mediating the immunomodulatory functions rather than bactericidal activity (Zhu and Gao 2017. *Heredity* (Edinb). 118: 453-465). Therefore, though AMSIN and LL-37 are derived from the human body, the selective pressure driving their evolution is obviously different. This could explain their difference in the immunomodulatory activity and against consolidates the advantages of these bacterial defensins as anti-infective drugs relative to human AMPs (please see Figure 5F and the associated text on pages 10-11: lines 331-340).

Point 10. Why only actinobacteria and myxobacteria selected?

--We conducted gene mining of bacterial defensins by surveying the GenBank Microbial Genomes database by TBLAST, which contained 39 different microbial subgroups. We found that only the genomes from species belonging to actinobacteria and myxobacteria contain genes encoding defensin-like peptides (please see Dataset EV1; page 4: lines 133-135).

Point 11. Why only mouse peritoneal model 20 mg dose selected, it should be 10, 15, 20?

--We selected the doses of AMSIN in the mouse peritoneal model by reference to the literature ("Treatment with plectasin subcutaneously at 10 mg per kg was initiated one hour after inoculation.") (Mygind *et al*. 2005. *Nature* 437: 975-980) with slight modification, in which the first-day dose was 20 mg per kg once (prim.vic.No2) and then the second to the fifth day at 10 mg per kg once daily. AMSIN can achieve a maximal therapeutic efficacy in this dose regiment initially determined by a pre-experiment.

Point 12. Is the dose for heart and lung safety check selected according to human equivalent dose?

--Yes. Based on our estimation, the dose 20 mg/kg of AMSIN used here corresponds to about 74 μM in mouse blood and 10 mg/kg corresponds to 37 μM. Our *in vitro* electrophysiological experiments confirmed that even 100 μM AMSIN was not toxic to human heart K^+ channel (hERG), a target for evaluating anticipated cardiovascular toxicity and strongly recommended by U.S. Food and Drug Administration (FDA) and European Medicines Agency (EMA) (Garrido *et al.* 2020. *Eur J Med Chem* 195: 112290). This indicates that our dose regiment in the mouse peritoneal model should be safe to mouse and human hearts. Given that the *in vivo* experiments showed therapeutic potential on mice, we speculated that AMSIN would be safe to human lung. However, this still needs further investigation in the future.

Point 13. *How this study directly related to oral health?*

--Of the three AMSIN-sensitive oral bacteria tested here, *S. mutans* is a primary colonizer of teeth and also a major cariogenic pathogen causing human oral disease (dental caries), especially in the case of disease onset(Krzyściak *et al*. 2014. *Eur J Clin Microbiol*

Infect Dis 33: 499-515; Lemos *et al*. 2019. *Microbiol Spectr* 7: 10.1128/microbiolspec.GPP3-0051-2018). Based on its potent antibacterial activity and synergy with oral AMPs, we can infer that AMSIN may play a direct role in oral health by inhibiting the overgrowth of *S. mutans* on the tooth surface in the normal physiological condition and thus is likely developed into a local drug for human dental caries. Besides as a systimic drug for treating infections of *S. pneumoniae* and drug-resistant *S. aureus*, AMSIN might be a valuable lead for developing novel drugs for treating infective endocarditis elicited by the opportunistic oral pathogen *Streptococcus sanguinis* should the bacteria enter the bloodstream and colonize a damaged heart valve (please see pages 12-13: lines 418-428).

Point 14. Time-kill test in co-cultures for the antimicrobial activity should be done.

--We have done the experiment to determine the *in vitro* killing kinetics of AMSIN on *Streptococcus mutans* (*SM*) (please see Figure 4B and the associated text on pages 8-9: lines 269-273).

Point 15. Hematological analysis and enzymatic assay for liver and kidney test for the safety assessment should also be done.

--Thank you for your valuable comments on the safety assessment of AMSIN. In this study, we have provided evidence in favor of the safety of AMSIN in two levels. (1) *In vitro*. AMSIN exhibits (a) no hemolysis, cytotoxicity and immunomodulatory activity on mammalian cells at a concentration far beyond its lethal concentrations for bacterial killing; (b) no DNA binding ability; (c) no toxicity on multiple ion channels relevant in human heart and the central nervous system (CNS). (2) *In vivo*. AMSIN was highly efficacious in mice infected with *Streptococcus pneumoniae* and was capable of curing mice of experimental peritoneal infection caused by methicillin-resistant *Staphylococcus aureus*. Because the focus of this work was: (1) to discover of bacterial counterparts of eukaryotic AMPs; (2) to study their evolution and structural diversity; (3) to characterize their interacting targets; and (4) to evaluate their therapeutic potential, a detailed assessment of its toxicity on various organs could be beyond the scope of this study. However, your instructive suggestions are valuable to our next-step research to develop AMSIN into a clinical drug. Thank you for your understanding.

Point 16. *For cytotoxicity: Neutral red can also do? Other experiments related to streptococcus/staphylococcus infection in cell line (heart or liver) and protective effects of AMP can be studied.*

--Please see our response to Point 15.

Point 17. *Include the animal experimental and in vitro schematic representation in the Material and method section.*

--We have included the contents (please see Appendix Figure S15. "Schematic representation of the *in vitro* and animal experiments.").

Point 18. To explore potential defensin-like peptides in bacteria, how authors selected the eukaryotic defensins as queries?

--Firstly, we downloaded all eukaryotic AMP sequences deposited in the APD3 database (https://aps.unmc.edu/) and then performed cluster analysis by the ClustalX program (http://www.clustal.org/). The clusters with sequences annotated as "defensin" were selected. To reduce the database search workload, we used CD-HIT to remove redundant sequences with a cutoff of 0.5 (http://weizhongli-lab.org/cd-hit/). The collected sequences included *cis*- and *trans*-classes (Shafee *et al.* 2016. *Mol Biol Evol* 33: 2345-2356). Using these sequences as queries, we conducted an iterative TBLASTN search of the microbial genomes database (please see "*Gene discovery*" in the Materials and methods section).

Point 19. While determining lethal concentrations of AMSIN against various bacteria, is there any criteria to choose only these strains or how to decide which strains need to check?

--Based on the origin of AMSIN from an oral bacterium, we firstly assayed its antibacterial activity on some common oral bacteria (e.g. oral *Streptococcus* bacteria including *S. mutans*, *S. salivarius* and *S. sanguinis*) as test strains. On this basis, we extended our assay to some clinically relevant and drug-resistant strains in human and animal infections (e.g. *Staphylococcus aureus Staphylococcus epidermidis, Streptococcus pneumoniae, and Enterococcus faecalis*). We added one sentence to describe the choice of the bacterial strains (please see page 7: lines 229-231).

Point 20. *As stated, HNP1, contains an extra N terminal Ala relative to HNP2. Does it create any effect on the antibacterial activity?*

--The study from Ericksen *et al*. has shown that the extra N-terminal Ala does not create any obvious effect on the antibacterial activity of HNP1 compared with that of HNP2, as indicated by LD_{50} (median lethal dose) against multiple bacterial strains (Ericksen *et al*. 2005. *Antimicrob Agents Chemother* 49: 269-275). This is also the case found in the LL-37 processing. Relative to LL-37, ALL-38 (an enzymatically processed version from a proLL-37 in human seminal plasma) also contains an extra N-terminal Ala and its antimicrobial activity also equaling to that of LL-37 (Sørensen *et al.* 2003. *J Biol Chem* 278: 28540-28546). We added the related literature (please see page 8: lines 250-251).

Point 21. *Why the main emphasis only on cysteine-based structural variations (in structural diversity of bacterial defensins)?*

--Bacterial defensins belong to the groups of AITDs, all members of which contain six evolutionarily conserved cysteine residues forming three intramolecular disulfide bridges to stabilize the well-defined CSαβ fold. Different from noncysteine mutations or loop insertion/deletion (indel) mutations that normally does not alter the structural features of a defensin, introducing single or a pair of cysteines often does. For example, the free, unpaired cysteine in AMSIN-1 may confer a homodimer structure. This structure has been found to be functionally important in some defensins (Zhang *et al.* 2010. *Biochemistry* 49: 9770-9782; Song *et al.* 2011. *FEBS Lett* 585: 300-306); and the vicinal disulfide bridge (VD) in CBSIN-2 seems to alter the local folding. Since the most widespread biological role of VD is to provide a rigid hydrophobic contact surface under the undecorated side of a sugar or multiring ligand (Richardson *et al*. 2017. *J Mol Biol* 429: 1321-1335), the VD in CBSIN-2 could be also related to its function. Thus, on one hand, cysteine-based structural variations can extend the structural diversity of bacterial defensins, on the other hand they may be of certain functional significance.

Point 22. *Actinomycesin specifically inhibits the bacterial peptidoglycan synthesis and shows no toxicity on a panel of human ion channels relevant in the heart and the central nervous system, and mammalian cells. Why only heart and CNS were chosen?*

--AMSIN exhibits a similar structure to scorpion toxins, both adopting a common CSαβ fold and descent from a common ancestor (Zhu *et al*. 2014. *Mol Biol Evol* 31: 546-559). The ion channels chosen here are those frequently targeted by a variety of scorpion toxins for predation and defense (Korolkova *et al*. 2002. *J Biol Chem* 277: 43104-43109; Zhu *et al*.

2011. *Mol Cell Proteomics* 2011. 10: M110.002832; Zhu *et al*. 2012. *Mol Cell Proteomics* 11: M111.012054). They are expressed in the heart and CNS of mammals and humans with vital physiological functions (Wulff *et al.* 2009. *Nat Rev Drug Discov* 8: 982-1001; Jiang *et al.* 2020. *Cell* 180: 122-134). Intoxication of these channels by scorpion toxins can rapidly evoke paralysis and even death of scorpion's prey and competitors and thus represent important test targets for toxicity assays.

Point 23. *To check the therapeutic effect of the AMSIN gene, why only cell viability was checked? Other parameters can also be considered, like- the cell line can be Staphylococcus and Streptococcus in vivo and in vitro, and the effect of the AMSIN gene can be checked.*

--In this study, we evaluated the *in vitro* and *in vivo* activity of AMSIN and relevant therapeutic potential at the protein level. We agree that further consideration of its therapeutic effect at the gene level with AMSIN-sensitive bacterial strains is useful. However, based on our research focus here we feel that a detailed study of these issues may be beyond the scope of this study (please see our response to Point 15).

Point 24. *While determining the in vivo anti-infective potency of AMSIN, why the two mouse models of systemic infections- the mouse pneumonia model and the peritonitis model were chosen?*

--That we chose these two mouse models was based on two related references that reported the discovery and *in vivo* evaluation of the therapeutic efficacy of two newfound peptide antibiotics - plectasin from a fungus and teixobactin from uncultured bacteria (Mygind *et al.* 2005. *Nature* 437: 975-980; Ling *et al*. 2015. *Nature* 517: 455-459).

Point 25. *AMSIN was introduced intravenously (i.v.) one hour post-infection 20 mg per kg. This treatment continued for five days (once each day). How this concentration and time - period was determined?*

--We determined the doses of AMSIN and time - period in the mouse peritoneal model by reference to the literature ("Treatment with plectasin subcutaneously at 10 mg per kg was initiated our hour after inoculation.") (Mygind *et al*. 2005. *Nature* 437: 975-980) with slight modification, in which the first day at 20 mg per kg once (prim.vic.No2) and the second to the fifth day at 10 mg per kg once daily. AMSIN can achieve a maximal therapeutic efficacy in this dose regiment initially determined by a pre-experiment.

Point 26. *While assessing the effect of AMSIN on human ion channels expressed in the CNS and heart, the peptide dose used was 100 μM. Why 100 μM?*

--Because most scorpion toxins with a defensin fold work at a nanomolar or low micromolar concentration (Zhu *et al*. 2012. *Mol Cell Proteomics* 11: M111.012054; Wang *et al*. 2015. *Biochem Pharmacol* 93: 232-239), we chose a rather high dose of AMSIN to confirm its safety on the human ion channels relevant in the heart and the central nervous system.

Minor correction require:

Point 27. *The author used vague scientific terminology in their writing; they should have been more precise in their writing, as they often used words like antibiotics and AMSIN.*

--We have carefully gone through the manuscript with revisions to make sure no vague scientific terminology. To avoid the confusion, we have provided the citation concerning antimicrobial peptides also known as peptide antibiotics (Hancock 1997. Peptide antibiotics. *Lancet* 349: 418-422) (please see page 3: line 70).

Point 28. *In the material method section specify the animal species and ethical number.*

--We have specified the animal species used in this study. Animal experiments were performed according to procedures approved by the Ethics Committee of the Institute of Zoology, Chinese Academy of Sciences. The institute does not issue a number to any animal study, but each study requires the permit to use animals from the Ethics Committee (please see "*Animals" in the* Materials and methods *section*).

Point 29. *The author emphasises that "they had described 48 such peptides from two microbial subgroups (actinobacteria and myxobacteria belonging to deltaproteobacteria)," but they do not specify which isoform of AMSIN they are operating on throughout the manuscript. The author should state this.*

--Done (please see page 4: lines 116-118).

Point 30. *An author suggested submitting an English proficiency certificate for the concerned manuscript before final submission because the English language is not of good quality, and there is also wordiness.*

--To improve the quality of the English language, we have asked our colleagues who are skilled authors of English language papers to help check the language. Meanwhile, we have tried our best to revise the language to avoid wordiness and any grammar or syntax errors. We hope that the revised language is now acceptable for the next review process.

Point 31. *The author has written many too many long sentences, making this explanation challenging to comprehend. Below is a list of sentences that must be changed;*

31a. Actinomycesin and its homologs from actinobacteria and myxobacteria share similarity to a group of ancient invertebrate-type defensins from fungi and invertebrates, and those from actinomyces colonised in the human oral cavity and ruminant rumen and dental plaque are evolving under positive selection, supporting their role implicated in adaptation to these complex multispecies bacterial communities.

--Done (please see pages 1 and 2: lines 29-35).

31b. Defensins from actinomyces colonised in the human oral cavity and ruminant rumen and dental plaque other than from fungi and animals are evolving under positive Darwinian selection at their molecular surface, in favour of their role in ecological adaptation to the complex oral and rumen multispecies communities.

--Done (please see page 4: lines 119-122).

31c. Analysis of the intracellular pool of cell-wall precursors by means of reversed-phase high-performance liquid chromatography (RP-HPLC) combined with mass spectrometry revealed the accumulation of the soluble molecule UDP-MurNAc-pentapeptide (abbreviated as UMP) in AMSIN- or vancomycin-treated cells (Fig 4D), suggesting that the peptide may work in a similar manner to the inhibitors of cell-wall synthesis.

--Done (please see page 9: lines 280-284).

31d. We found that AMSIN exhibited a kinetic behaviour highly similar to vancomycin, a typical bacterial cell wall synthesis inhibitor, but differed from rifampicin that targets bacterial DNA-dependent RNA polymerase to inhibit RNA synthesis and kanamycin that works by interfering with protein synthesis (Fig 4A).

--Done (please see page 8: lines 266-269).

31e. UMP contains the elements responsible for direct interaction of Lipid II with plectasin (i.e. the pyrophosphate group and the γ-Glu in the pentapeptide) (Schneider et al 2010) or vancomycin (i.e. the D-Ala-D-Ala 20 motif) (Hernout et al, 2007) (Fig 4E; Fig EV13), thus allowing to investigate a possible interaction between AMSIN and Lipid II by using UMP as an alternative.

--Done (please see page 9: lines 284-289).

31f. As verified by AMSIN whose high efficacy and low toxicity as well as serum stability, together with a bacterial origin and potential complex microflora-driven adaptive evolution, all indicate that actinomycetes-derived defensins are promising Lipid II-bound antibiotic leads with greater therapeutic potential than their counterparts in eukaryotes.

--To improve the quality of Discussion, we have rewritten and extended these sentences and now they read "Therefore, defensins from oral or rumen actinomycetes are more likely leads than multifunctional eukaryotic defensins in drug design given that their antibacterial function has been optimized by complex microflora-driven natural selection, as mentioned previously. In this work, we have provided experimental evidence confirming that actinomycetes-derived defensins are indeed a class of promising peptide antibiotic leads targeting bacterial cell wall synthesis. As the representative, AMSIN lacked obvious cytotoxicity on mammalian and human cells and displayed weak immunomodulatory activity with relatively high serum stability (Fig 5). Of all of these properties relevant to therapeutic efficacy, AMSIN is superior to eukaryotic AMPs (e.g. human LL-37 and defensins). The latter often exhibits potent mammalian cytotoxicity with stronger immunomodulatory activity and serum instability and inactivation (Panyutich *et al*, 1995; Johansson *et al*, 1998; Scott *et al*, 2002; Hara *et al*, 2008; Zhu and Gao 2017; Kudryashova *et al*, 2021). From an evolutionary viewpoint, natural selection on mammalian LL-37 is associated with the evolutionary variability of their endogenous receptors mediating the immunomodulatory functions rather than bactericidal activity (Zhu and Gao 2017), which is different from the evolution of AMSIN and its bacterial homologs. This could explain the difference of the immunomodulatory activity between AMSIN and LL-37 and against consolidates the advantages of these bacterial defensins as anti-infective drugs relative to human AMPs." (please see page 12: lines 393-410).

Referee #3

Thank you very much for your positive comments and constructive suggestions on our work that are very valuable for improving our manuscript as well as guiding our future research. Please see below our point-to-point response to your issues:

Point 1. *Comments on Novelty/Model System for Author: The authors used both in silico, in vitro and in vivo system to characterize the functions of the molecule studied. Methods used are adequate however the use of T-test as statistical test should be justified. The topic of the study is quiet novel and with strong medical impact.*

--Regarding the use of T-test, please see our response to Point 4.

Point 2. *Remarks for Author: EMM-2021-14499*

The present study reports and characterized a human oral actinomyces-derived defensin (actinomycesin) with therapeutic potential. After in silico analyses of microbial-derived defensins, the authors biologically characterized actinomycesin. The authors demonstrated: using NOD mouse model and streptozotocin-induced model in C57BL/6 mice, the authors aim to demonstrate that:

-Actinomycesin shows a lytic effect on various pathogenic bacteria.

-Lytic effect synergizes with oral human antimicrobial peptides (alpha-defensin 1&2 and lysozyme C).

-Actinomycesin acts on bacteria by inhibiting bacterial cell wall biosynthesis.

-Actinomycesin has anti-infective potency in mouse models of infection (pneumonia and peritonitis).

The present study provides important insights to the current knowledge but the authors need to provide additional data to strengthen their conclusion. The authors need to further characterize the potential toxicity of actinomycesin in vivo via different routes of administration in animal models.

--In the two *in vivo* animal models reported here, AMSIN was administrated via two different routes, intramuscular injection (i.m.) for the pneumonia model and intravenous injection (i.v.) for the peritonitis model. We found that both created no obvious toxicity. Because the focus of this work was: (1) to discover of bacterial counterparts of eukaryotic AMPs; (2) to study their evolution and structural diversity; (3) to characterize their interacting targets; and (4) to evaluate their therapeutic potential, a detailed assessment of the

toxicity originated from different routes of administration could be beyond the scope of this study. However, your instructive suggestions are valuable to our next-step research to develop AMSIN into a clinical drug. Thank you for your understanding.

Point 3. *Regarding the growing knowledge regarding the immunomodulatory roles of defensins, this aspect need to be investigate almost in vitro (chemotactic activity, pro- or anti-inflammatory activity...).*

--In this revised version, we have added new experimental data to evaluate the potential immunomodulatory role of AMSIN by measuring the chemokine interleukin-8 (IL-8) release by A549 cells (a human alveolar type II cell line) as a representative. We found that AMSIN did not induce an elevated IL-8 release at a concentration range (25-100 μg/mL) in a FBS-containing medium ($P>0.05$). Although AMSIN showed some activity of inducing IL-8 release when the cells were cultured in the medium without the serum, the activity was generally weaker than that of LL-37 (please see Figure 5F and the associated text on page 10: lines 331-340).

Point 4. *The number of samples analyzed and the number of experiments need to be mentioned in the figure legends. Indeed, this is mandatory to determine if the T-test used by the authors is adapted to analyze the data. T-test requires 20 independent values per group. If it is not the case, a non-parametric Mann-Whitney test must be used.*

--We have added the number of samples analyzed and the number of experiments in the figure legends. In this revised version, our data are expressed as mean ± standard deviation (SD) ($10 \geq n \geq 3$) which were calculated by Excel. Statistical significance between two groups was determined by unpaired two-tailed Student's t-test for the data that accord with normal distribution and homogeneity of variance, as reported in other studies (e.g. Monteil *et al.* 2020. *Cell* 181: 905-913: “Statistics: Statistical analysis was conducted using GraphPad Prism 8 (GraphPad) and significance was determined by Student’s test”). For the data that did not meet any one of the two conditions, we used the nonparametric Mann-Whitney U test to compare the difference between two groups (Hoffman 2015. *Biostatistics for Medical and Biomedical Practitioners*, Academic Press, pp. 337-362), as recommended by the reviewer. Before these two tests, we firstly tested data for normal distribution by Shapiro-Wilk test and homogeneity of variance by Levene’s test (please see “Statistics” in the Materials and methods section).

3rd Nov 2021

Dear Prof. Zhu,

Thank you for the submission of your revised manuscript to EMBO Molecular Medicine. We have now received the enclosed reports from the two referees who re-reviewed your manuscript. As you will see, they are now supportive of publication, and I am therefore pleased to inform you that we will be able to accept your manuscript, once the following minor points will be addressed:

1/ Please address the remaining minor concern from referee #3.

2/ Main manuscript text
- Please address the queries from our data editors in track changes mode in the main manuscript file labelled 'Data edited ms file'. Please use this file for any further modification.
- Please remove the red text and only keep in track changes mode any new modification.
- We usually recommend a maximum of 175 words for abstracts. While this is not a strict limit, we would appreciate if you could shorten your abstract.
- We can accommodate a maximum of 5 keywords, please adjust accordingly.
- Material and methods:
o Cells: please indicate the origin of the cells, and whether they were tested for mycoplasma contamination.
o Animals: please indicate the age and gender of the mice for each type of experiment.
- Thank you for providing a Data Availability Section. Please place it directly after the Material and Methods section. Please indicate in this section all datasets generated in this study that need to be deposited in a public repository (including RNAseq data) and provide an URL for each. Please only list here the datasets newly generated for this study (and not yet published).

3/ Figures:
- EV figures need to be uploaded as individual, high-resolution figure files. EV tables need to be uploaded as individual files, one table per file.
- Please upload the different datasets as individual excel files, one tab per table.

4/ Checklist:
- please provide information in section C-7. In particular, please indicate whether the cells were tested for mycoplasma contamination.
- please provide information on the gender and the age of the mice in section D -8
- please fill the section F according to the Data Availability section (in particular, please deposit the RNAseq data in a public repository).

5/ Thank you for providing Source Data. Please upload them as 1 file per figure.

6/ Thank you for providing The Paper Explained. I added minor edits, please let me know if you agree with the following, or amend as you see fit:
Problem
Despite their evolutionary success as eukaryotic defensive weapon across 1.8-billion-year history and intense research over several decades, few eukaryote-sourced antimicrobial peptides (AMPs) have reached the stage of clinical applications for treating systemic infection. Two main intrinsic factors hamper the development of these AMPs as systemically administrated drugs. First, in eukaryotes, especially in multicellular organisms, most AMPs defend a microenvironment and are not delivered into the systemic circulation; second, in multicellular organisms, AMPs have evolved multiple biological functions beyond their antibiotic activity. These additional properties often interfere with the human immune system function when administrated systemically.
Results
Our study addressed these two critical issues based on the hypothesis that AMPs from unicellular, prokaryotic organisms that are the most evolutionary distant from humans, may exhibit more advantages than AMPs from multicellular organisms. We searched for AMPs from the public available microbial genome resources and discovered AMSIN, the first human oral actinomyces-sourced defensin targeting bacterial cell-wall biosynthesis and exhibiting therapeutic potential against bacterial infections elicited by Streptococcus pneumoniae and methicillin-resistant Staphylococcus aureus in mice.
Impact
This work identifies human oral bacteria as a new source of peptide antibiotics, and might stimulate a shift in this field of research from eukaryotes to prokaryotes.

7/ Thank you for providing relevant web links. Please rename this section "For more information". The links to your deposited data should be removed and listed in the Data Availability Section. Furthermore, we could not access the following link: http://www.cbs.dtu.dk/services/SecretomeP/

8/ Thank you for providing a synopsis. Please adjust the text to fit our format and style:
The synopsis should include a short stand first (maximum of 300 characters, including space) as well as 2-5 one-sentences bullet points that summarizes the paper. Please write the bullet points to summarize the key NEW findings. They should be designed to be complementary to the abstract - i.e. not repeat the same text. We encourage inclusion of key acronyms and quantitative information (maximum of 30 words / bullet point).

Please also reformat the synopsis image to a png/tif/jpeg file 550 px wide x 300-600 px high. Make sure that the text remains legible.

9/ As part of the EMBO Publications transparent editorial process initiative (see our Editorial at http://embomolmed.embopress.org/content/2/9/329), EMBO Molecular Medicine will publish online a Review Process File (RPF) to accompany accepted manuscripts.
This file will be published in conjunction with your paper and will include the anonymous referee reports, your point-by-point response and all pertinent correspondence relating to the manuscript. Let us know whether you agree with the publication of the RPF and as here, if you want to remove or not any figures from it prior to publication.
Please note that the Authors checklist will be published at the end of the RPF.

I look forward to receiving your revised manuscript.

Yours sincerely,

Lise Roth

Lise Roth, PhD
Editor
EMBO Molecular Medicine

To submit your manuscript, please follow this link:

Link Not Available

The system will prompt you to fill in your funding and payment information. This will allow Wiley to send you a quote for the article processing charge (APC) in case of acceptance. This quote takes into account any reduction or fee waivers that you may be eligible for. Authors do not need to pay any fees before their manuscript is accepted and transferred to our publisher.

***** Reviewer's comments *****

Referee #2 (Comments on Novelty/Model System for Author):

I have no concerns.

Referee #2 (Remarks for Author):

There are no concerns since the authors have integrated and offered sufficient responses to the questions that were expressed. Following a thorough examination of the whole manuscript, I am satisfied with the current revision.

Referee #3 (Remarks for Author):

The overall revised manuscript has been improved; however, the authors may more extensively evaluate the immune aspects of AMSIN. the authors show only that in vitro AMSIN induces IL-8 expression by A549 cells. Other cytokines may be measured almost in this in vitro system, although evaluating the level of cytokines in the serum of mice after AMSIN treatment does not represent technically challenging experiments.

EMM-2021-14499

Adaptively Evolved Human Oral Actinomyces-Sourced Defensins Show Therapeutic Potential

Response to *EMBO Molecular Medicine* Reviewers Comments

Referee #2 (Comments on Novelty/Model System for Author):

I have no concerns.

Referee #2 (Remarks for Author):

There are no concerns since the authors have integrated and offered sufficient responses to the questions that were expressed. Following a thorough examination of the whole manuscript, I am satisfied with the current revision.

-- We have performed a thorough examination of the whole manuscript. Thanks.

Referee #3 (Remarks for Author):

The overall revised manuscript has been improved; however, the authors may more extensively evaluate the immune aspects of AMSIN. the authors show only that in vitro AMSIN induces IL-8 expression by A549 cells. Other cytokines may be measured almost in this in vitro system, although evaluating the level of cytokines in the serum of mice after AMSIN treatment does not represent technically challenging experiments.

-- Thank you for your comments. In this study, we chose IL-8 as a representative of cytokines to study the immunomodulatory activity of AMSIN based on several previous observations: (1) IL-8 is a chemotactic and inflammatory cytokine and the prototype of the CXC chemokine family (Baggiolini and Clark-Lewis 1992. *FEBS Lett* 307: 97-101). (2) The elevated expression of IL-8 is often associated with some inflammatory diseases and tumor progression (Alfaro *et al*, 2017. *Cancer Treat Rev* 60: 24-31; Ha *et al*, 2017. *Theranostics* 7: 1543-1588). (3) IL-8 has been used as an index to evaluate the immunomodulatory activity (potential toxicity) of AMPs (e.g. Hara *et al*, 2008. *Biochem Biophys Res Commun* 374: 709-713). We agree with your comment that a more extensive evaluation of responses of multiple cytokines to AMSIN both *in vitro* and *in vivo* could be beneficial in further

exploiting the therapeutic value of this peptide, however, we feel that more work in this aspect is beyond the scope of this study. Please see our response to this comment on page 10: lines 327–334 in the revised text.

10th Nov 2021

Dear Prof. Zhu,

Thank you for providing the revised files.
Before we can proceed with acceptance, please address the following remaining minor concerns:

1/ Please address experimentally (at least in vitro) the remaining minor concern from referee #3 regarding the immune aspects of AMSIN.

2/ Main manuscript text
- I slightly modified your abstract, please let me know if you agree with the following:
The development of eukaryote-derived antimicrobial peptides as systemically administered drugs has proven a challenging task. Here, we report the first human oral actinomyces-sourced defensin - actinomycesin - that shows promise for systemic therapy. Actinomycesin and its homologs are only present in actinobacteria and myxobacteria, and share similarity with a group of ancient invertebrate-type defensins reported in fungi and invertebrates. Signatures of natural selection were detected in defensins from the actinomyces colonized in human oral cavity and ruminant rumen and dental plaque, highlighting their role in adaptation to complex multispecies bacterial communities. Consistently, actinomycesin exhibited potent antibacterial activity against oral bacteria and clinical isolates of Staphylococcus and synergized with two classes of human salivary antibacterial factors. Actinomycesin specifically inhibited bacterial peptidoglycan synthesis and displayed weak immunomodulatory activity and low toxicity on mammalian cells and ion channels in the heart and the central nervous system. Actinomycesin was highly efficient in mice infected with Streptococcus pneumoniae and mice with MRSA-induced experimental peritoneal infection. This work identifies human oral bacteria as a new source of systemic anti-infective drugs.
- Thank you for providing a Data Availability Section. Please remove "Other data underlying this article are available in the article and in its online Expanded View, Appendix and Dataset EV." Please only list here the datasets newly generated for this study (the chemical shift data and coordinates of AMSIN seem to have been published before (https://bmrb.io/data_library/summary/?bmrbId=11537 and https://www.rcsb.org/structure/2RU0), please clarify).

3/ Thank you for providing a synopsis. I added minor edits, please let me know if you agree with the following, or amend as you see fit:

Bacteria are a major source of clinically used antibiotics. This study explored the potential of oral actinomyces-sourced defensins as systemic anti-infective drugs via a combination of microbial genome mining, evolution analysis and experimental studies.
- Bacterial defensins with the cysteine-stabilized α-helical and β-sheet motifs are exclusively present in actinomycetes and myxobacteria
- AMSIN kills sensitive bacteria through interaction with Lipid II to hamper cell-wall biosynthesis
- AMSIN shows low toxicity and antibacterial activity in undiluted human serum, which is superior to some human-derived host defense peptides

Once revised, please remove the red text and accept all changes.

I look forward to receiving your revised manuscript.

Yours sincerely,

Lise Roth

Lise Roth, PhD
Editor
EMBO Molecular Medicine

EMM-2021-14499

Adaptively Evolved Human Oral Actinomyces-Sourced Defensins Show Therapeutic Potential

Response to *EMBO Molecular Medicine* Reviewers Comments

Referee #3 (Remarks for Author):

The overall revised manuscript has been improved; however, the authors may more extensively evaluate the immune aspects of AMSIN. the authors show only that in vitro AMSIN induces IL-8 expression by A549 cells. Other cytokines may be measured almost in this in vitro system, although evaluating the level of cytokines in the serum of mice after AMSIN treatment does not represent technically challenging experiments.

-- Thank you for your comments. In this revised version, we used a human immune-related cell line (HL-60) to evaluate its cytokine response to AMSIN. HL-60 is a human neutrophil-like cell line commonly used to study neutrophil phenotypic functions (Millius and Weiner. 2009. *Methods Mol Biol* 571: 167-177). It is known that this cell line can constitutively produce monocyte chemotactic protein-1 (MCP-1) protein (about 400 pg/mL) (Steube *et al*, 1999. *Leuk Res* 23: 843-849). This cytokine is the prototype of β-chemokines (the CC family) (Deshmane *et al*, 2009. *J Interferon Cytokine Res* 29: 313-326) and its elevated expression is associated with various human diseases (Deshmane *et al*, 2009). We thus chose the HL-60 – MCP-1 system to further investigate the immune-modulatory effect of AMSIN on human cells. The results show that in the serum-containing medium, AMSIN did not significantly enhance the release of MCP-1 by HL-60 at the concentration range (25-100 μg/mL) when compared with the control. This data provides new evidence for our initial conclusion drawn from the A549 – IL-8 system, that is, AMSIN has a rather weak immunomodulatory effect on human cells (please see Fig 5G and its legend and the associated text on pages 10 and 11: lines 333-349 as well as the Methods section "Measurement of peptide-induced cytokine release in human-derived cell lines").

3rd Dec 2021

Dear Prof. Zhu,

Thank you for bearing with the last revisions. We are pleased to inform you that your manuscript is accepted for publication and is now being sent to our publisher to be included in the next available issue of EMBO Molecular Medicine!

We would like to remind you that as part of the EMBO Publications transparent editorial process initiative, EMBO Molecular Medicine will publish a Review Process File online to accompany accepted manuscripts. If you do NOT want the file to be published or would like to exclude figures, please immediately inform the editorial office via e-mail.

To better promote your work among the Chinese readership, we would like to invite you to prepare a short summary of the manuscript in Chinese, which we will promote on the WeChat platform 'BioArt' with 240,000 followers.

If you are interested in taking up this opportunity, we recommend to cover the article very close to its online publication date. Thus, ideally we will very much appreciate it if you can send us a draft by replying to this invitation email within the next 7 working days. My colleague Jingyi (jingyi.hou@embo.org) will be happy to suggest any edits that may help to further improve the text. Please let us know within 3 days if you plan (or not) to contribute such a short summary in Chinese.

Below please find some general guidelines on how to prepare a summary. I have also included links to recent examples for your reference.

Thank you again and please let me know if you have any question.

Congratulations on your interesting work!

With kind regards,

Lise Roth

Lise Roth, Ph.D
Scientific Editor
EMBO Molecular Medicine

Follow us on Twitter @EmboMolMed
Sign up for eTOCs at embopress.org/alertsfeeds

EMBO PRESS

YOU MUST COMPLETE ALL CELLS WITH A PINK BACKGROUND ➔

PLEASE NOTE THAT THIS CHECKLIST WILL BE PUBLISHED ALONGSIDE YOUR PAPER

Corresponding Author Name: Shunyi Zhu
Journal Submitted to: EMBO Molecular Medicine
Manuscript Number: EMM-2021-14499

Reporting Checklist For Life Sciences Articles (Rev. June 2017)

This checklist is used to ensure good reporting standards and to improve the reproducibility of published results. These guidelines are consistent with the Principles and Guidelines for Reporting Preclinical Research issued by the NIH in 2014. Please follow the journal's authorship guidelines in preparing your manuscript.

A- Figures
1. Data
The data shown in figures should satisfy the following conditions:

- ➔ the data were obtained and processed according to the field's best practice and are presented to reflect the results of the experiments in an accurate and unbiased manner.
- ➔ figure panels include only data points, measurements or observations that can be compared to each other in a scientifically meaningful way.
- ➔ graphs include clearly labeled error bars for independent experiments and sample sizes. Unless justified, error bars should not be shown for technical replicates.
- ➔ if n< 5, the individual data points from each experiment should be plotted and any statistical test employed should be justified
- ➔ Source Data should be included to report the data underlying graphs. Please follow the guidelines set out in the author ship guidelines on Data Presentation.

2. Captions
Each figure caption should contain the following information, for each panel where they are relevant:

- ➔ a specification of the experimental system investigated (eg cell line, species name).
- ➔ the assay(s) and method(s) used to carry out the reported observations and measurements
- ➔ an explicit mention of the biological and chemical entity(ies) that are being measured.
- ➔ an explicit mention of the biological and chemical entity(ies) that are altered/varied/perturbed in a controlled manner.

- ➔ the exact sample size (n) for each experimental group/condition, given as a number, not a range;
- ➔ a description of the sample collection allowing the reader to understand whether the samples represent technical or biological replicates (including how many animals, litters, cultures, etc.).
- ➔ a statement of how many times the experiment shown was independently replicated in the laboratory.
- ➔ definitions of statistical methods and measures:
  - common tests, such as t-test (please specify whether paired vs. unpaired), simple χ2 tests, Wilcoxon and Mann-Whitney tests, can be unambiguously identified by name only, but more complex techniques should be described in the methods section;
  - are tests one-sided or two-sided?
  - are there adjustments for multiple comparisons?
  - exact statistical test results, e.g., P values = x but not P values < x;
  - definition of 'center values' as median or average;
  - definition of error bars as s.d. or s.e.m.

Any descriptions too long for the figure legend should be included in the methods section and/or with the source data.

In the pink boxes below, please ensure that the answers to the following questions are reported in the manuscript itself. Every question should be answered. If the question is not relevant to your research, please write NA (non applicable). We encourage you to include a specific subsection in the methods section for statistics, reagents, animal models and human subjects.

USEFUL LINKS FOR COMPLETING THIS FORM

http://www.antibodypedia.com
http://1degreebio.org
http://www.equator-network.org/reporting-guidelines/improving-bioscience-research-repor
http://grants.nih.gov/grants/olaw/olaw.htm
http://www.mrc.ac.uk/Ourresearch/Ethicsresearchguidance/Useofanimals/index.htm
http://ClinicalTrials.gov
http://www.consort-statement.org
http://www.consort-statement.org/checklists/view/32-consort/66-title
http://www.equator-network.org/reporting-guidelines/reporting-recommendations-for-tumo
http://datadryad.org
http://figshare.com
http://www.ncbi.nlm.nih.gov/gap
http://www.ebi.ac.uk/ega
http://biomodels.net/
http://biomodels.net/miriam/
http://jjj.biochem.sun.ac.za
http://oba.od.nih.gov/biosecurity/biosecurity_documents.html
http://www.selectagents.gov/

B- Statistics and general methods

Please fill out these boxes ➔ (Do not worry if you cannot see all your text once you press return)

1.a. How was the sample size chosen to ensure adequate power to detect a pre-specified effect size?	Sample size calculations were based on literatures.
1.b. For animal studies, include a statement about sample size estimate even if no statistical methods were used.	No statistical methods were used to predetermine sample size. For animal experiments at least 3 animals were used per group.
2. Describe inclusion/exclusion criteria if samples or animals were excluded from the analysis. Were the criteria pre-established?	No data were excluede from the analiysis.
3. Were any steps taken to minimize the effects of subjective bias when allocating animals/samples to treatment (e.g. randomization procedure)? If yes, please describe.	N/A.
For animal studies, include a statement about randomization even if no randomization was used.	Mice were randomly divided into the distinct groups.
4.a. Were any steps taken to minimize the effects of subjective bias during group allocation or/and when assessing results (e.g. blinding of the investigator)? If yes please describe.	N/A.
4.b. For animal studies, include a statement about blinding even if no blinding was done	Data collection and analysis were not performed blind.
5. For every figure, are statistical tests justified as appropriate?	Yes.
Do the data meet the assumptions of the tests (e.g., normal distribution)? Describe any methods used to assess it.	Statistical analyses were carried out using SPSS Statistics 17.0 (SPSS Inc.). Data are expressed as mean ± standard deviation (SD) (10≥n≥3) which were calculated by Excel. Statistical significance between two groups was determined by unpaired two-tailed Student's t-test for the data that accorded with normal distribution and homogeneity of variance. For the data that did not meet any one of the two conditions, we used the nonparametric Mann-Whitney U test to compare the difference between two groups (Hoffman 2015). Before these two tests, we firstly tested data for normal distribution by Shapiro-Wilk test and homogeneity of variance by Levene's test. Generally, P≤0.05 was considered statistically significant (*), P≤0.01 was considered highly significant (**) and P≤ 0.001 was considered very highly significant (***)
Is there an estimate of variation within each group of data?	Data are expressed as mean ± standard deviation (SD) from three independent experiments.

Is the variance similar between the groups that are being statistically compared?	Yes.

C- Reagents

6. To show that antibodies were profiled for use in the system under study (assay and species), provide a citation, catalog number and/or clone number, supplementary information or reference to an antibody validation profile. e.g., Antibodypedia (see link list at top right), 1DegreeBio (see link list at top right).	N/A.
7. Identify the source of cell lines and report if they were recently authenticated (e.g., by STR profiling) and tested for mycoplasma contamination.	A549 cells were purchased from the Cell Resource Center of the Chinese Academy of Medical Sciences (Beijing, China). They were tested for mycoplasma contamination with the Mycoplasma Detection Kit (Solarbio, China) and only negative cultures were used in our experiments.

* for all hyperlinks, please see the table at the top right of the document

D- Animal Models

8. Report species, strain, gender, age of animals and genetic modification status where applicable. Please detail housing and husbandry conditions and the source of animals.	Institute of Cancer Research (ICR) mice (Mus musculus) were purchased from (SPF (Beijing) Biotechnology Co., Ltd., Beijing, China). Animals were housed in a temperature-controlled room (22-25°C) with water and food available ad libitum, and on a 12 h light/dark cycle. Female ICR mice: 6 or 9-10 weeks of age; Male ICR mice: 6 weeks of age.
9. For experiments involving live vertebrates, include a statement of compliance with ethical regulations and identify the committee(s) approving the experiments.	Animal experiments were performed according to procedures approved by the Ethics Committee of the Institute of Zoology, Chinese Academy of Sciences.
10. We recommend consulting the ARRIVE guidelines (see link list at top right) (PLoS Biol. 8(6), e1000412, 2010) to ensure that other relevant aspects of animal studies are adequately reported. See author guidelines, under 'Reporting Guidelines'. See also: NIH (see link list at top right) and MRC (see link list at top right) recommendations. Please confirm compliance.	We confirm compliance.

E- Human Subjects

11. Identify the committee(s) approving the study protocol.	N/A.
12. Include a statement confirming that informed consent was obtained from all subjects and that the experiments conformed to the principles set out in the WMA Declaration of Helsinki and the Department of Health and Human Services Belmont Report.	N/A.
13. For publication of patient photos, include a statement confirming that consent to publish was obtained.	N/A.
14. Report any restrictions on the availability (and/or on the use) of human data or samples.	N/A.
15. Report the clinical trial registration number (at ClinicalTrials.gov or equivalent), where applicable.	N/A.
16. For phase II and III randomized controlled trials, please refer to the CONSORT flow diagram (see link list at top right) and submit the CONSORT checklist (see link list at top right) with your submission. See author guidelines, under 'Reporting Guidelines'. Please confirm you have submitted this list.	N/A.
17. For tumor marker prognostic studies, we recommend that you follow the REMARK reporting guidelines (see link list at top right). See author guidelines, under 'Reporting Guidelines'. Please confirm you have followed these guidelines.	N/A.

F- Data Accessibility

18: Provide a "Data Availability" section at the end of the Materials & Methods, listing the accession codes for data generated in this study and deposited in a public database (e.g. RNA-Seq data: Gene Expression Omnibus GSE39462, Proteomics data: PRIDE PXD000208 etc.) Please refer to our author guidelines for 'Data Deposition'.<br><br>Data deposition in a public repository is mandatory for:<br>a. Protein, DNA and RNA sequences<br>b. Macromolecular structures<br>c. Crystallographic data for small molecules<br>d. Functional genomics data<br>e. Proteomics and molecular interactions	The chemical shift data and coordinates of AMSIN were deposited to BioMagResBank (BMRB Entry 11537; https://bmrb.io/data_library/summary/?bmrbId=11537) and Protein Data Bank (accession number 2RU0; https://www.rcsb.org/structure/2RU0), respectively. RNAseq data that included the transcriptional response of SP R6 cells to AMSIN or vancomycin at a subinhibitory concentration and the transcriptional profile of the untreated cells were deposited to the Genbank database (accession number PRJNA778040; https://www.ncbi.nlm.nih.gov/sra/?term=PRJNA778040).
19. Deposition is strongly recommended for any datasets that are central and integral to the study; please consider the journal's data policy. If no structured public repository exists for a given data type, we encourage the provision of datasets in the manuscript as a Supplementary Document (see author guidelines under 'Expanded View' or in unstructured repositories such as Dryad (see link list at top right) or Figshare (see link list at top right).	All seqeunce data underlying this article are available in the article and in its online Expanded View.
20. Access to human clinical and genomic datasets should be provided with as few restrictions as possible while respecting ethical obligations to the patients and relevant medical and legal issues. If practically possible and compatible with the individual consent agreement used in the study, such data should be deposited in one of the major public access-controlled repositories such as dbGAP (see link list at top right) or EGA (see link list at top right).	N/A.
21. Computational models that are central and integral to a study should be shared without restrictions and provided in a machine-readable form. The relevant accession numbers or links should be provided. When possible, standardized format (SBML, CellML) should be used instead of scripts (e.g. MATLAB). Authors are strongly encouraged to follow the MIRIAM guidelines (see link list at top right) and deposit their model in a public database such as Biomodels (see link list at top right) or JWS Online (see link list at top right). If computer source code is provided with the paper, it should be deposited in a public repository or included in supplementary information.	N/A.

G- Dual use research of concern

22. Could your study fall under dual use research restrictions? Please check biosecurity documents (see link list at top right) and list of select agents and toxins (APHIS/CDC) (see link list at top right). According to our biosecurity guidelines, provide a statement only if it could.	N/A.
